# Supplementary material for: Strategies to identify individuals with monogenic diabetes: results of an economic evaluation
Source: BMJ Open. 2020 Mar 18;10(3):e034716. doi: 10.1136/bmjopen-2019-034716 (PMC7150598; doi:10.1136/bmjopen-2019-034716)
Supplement: Supplementary data [file bmjopen-2019-034716supp001.pdf]

Supplementary Data 1: Parameters and results for Cohort 2

Cohort 2 - Diagnosed with diabetes <30yrs old and still <30 yrs old at start of model

Table 1A Characteristics of the modelled Cohort 2 at entry to the model

Characteristic	Parameter value	Evidence source
Prevalence (95% confidence interval)		
GCK mutation	1·2% (0.5%, 2.3%)	Shields et al[1] & unpublished data from accompanying clinical study (N=687)
HNF1A mutation	0·9% (0.3%, 1.9%)	Shields et al[1] & unpublished data from accompanying clinical study (N=687)
HNF4A mutation	0.1% (0%, 0.5%)	Shields et al[1] & unpublished data from accompanying clinical study (N=687)
Type 1 diabetes[a]	93·4% (91.3%, 95.2%)	Unpublished data from accompanying clinical study (N=687)
Type 2 diabetes	4·5% (3.1%, 6.3%)	Unpublished data from accompanying clinical study (N=687)
Age (years) [b]	19	Unpublished data from accompanying clinical study (N=687)
Time since diagnosis (years) [b]	8	
Body mass index [b]	25·7	
HbA1c (mmol/mol) [b]	59.8	
Female	50%	
Systolic blood pressure [b]	131·7	[2]
Total cholesterol [b]	4·74	[2]
High density lipoprotein [b]	1·31	[2]
Low density lipoprotein [b]	2·61	[2]
Triglycerides [b]	0·83	[2]
Caucasian	89%	[3]

Black	4%	3
Asian	7%	3

[a] Defined as receiving insulin treatment within 12 months of diabetes diagnosis.
[b]Mean.

Table 1B Percentage (95% CI) of referred individuals tested for mutations in *GCK* and/or *HNF1A* and *HNF4A* genes by true diagnosis (from unpublished UK referral centre data)

True diabetes diagnosis	Percentage (95% CI) [N=1399]		
	***GCK* only**	***HNF1A* and *HNF4A***	***GCK*, *HNF1A* and *HNF4A***
Not monogenic	15.8% (13.4%, 18.4%)	69.0% (65.8%, 72.0%)	15.2% (12.9%, 17.8%)
GCK mutation	94.6% (91.0%, 97.1%)		5.3% (2.9%, 9.0%)
HNF1A mutation		95.0% (91.0%, 97.6%)	5.0% (2.4%, 9.0%)
HNF4A mutation		96.4% (89.8%, 99.2%)	3.6% (0.8%, 10.2%)

Table 1C Percentage (95% CI) of cohort not accepting offer of testing, or requiring multiple tests for the Biomarker Testing strategy

	Percentage (95% CI)	
Number of tests	UCPCR (including urine sample) N=1299	Autoantibody (including blood sample) N=419
0	12·8% (11.0%, 14.7%)	6·9% (4.7%, 9.8%)
1	84·6% (82.5%, 86.5%)	90·5% (87.2%, 93.1%)
2	2·4% (1.6%, 3.4%)	2·6% (1.3%, 4.6%)
3	0·1% (0.04%, 0.7%)	0%

UCPCR, urinary c-peptide creatinine ratio. Unpublished data from accompanying clinical study.

Table 1D Multipliers (and 95% confidence intervals) to inform cascade genetic testing of diabetic family members

Number of relatives test per true monogenic diabetes case identified	Cohort 2 multiplier	Data source
Relatives positive for monogenic diabetes	5.6 (4.7, 6.5)	Re-analysis of Shields et al[4] (specific to definition of modelled cohort)
Relatives negative for monogenic diabetes	0.6 (0.3, 1.0)	

Peters JL, *et al. BMJ Open* 2020; 10:e034716. doi: 10.1136/bmjopen-2019-034716

Table 1E Pre-genetic treatment pattern, cost and frequency of HBGM by true diagnosis

	Treatment	% receiving treatment	Mean monthly treatment costs	Mean frequency of HBGM[a]
Type 1	Insulin only	100%	£52	78
Type 2	Insulin only	0%	£55	43
	Insulin + tablets	19%	£50	43
	Tablets only	68%	£2	17
	No diabetes treatment	13%	£0	0
GCK	Insulin only	75% (19%, 99%)	£5	52 (0, 110)
	Tablets only	25% (0.6%, 81%)	£1	0
HNF1A or *HNF4A*	Insulin only	67% (35%, 90%)	£18	63 (37, 90)
	Insulin + tablets	0%		
	Tablets	25.0% (6%, 57%)	£1	
	No diabetes treatment	8% (0.2%, 38%)	£0	0

[a]HBGM, home blood glucose monitoring

Table 1F Post-diagnosis HBGM frequency by treatment changed to and true diagnosis

	Time since diagnosis of monogenic diabetes			
	**1 month**	**3 months**	**6 months**	**12 months**
GCK – no diabetes treatment	0	0	0	0
HNF1A and *HNF4A* – tablets only	41 (19, 62)	23 (5, 41)	19 (6, 33)	16 (3, 28)

Table 1G Percentage of individuals with *HNF1A* or *HNF4A* mutations changing to more appropriate treatment after receiving a diagnosis of monogenic diabetes

	Time since treatment change (month)			
	1	3	6	12
Percentage changing to more appropriate treatment	100% (73%, 100%)	100% (73%, 100%)	100% (73%, 100%)	100% (73%, 100%)

Table 1H Summary of base case, sensitivity and threshold analyses

Parameter	Base case justification	Justification of sensitivity/threshold analyses
Long-term insulin need for individuals with HNF1A or HNF4A mutations	Expert 1	Expert 2, who assumed greater insulin need sooner.
Prevalence of monogenic diabetes	In the accompanying clinical study, the total number of cases of monogenic diabetes was 14 from a total of 687 individuals screened. This leads to an estimated prevalence within the definition of Cohort 1 of 14/687 = 2%.	In sensitivity analyses it was assumed that:<br>1. all of the remaining 993 who were eligible to be screened in the accompanying clinical study would fit the definition for Cohort 2, but were not cases of monogenic diabetes, therefore a lower prevalence of monogenic diabetes was assumed (14/1670 = 0.8%).<br>2. as an upper limit, the prevalence of monogenic diabetes was doubled (28/687 = 4%).
Sensitivity and specificity of the Ad Hoc Testing strategy	Based on referral rate data for Northern Ireland (the region with the lowest referral rates)[4]	Analysed all regions using estimates of sensitivity and specificity given in Supplementary Data 3.
Genetic test cost	UK referral centre costs[5]: £350 for *GCK* mutation; £450 for *HNF1A* and *HNF4A* mutations.	Threshold analyses to identify at what cost of the *GCK* and *HNF1A* and *HNF4A* genetic tests would the All Tested strategy incur no additional costs over the No Testing strategy. Costs of tests for *GCK* and *HNF1A* and *HNF4A* mutations were reduced in 10% steps to just 10% of their base case costs: £35 for *GCK* and £45 for *HNF1A* and *HNF4A*.
Uptake of UCPCR test	Based on data from the accompanying clinical study which investigated the application of the Biomarker Testing strategy.<br>Uptake of UCPCR was assumed to be 87%.	Threshold analyses where UCPCR test uptake was assumed to range from 100% to just 10%.<br>It was hypothesised that test uptake in practice is likely to be lower than test uptake in the accompanying clinical study where individuals have consented to participating in a study.
Uptake of autoantibody test	Based on data from the accompanying clinical study which investigated the application of the Biomarker Testing strategy.<br>Uptake of autoantibody testing was assumed to be 93%.	Threshold analyses where autoantibody test uptake was assumed to range from 100% to just 10%.<br>It was hypothesised that test uptake in practice is likely to be lower than test uptake in the accompanying clinical study where individuals have consented to participating in a study
Uptake of genetic test	Based on data from the accompanying clinical study which investigated the application of the Biomarker Testing strategy.<br>Uptake of genetic testing was assumed to be the same as for autoantibody testing (93%) since the same blood sample for autoantibody testing was used for the genetic testing.	Threshold analyses where genetic test uptake was assumed to range from 100% to just 10%.<br>It was hypothesised that test uptake in practice is likely to be lower than test uptake in the accompanying clinical study where individuals have consented to participating in a study
Repeat urine samples and UCPCR tests	Based on data from the accompanying clinical study which investigated the	Threshold analyses were undertaken assuming no repeats, 1%, 5%, 10%, 20%, 50%, 100%, 150% and 200% of samples and tests needed to be repeated. 200%

	application of the Biomarker Testing strategy. The percentage of repeat urine samples and UCPCR tests was assumed to be 3%.	repeat samples and tests can be interpreted as every individual requiring another 2 urine samples and UCPCR tests to be done, so that in total every individual has provided 3 urine samples and 3 UCPCR tests have been done – an extreme assumption.
Repeat blood samples and autoantibody tests	Based on data from the accompanying clinical study which investigated the application of the Biomarker Testing strategy. The percentage of repeat blood samples and autoantibody tests was assumed to be 3%.	Threshold analyses were undertaken assuming no repeats, 1%, 5%, 10%, 20%, 50%, 100%, 150% and 200% of samples and tests needed to be repeated. 200% repeat samples and tests can be interpreted as every individual requiring another 2 blood samples and autoantibody tests to be done, so that in total every individual has provided 3 blood samples and 3 autoantibody tests have been done – an extreme assumption.
Sensitivity of UCPCR test	Based on data from Besser et al[6] which used a prevalent case-control diagnostic study design: 0.94.	Since the sensitivity estimate for the UCPCR test is from a case-control diagnostic study, it is likely that the reported estimate will be greater than in practice. Threshold analyses have therefore been undertaken to investigate the impact of assuming lower sensitivity values in particular.<br><br>Threshold analyses assumed sensitivity estimates between 1 and 0·55.
Specificity of UCPCR test	Based on data from Besser et al[6] which used a prevalent case-control diagnostic study design: 0.96.	Since the specificity estimate for the UCPCR test is from a case-control diagnostic study, it is likely that the reported estimate will be greater than in practice. Threshold analyses have therefore been undertaken to investigate the impact of assuming lower specificity values in particular.<br><br>Threshold analyses assumed specificity estimates between 1 and 0·55.
Sensitivity of autoantibody test	Based on data from MacDonald et al[7] which used a prevalent case-control diagnostic study design: 0.99.	Since the sensitivity estimate for the autoantibody test is from a case-control diagnostic study, it is likely that the reported estimate will be greater than in practice. Threshold analyses have therefore been undertaken to investigate the impact of assuming lower sensitivity values in particular.<br><br>Threshold analyses assumed sensitivity estimates between 1 and 0·55.
Specificity of autoantibody test	Based on data from MacDonald et al[7] which used a prevalent case-control diagnostic study design: 0.82.	Since the specificity estimate for the autoantibody test is from a case-control diagnostic study, it is likely that the reported estimate will be greater than in practice. Threshold analyses have therefore been undertaken to investigate the impact of assuming different specificity values.<br><br>Threshold analyses assumed specificity estimates between 1 and 0·55.
Percentage of individuals with *GCK* mutation who are receiving insulin treatment at the	Based on data from the accompanying clinical study which investigated the application of the Biomarker Testing strategy. 75% of individuals with *GCK* mutation are	Threshold analyses assuming 100% to 10% (in 10% decrements) of individuals with *GCK* mutations are receiving insulin at the start of the model.

start of the model	receiving insulin treatment at the start of the model, while 25% are receiving tablets (metformin and sulphonylureas).	
Percentage of individuals with *HNF1A* or *HNF4A* mutation who are receiving insulin treatment at the start of the model	Based on data from the accompanying clinical study which investigated the application of the Biomarker Testing strategy. 67% of individuals with *HNF1A* or *HNF4A* mutation are receiving insulin treatment at the start of the model, 25% are receiving tablets (metformin and sulphonylureas) and 8% are not treated pharmacologically.	Threshold analyses assuming 100% to 10% (in 10% decrements) of individuals with *HNF1A* or *HNF4A* mutations are receiving insulin at the start of the model.
Percentage of individuals with *HNF1A* or *HNF4A* mutations who remain on most appropriate treatment after a diagnosis of monogenic diabetes	Based on data from the accompanying clinical study which investigated the application of the Biomarker Testing strategy. At every follow-up point after treatment change, 100% of individuals with *HNF1A* or *HNF4A* mutations remained on the most appropriate treatment.	The base case estimates are based on a small number of participants. Threshold analyses have been conducted to investigate the percentage of individuals with *HNF1A* or *HNF4A* mutations who need to remain on tablets for the strategies to be cost-saving compared to No Testing.<br><br>It was assume that for all follow-up time periods after a monogenic diabetes diagnosis, the percentage receiving tablets is: 86%, 77%, 50%, 25% or 10%.
Cascade family testing	Analysis of referral rate data[4] indicate that for every 10 case of monogenic diabetes identified, 6.2 family members are also genetically tested: with 5.6 being positive for monogenic diabetes and 0.6 being negative for monogenic diabetes.	The impact of family cascade testing in the Ad Hoc Testing, Clinical Prediction Model Testing and Biomarker Testing strategies was investigated by removing all cascade family testing from the strategies.<br><br>Estimates of the magnitude of cascade family testing based on the 95% confidence interval limits are used to investigate the impact of this parameter: 4.7 to 6.5 family members who are found to be positive for monogenic diabetes, and 0. 3 to 1 family members who are found to be negative for monogenic diabetes.
Frequency of HBGM before and after changing treatment due to a diagnosis of monogenic diabetes	Based on data from the accompanying clinical study which investigated the application of the Biomarker Testing strategy. Data suggested that individuals with *GCK* mutations stopped HBGM after their diagnosis of monogenic diabetes, while individuals with *HNF1A* or *HNF4A* mutations significantly reduced their frequency of HBGM after a diagnosis of monogenic diabetes.	The 95% confidence limits for the estimated frequency of HBGM at the start of the model and at follow-up after a treatment change for individuals with *HNF1A* or *HNF4A* mutations were used in sensitivity analyses. The change in frequency of HBGM before and after a diagnosis of monogenic diabetes was maximised (which would favour strategies to identify cases of monogenic diabetes) by assuming the upper 95% confidence limit at baseline and the lower 95% confidence limits at follow-up. Conversely, the change in frequency of HBGM was minimised (which would not be as favourable to strategies to identify cases of monogenic diabetes) by assuming the lower 95% confidence limit at baseline and the upper 95% confidence limit at follow-up.

Table 1I Summary of “base case” results

Strategy	Total undiscounted LYs	Total discounted QALYs	Total discounted costs[a]	Incremental costs vs No Testing strategy[a]	% who are genetically tested	
					With monogenic diabetes	Without monogenic diabetes
Clinical Prediction Model[b]	38·4	11·9	£54,000	-£100	93	3
Biomarker			£54,000	-£100	93	5
Ad Hoc			£54,100	0	7	<1
No Testing			£54,100	NA	0	0
All Testing			£54,400	£300	93	93

[a] rounded to nearest £100; [b] thresholds chosen to maximise costs saved

Fig 1A Incremental costs (vs No Testing) and the proportion of monogenic diabetes cases identified for each strategy

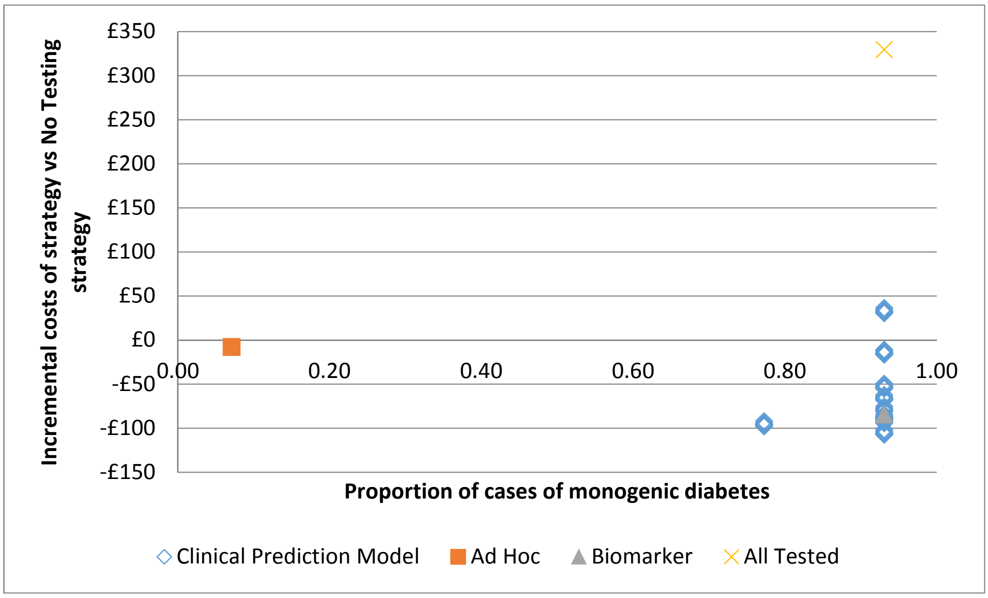

Peters JL, *et al. BMJ Open* 2020; 10:e034716. doi: 10.1136/bmjopen-2019-034716

Fig 1B Tornado plot of sensitivity analyses for the Ad Hoc Testing strategy

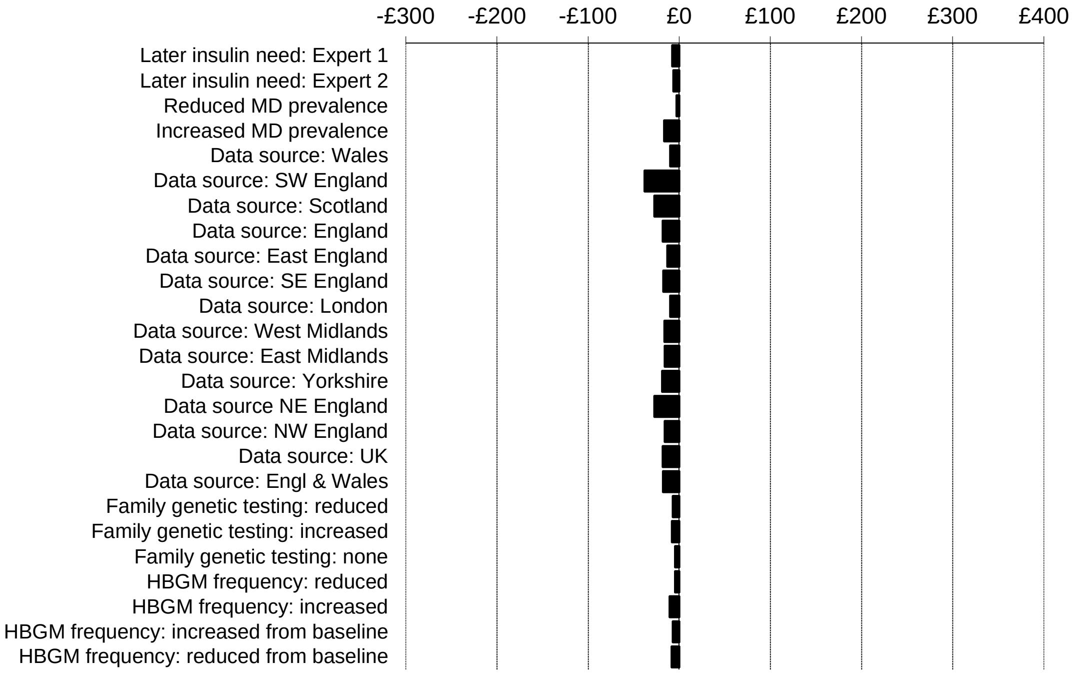

Fig 1C Tornado plot for the Clinical Prediction Model Testing strategy

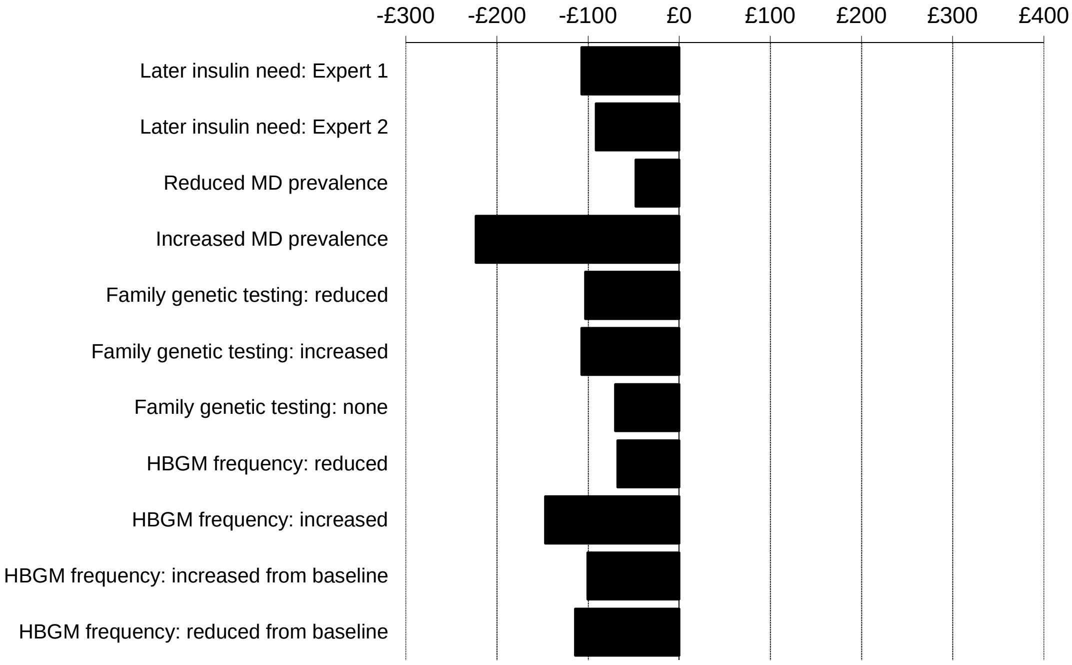

Peters JL, *et al. BMJ Open* 2020; 10:e034716. doi: 10.1136/bmjopen-2019-034716

Fig 1D Tornado plot for the Biomarker Testing strategy

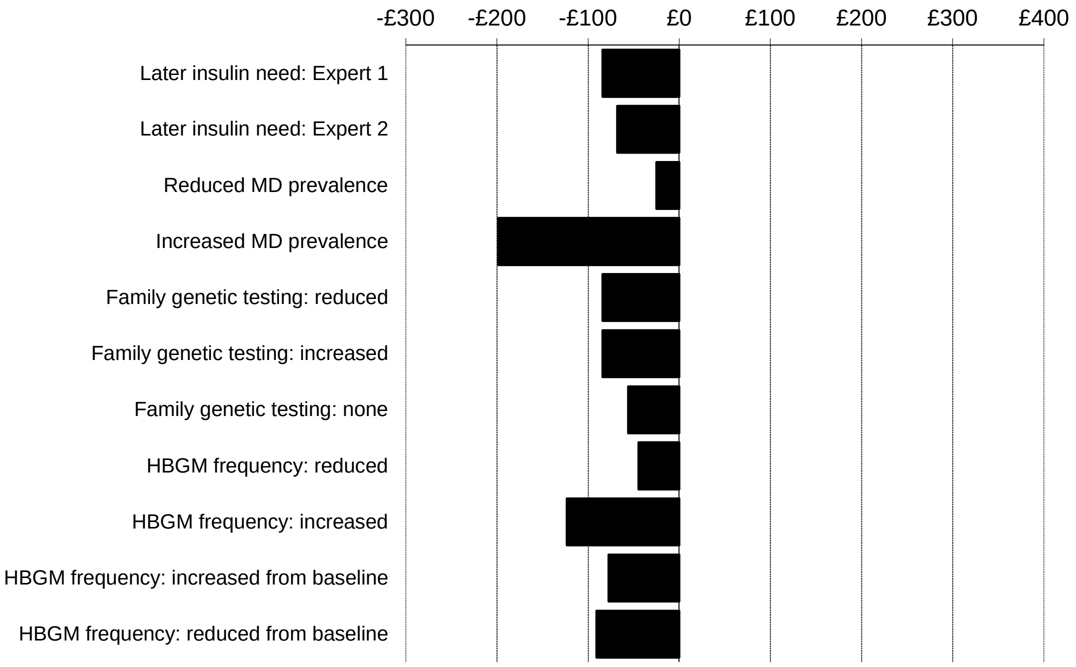

Fig 1E Tornado plot for the All Testing strategy

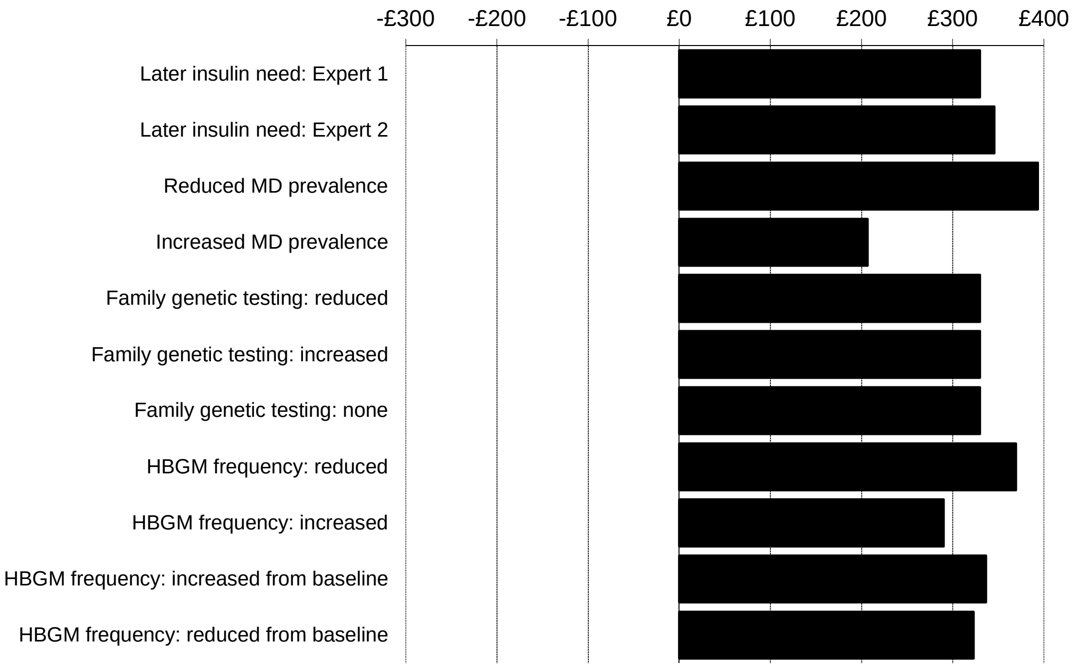

Peters JL, *et al. BMJ Open* 2020; 10:e034716. doi: 10.1136/bmjopen-2019-034716

Fig 1F Incremental costs (vs No Testing) for all strategies for reducing percentage of *GCK* cohort starting on insulin

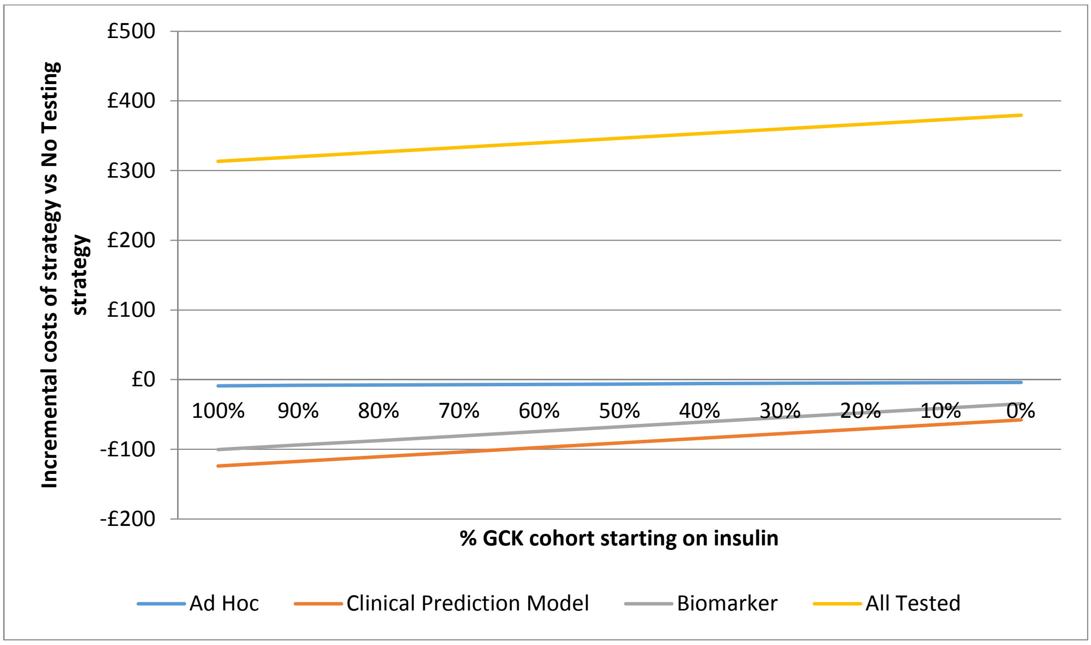

Fig 1G Incremental costs (vs No Testing) for all strategies for reducing percentage of *HNF1A* and *HNF4A* cohort starting on insulin

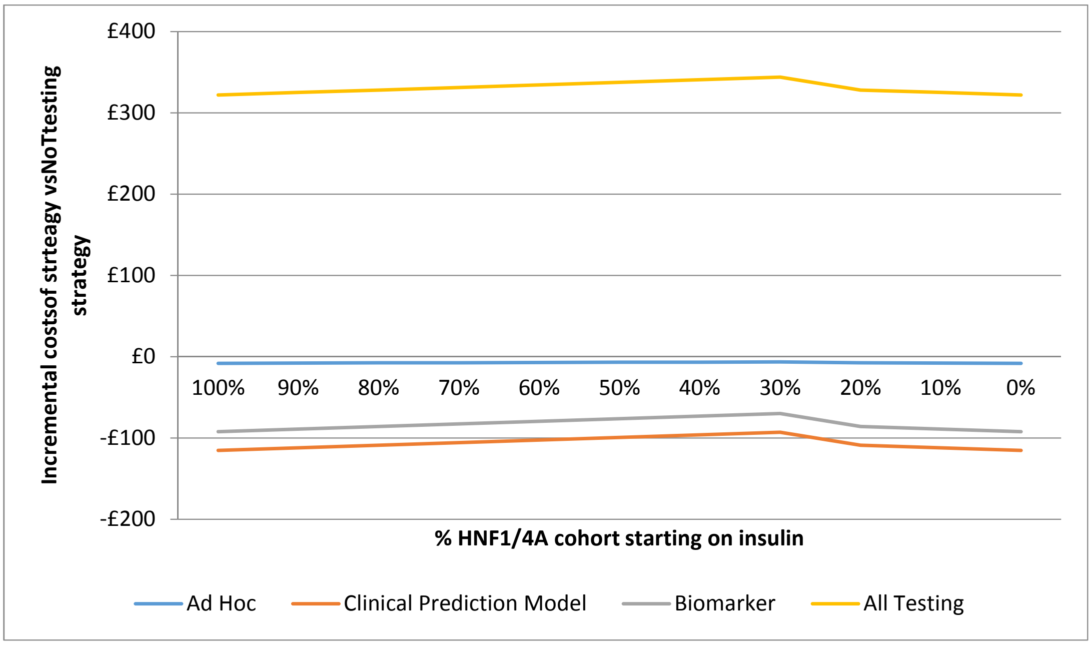

Peters JL, *et al. BMJ Open* 2020; 10:e034716. doi: 10.1136/bmjopen-2019-034716

Fig 1H Incremental costs (vs No Testing) for the Biomarker Testing strategy with reducing levels of UCPCR and antibody testing uptake

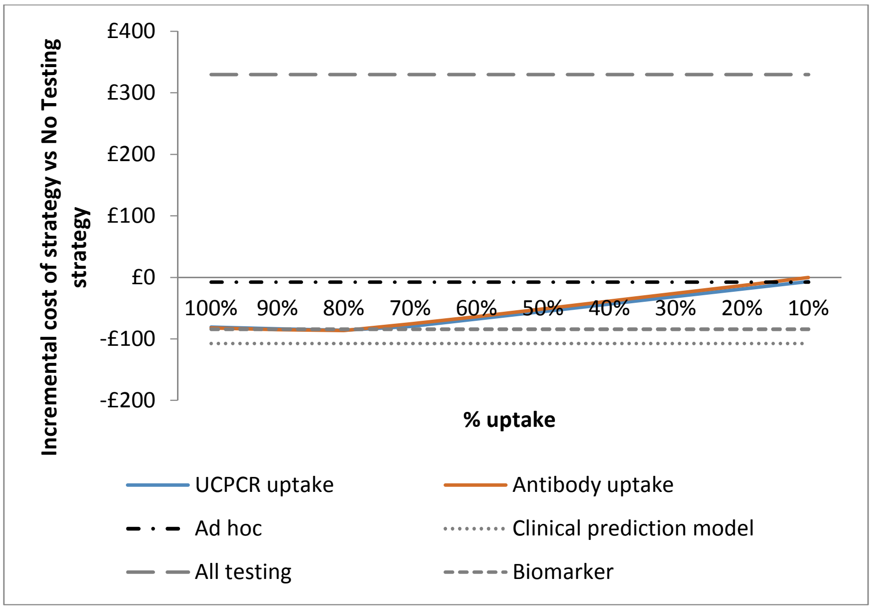

Fig 1I Incremental costs (vs No Testing) for the Biomarker Testing strategy with reducing estimates of sensitivity and specificity for the UCOCR and antibody tests

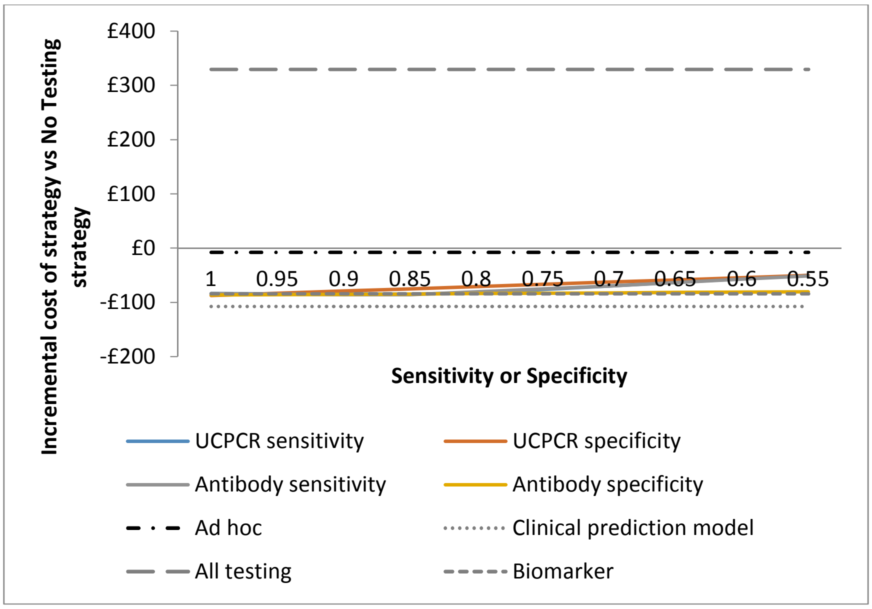

Peters JL, *et al. BMJ Open* 2020; 10:e034716. doi: 10.1136/bmjopen-2019-034716

Fig 1J Incremental costs for the Biomarker Testing strategy (vs No Testing) with increasing estimates of repeat samples and UCPCR and autoantibody tests

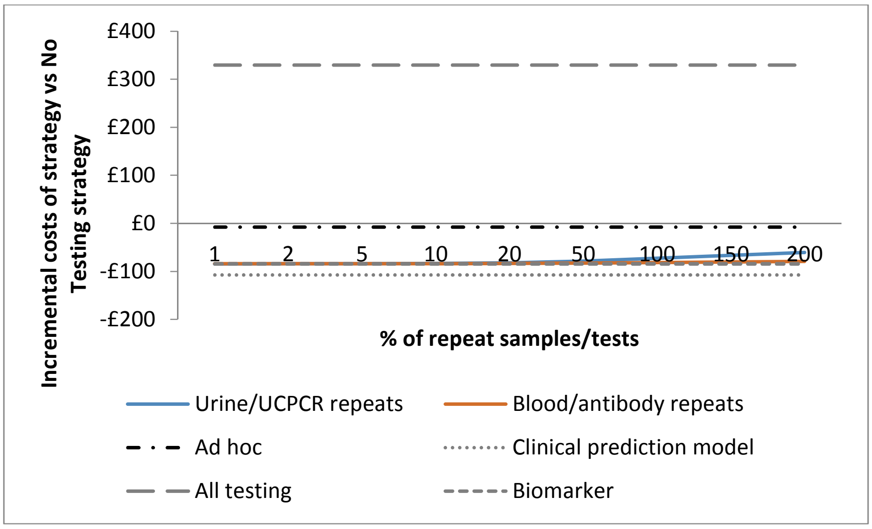

Fig 1K Incremental costs (vs No Testing) for all strategies when genetic test costs are reduced

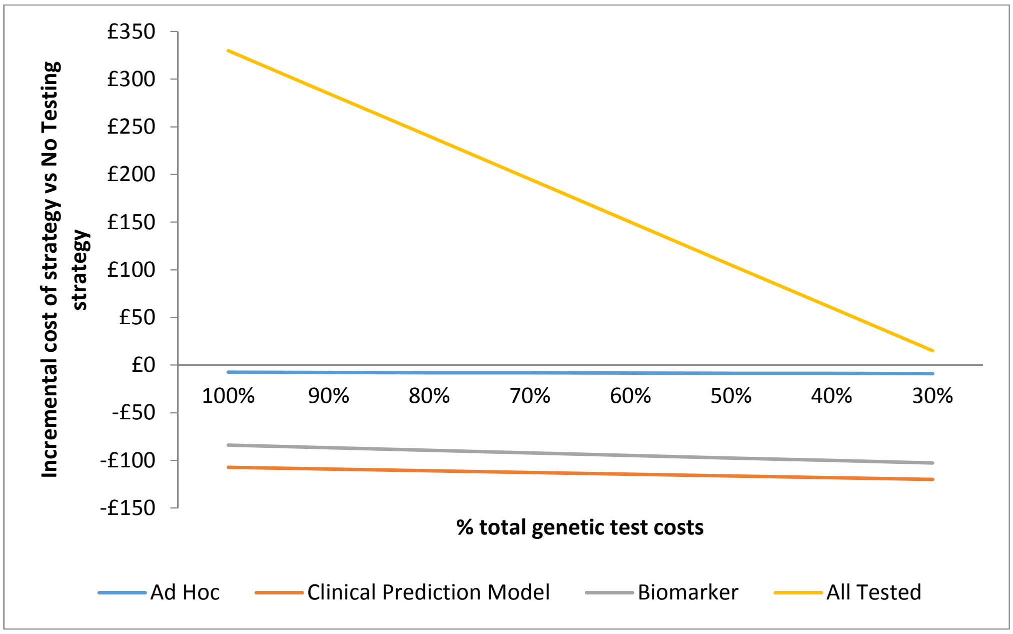

Peters JL, *et al. BMJ Open* 2020; 10:e034716. doi: 10.1136/bmjopen-2019-034716

References

1. Shields BM, Shepherd M, Hudson M, et al. Population-Based Assessment of a Biomarker-Based Screening Pathway to Aid Diagnosis of Monogenic Diabetes in Young-Onset Patients. *Diabetes Care* 2017;40(8):1017-25. doi: 10.2337/dc17-0224 [published Online First: 2017/07/14]
2. Llaurado G, Gonzalez-Clemente J-M, Maymo-Masip E, et al. Serum levels of TWEAK and scavenger receptor CD163 in type 1 diabetes mellitus: relationship with cardiovascular risk factors. A case-control study. *PLoS One* 2012;7(8):e43919.
3. Office for National Statistics. Population Estimates by Ethnic Group 2002-2009. Statistical Bulletin, 2011.
4. Shields BM, Hicks S, Shepherd MH, et al. Maturity-onset diabetes of the young (MODY): how many cases are we missing? *Diabetologia* 2010;53:2504-08.
5. Royal Devon and Exeter NHS Foundation Trust. [Available from: http://www.rdehospital.nhs.uk/prof/molecular_genetics/tests/Full_Test_List.htm accessed 13th October 2014.
6. Besser REJ, Shepherd MH, McDonald TJ, et al. Urinary c-peptide-to-creatinine ratio is a practical outpatient tool for identifying hepatocyte nuclear factor 1-α/hepatocyte nuclear factor 4-α maturity-onset diabetes of the young from long-duration type 1 diabetes. *Diabetes Care* 2011;34:1-6.
7. McDonald TJ, Colclough K, Brown R, et al. Islet autoantibodies can discriminate maturity-onset diabetes of the young (MODY) from type 1 diabetes. *Diabetic Medicine* 2011;28:1028-33.
