# Supplementary material for: Strategies to identify individuals with monogenic diabetes: results of an economic evaluation
Source: BMJ Open. 2020 Mar 18;10(3):e034716. doi: 10.1136/bmjopen-2019-034716 (PMC7150598; doi:10.1136/bmjopen-2019-034716)
Supplement: Supplementary data [file bmjopen-2019-034716supp002.pdf]

Supplementary Data 2: Parameters and results for Cohort 1

Cohort 1 - Diagnosed with diabetes <30yrs old and still <50 yrs old at start of model

Table 2A Characteristics of the modelled cohorts 1 and 2 at entry to the model

Characteristic	Parameter value	Evidence source
Prevalence (95% confidence interval)		
GCK mutation	0·7% (0.4%, 1.4%)	Shields et al[1] & unpublished data from accompanying clinical study (N=1407)
HNF1A mutation	1·5% (1.2%, 2.7%)	Shields et al[1] & unpublished data from accompanying clinical study (N=1407)
HNF4A mutation	0·2% (0.1%, 0.6%)	Shields et al[1] & unpublished data from accompanying clinical study (N=1407)
Type 1 diabetes[a]	88·6% (86.4%, 89.9%)	Unpublished data from accompanying clinical study (N=1407)
Type 2 diabetes	9.0% (7.4%, 10.5%)	Unpublished data from accompanying clinical study (N=1407)
Age (years) [b]	25	Unpublished data from accompanying clinical study (N=1407)
Time since diagnosis (years) [b]	12	
Body mass index [b]	24·4	
HbA1c (mmol/mol) [b]	64.2	
Female (%)	50	
Systolic blood pressure [b]	131·7	[2]
Total cholesterol [b]	4·74	[2]
High density lipoprotein [b]	1·31	[2]
Low density lipoprotein [b]	2·61	[2]
Triglycerides [b]	0·83	[2]
Caucasian	89%	[3]

Black	4%	[3]
Asian	7%	[3]

[a] Defined as receiving insulin treatment within 12 months of diabetes diagnosis.
[b]Mean.

Table 2B Percentage (95% CI) of referred individuals tested for mutations in *GCK* and/or *HNF1A* and *HNF4A* genes by true diagnosis (from unpublished UK referral centre data)

True diabetes diagnosis	Percentage (95% CI) [N=2294]		
	***GCK* only**	***HNF1A* and *HNF4A***	***GCK*, *HNF1A* and *HNF4A***
Not monogenic	14.1% (12.3%, 16.0%)	70.0% (67.5%, 72.4%)	15.9% (14.0%, 18.0%)
GCK mutation	95.2% (92.3%, 97.3%)		4.8% (2.7%, 7.7%)
HNF1A mutation		96.2% (94.0%, 97.8%)	3.5% (2.0%, 5.7%)
HNF4A mutation		97.3% (93.2%, 99.2%)	2.7% (0.7%, 6.8%)

Table 2C Percentage (95% CI) of cohort not accepting offer of testing, or requiring multiple tests for the Biomarker Testing strategy

	Cohort 1	
Number of tests	UCPCR (including urine sample) N=2017	Autoantibody (including blood sample) N=624
0	11·9% (10.6%, 13.4%)	8·2% (6.1%, 10.6%)
1	86·1% (84.5%, 87.6%)	90·0% (87.4%, 92.3%)
2	1·8% (1.3%, 2.5%)	1·8% (0.9%, 3.1%)
3	0·1% (0.03%, 0.4%)	0%

UCPCR, urinary c-peptide creatinine ratio. Unpublished data from accompanying clinical study.

Table 2D Multipliers (and 95% confidence intervals) to inform cascade genetic testing of diabetic family members

**Number of relatives test per true monogenic diabetes case identified**	Multipliers (and 95% CIs)	**Data source**
Relatives positive for monogenic diabetes	5.9 (5.4, 6.3)	Re-analysis of Shields et al[4] (specific to definition of modelled cohort)
Relatives negative for monogenic diabetes	0.4 (0.2, 0.6)	

Table 2E Pre-genetic test treatment pattern, cost and frequency of HBGM by true diagnosis

**Diabetes type**	**Treatment**	**% receiving treatment**	**Mean monthly treatment costs**	**Mean frequency of HBGM[a]**
Type 1	Insulin only	100%	£52	78
Type 2	Insulin only	36%	£55	43
	Insulin + tablets	54%	£50	43
	Tablets only	3%	£2	17
	No diabetes treatment	7%	£0	0
GCK mutation	Insulin only	87.5% (47.3%, 99.7%)	£10	63 (19, 107)
	Tablets only	12.5% (0.3%, 52.6%)	£1	0
HNF1A and *HNF4A* mutation	Insulin only	78.4% (61.8%, 90.2%)	£23	
	Insulin + tablets	13.5% (4.5%, 28.8%)	£16	76 (52, 99)
	Tablets	5.4% (0.1%, 18.2%)	£2	
	No diabetes treatment	2.7% (0.1%, 14.2%)	£0	0

[a] HBGM, home blood glucose monitoring

Table 2F Estimated dose and timing of future insulin requirements for individuals identified as having *HNF1A* or *HNF4A* mutations

**Population**	**Expert 1**		**Expert 2**	
	**Years after start of model**	**Insulin need (u)**	**Years after start of model**	**Insulin need (U/kg)**
Tablets only	0-19<br>20-24<br>25-29<br>≥30 yrs	As at model start<br>10 + tablets<br>20+ tablets<br>30 + tablets	0-9<br>10-14<br>15-24<br>≥2 yrs	As at model start<br>0.25 + tablets<br>0.4 + tablets<br>0.5 (no tablets)
Tablets and insulin	0-4<br>5-14<br>≥15 yrs	As at model start<br>20 + tablets<br>30 + tablets	0-9<br>10-14<br>≥15 yrs	As at start of model<br>0.4 + tablets<br>0.5 (no tablets)
Insulin only	0-9<br>10-24<br>≥25 yrs	As at model start<br>50<br>60	≥0 yrs	0.5

Table 2G Post-diagnosis HBGM frequency (95%CI) by treatment changed to and true diagnosis

	**Time since diagnosis of monogenic diabetes (months)**			
**Mutation - Treatment received**	**1**	**3 months**	**6 months**	**12 months**
GCK mutation – no diabetes treatment	0	0	0	0
HNF1/4A mutation – tablets only	50 (27, 73)	36 (14, 57)	22 (11, 33)	21 (10, 32)
HNF1/4A mutation – insulin and tablets	89 (56, 121)	66 (44, 87)	70 (46, 93)	43 (25, 60)

Table 2H Justification of parameter values and variations used in base case and sensitivity analyses

Parameter	Base case justification	Justification of sensitivity/threshold analyses
Prevalence of monogenic diabetes	In the accompanying clinical study, the total number of cases of monogenic diabetes was 34 from a total of 1407 individuals screened. This leads to an estimated prevalence within the definition of Cohort 1 of 34/1407 = 2·4%.	Although the total screened population was 1407 in the accompanying clinical study[1], the total eligible population in the defined geographical area was 2288. We could therefore assume:<br>1. that no more cases would have been found in the remaining eligible population not screened, i.e. the remaining 881 were not screened as they were quite obviously ***not*** cases of monogenic diabetes, therefore a lower estimate of the prevalence of monogenic diabetes might be appropriate (34/2288 = 1·5%),<br>2. there were no differences between those not screened and those who were screened, and so the base case numbers would not change (34/1407 = 2·4%)<br>3. those 881 who did not complete screening were ***more*** likely to be cases of monogenic diabetes. As an upper estimate, we assume the prevalence of monogenic diabetes in the defined cohort is doubled (68/1407 = 4·8%).<br>To investigate an increase or decrease in the prevalence of monogenic diabetes, sensitivity analyses assumed scenarios 1 and 3 above.
Sensitivity and specificity of the Ad Hoc Testing strategy	Based on referral rate data for Northern Ireland (the region with the lowest referral rates)[4]	Sensitivity analyses were based on all regions analysed by Shields et al[4]
Sensitivity of UCPCR test	Based on data from Besser et al[5] which used a prevalent case-control diagnostic study design: 0.94.	Since the sensitivity estimate for the UCPCR test is from a case-control diagnostic study, it is likely that the reported estimate will be greater than in practice.<br><br>Threshold analyses assumed sensitivity estimates for the UCPCR test between 1 and 0·55 (in 0.05 decrements). Results assuming a sensitivity of 1 or 0.55 are presented.
Specificity of UCPCR test	Based on data from Besser et al[5] which used a prevalent case-control diagnostic study design: 0.96.	Since the specificity estimate for the UCPCR test is from a case-control diagnostic study, it is likely that the reported estimate will be greater than in practice.<br><br>Threshold analyses assumed specificity estimates for the UCPCR test between 1 and 0·55 (in 0.05 decrements). Results assuming a specificity of 1 or 0.55 are shown.
Sensitivity of autoantibody test	Based on data from MacDonald et al[6] which used a prevalent case-control diagnostic study design: 0.99.	Since the sensitivity estimate for the autoantibody test is from a case-control diagnostic study, it is likely that the reported estimate will be greater than in practice.<br><br>Threshold analyses assumed sensitivity estimates for the autoantibody test between 1 and 0·55 (in 0.05 decrements).<br>Results assuming a sensitivity of 1 or 0.55 are shown.

Specificity of autoantibody test	Based on data from MacDonald et al[6] which used a prevalent case-control diagnostic study design: 0.82.	Since the specificity estimate for the autoantibody test is from a case-control diagnostic study, it is likely that the reported estimate will be greater than in practice.<br><br>Threshold analyses assumed specificity estimates for the autoantibody test between 1 and 0·55 (in 0.05 decrements).<br>Results assuming a sensitivity of 1 or 0.55 are shown.
Uptake of UCPCR test	Based on data from the accompanying clinical study which investigated the application of the Biomarker strategy.<br>Uptake of UCPCR was assumed to be 88%.	Threshold analyses where UCPCR test uptake was assumed to range from 100% to just 10% (in 10% decrements).<br>It was hypothesised that test uptake in practice is likely to be lower than test uptake in the accompanying clinical study where individuals have consented to participating in a study.<br><br>Results of assumptions that uptake of UCPCR is 100% or 10% are reported.
Uptake of autoantibody test	Based on data from the accompanying clinical study which investigated the application of the Biomarker strategy.<br>Uptake of autoantibody testing was assumed to be 92%.	Threshold analyses where autoantibody test uptake was assumed to range from 100% to just 10% (in 10% decrements).<br><br>It was hypothesised that test uptake in practice is likely to be lower than test uptake in the accompanying clinical study where individuals have consented to participating in a study.<br><br>Results of assumptions that uptake of autoantibody testing is 100% or 10% are reported.
Uptake of genetic test	Based on data from the accompanying clinical study which investigated the application of the Biomarker strategy.<br>Uptake of genetic testing was assumed to be the same as for autoantibody testing (92%) since the same blood sample for autoantibody testing was used for the genetic testing.	Threshold analyses where genetic test uptake was assumed to range from 100% to just 10% (in 10% decrements).<br><br>It was hypothesised that test uptake in practice is likely to be lower than test uptake in the accompanying clinical study where individuals have consented to participating in a study.<br><br>Results of assumptions that uptake of genetic testing is 100% or 10% are reported.
Repeat urine samples and UCPCR tests	Based on data from the accompanying clinical study which investigated the application of the Biomarker strategy. The percentage of repeat urine samples and UCPCR tests was assumed to be 2%.	Threshold analyses were undertaken assuming no repeats, 1%, 5%, 10%, 20%, 50%, 100%, 150% and 200% of samples and tests needed to be repeated. 200% repeat samples and tests can be interpreted as every individual requiring another 2 urine samples and UCPCR tests to be done, so that in total every individual has provided 3 urine samples and 3 UCPCR tests have been done – an extreme assumption.<br><br>Results for assuming 200% repeat samples and tests are presented.
Repeat blood samples and	Based on data from the accompanying clinical study which investigated the	Threshold analyses were undertaken assuming no repeats, 1%, 5%, 10%, 20%, 50%, 100%, 150% and 200% of samples and tests needed to be repeated. 200%

autoantibody tests	application of the Biomarker strategy. The percentage of repeat blood samples and autoantibody tests was assumed to be 2%.	repeat samples and tests can be interpreted as every individual requiring another 2 blood samples and autoantibody tests to be done, so that in total every individual has provided 3 blood samples and 3 autoantibody tests have been done, clearly an extreme assumption.<br><br>Results for assuming 200% repeat samples and tests are presented.
Percentage of individuals with *GCK* mutation who are receiving insulin treatment at the start of the model	Based on data from the accompanying clinical study which investigated the application of the Biomarker strategy. 88% of individuals with *GCK* mutation are receiving insulin treatment at the start of the model, while 12% are receiving tablets (metformin and sulphonylureas).	Threshold analyses assuming 100% to 10% (in 10% decrements) of individuals with *GCK* mutations are receiving insulin at the start of the model.<br><br>Results from assuming 100% or 10% are receiving insulin at the start of the model are presented.
Percentage of individuals with *HNF1A* or *HNF4A* mutation who are receiving insulin treatment at the start of the model	Based on data from the accompanying clinical study which investigated the application of the Biomarker strategy. 78% of individuals with *HNF1A* or *HNF4A* mutation are receiving insulin treatment at the start of the model, 5% are receiving insulin and tablets (metformin and sulphonylureas), 14% are receiving tablets and 3% are not treated pharmacologically.	Threshold analyses assuming 100% to 10% (in 10% decrements) of individuals with *HNF1A* or *HNF4A* mutations are receiving insulin at the start of the model.<br><br>Results from assuming 100% or 10% are receiving insulin at the start of the model are presented.
Genetic test cost	UK referral centre costs[7]: £350 for *GCK* mutation; £450 for *HNF1A* and *HNF4A* mutations.	Threshold analyses were conducted to identify at what cost of genetic tests would the All Tested strategy incur no additional costs over the No Testing strategy. Costs of tests for GCK and HNF1A and HNF4A mutations were reduced in 10% steps to just 10% of their base case costs: £35 for GCK and £45 for HNF1A and HNF4A.<br><br>Results of assumptions that genetic costs are 100% or 10% of their current costs are reported.
Long-term insulin need for individuals with *HNF1A* or *HNF4A* mutations	Expert 1	Expert 2, who assumed a larger dose of insulin would generally be required sooner than that stated by Expert 1.
Percentage of individuals with *HNF1A* or *HNF4A* mutations who remain on most appropriate treatment after a diagnosis of	Based on data from the accompanying clinical study which investigated the application of the Biomarker strategy. At 1 and 3 months after changing to more appropriate treatment, 86% are receiving tablets only (sulphonylureas and	The base case estimates are based on a small number of participants. Threshold analyses have been conducted to investigate the percentage of individuals with *HNF1A* or *HNF4A* mutations who need to remain on tablets for the strategies to be cost-saving compared to No Testing.

monogenic diabetes	metformin). At 6 and 12 months 89% and 77% are on tablets only, respectively.	It was assume that for all follow-up time periods after a monogenic diabetes diagnosis, the percentage receiving tablets is: 100%, 50%, 25% or 10%.<br><br>Results assuming 100% and 10% receive tablets are presented.
Cascade family testing	Analysis of referral rate data[7] indicate that for every 10 case of monogenic diabetes identified, 6.3 family members are also genetically tested: with 5.9 being positive for monogenic diabetes and 0.4 being negative for monogenic diabetes.	The impact of family cascade testing in the Ad Hoc, Clinical Prediction Model and Biomarker strategies was investigated by removing all cascade family testing from the strategies.<br><br>Estimates of the magnitude of cascade family testing based on the upper 95% confidence interval limits are used where 6.3 family members are found to be positive for monogenic diabetes, and 0.6 are found to be negative for monogenic diabetes, compared to the scenario where there is no family testing.
Frequency of HBGM before and after changing treatment due to a diagnosis of monogenic diabetes	Based on data from the accompanying clinical study which investigated the application of the Biomarker strategy. Data suggested that individuals with *GCK* mutations stopped HBGM after their diagnosis of monogenic diabetes, while individuals with *HNF1A* or *HNF4A* mutations significantly reduced their frequency of HBGM after a diagnosis of monogenic diabetes.	The 95% confidence limits for the estimated frequency of HBGM at the start of the model and at follow-up after a treatment change for individuals with *HNF1A* or *HNF4A* mutations were used in sensitivity analyses. The change in frequency of HBGM before and after a diagnosis of monogenic diabetes was maximised (which would favour strategies to identify cases of monogenic diabetes) by assuming the upper 95% confidence limit at baseline and the lower 95% confidence limits at follow-up. Conversely, the change in frequency of HBGM was minimised (which would not be as favourable to strategies to identify cases of monogenic diabetes) by assuming the lower 95% confidence limit at baseline and the upper 95% confidence limit at follow-up.

UCPCR, urinary c-peptide to creatinine ratio; HBGM, home blood glucose monitoring

Fig 2A Incremental costs (vs No Testing) for all strategies for reducing percentage of *GCK* cohort starting on insulin

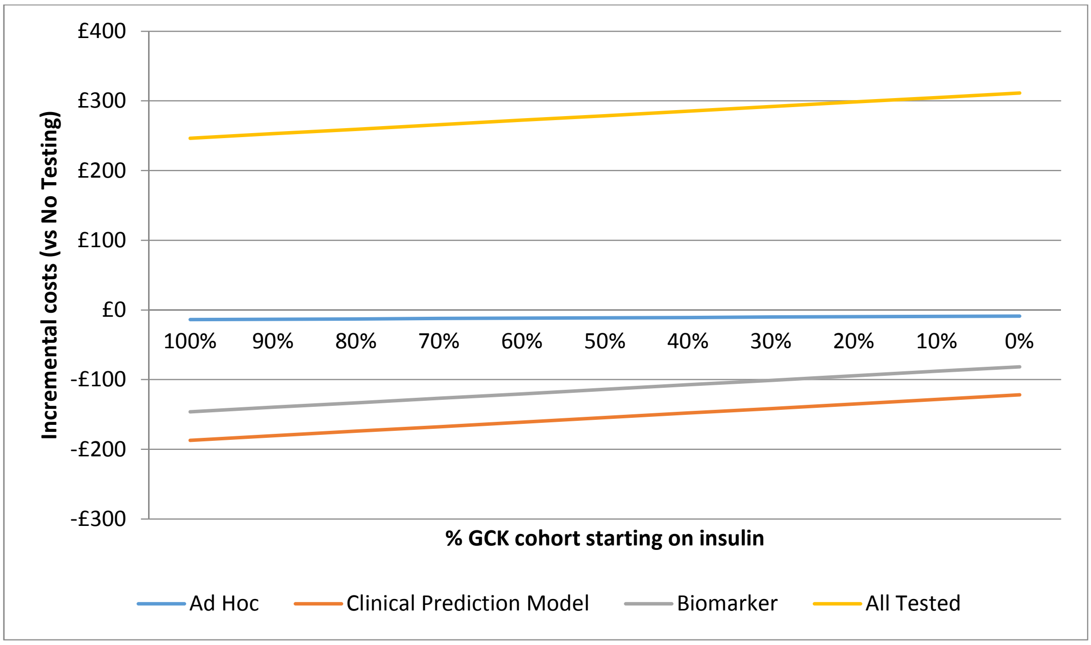

Fig 2B Incremental costs (vs No Testing) for all strategies for reducing percentage of *HNF1A* and *HNF4A* cohort starting on insulin

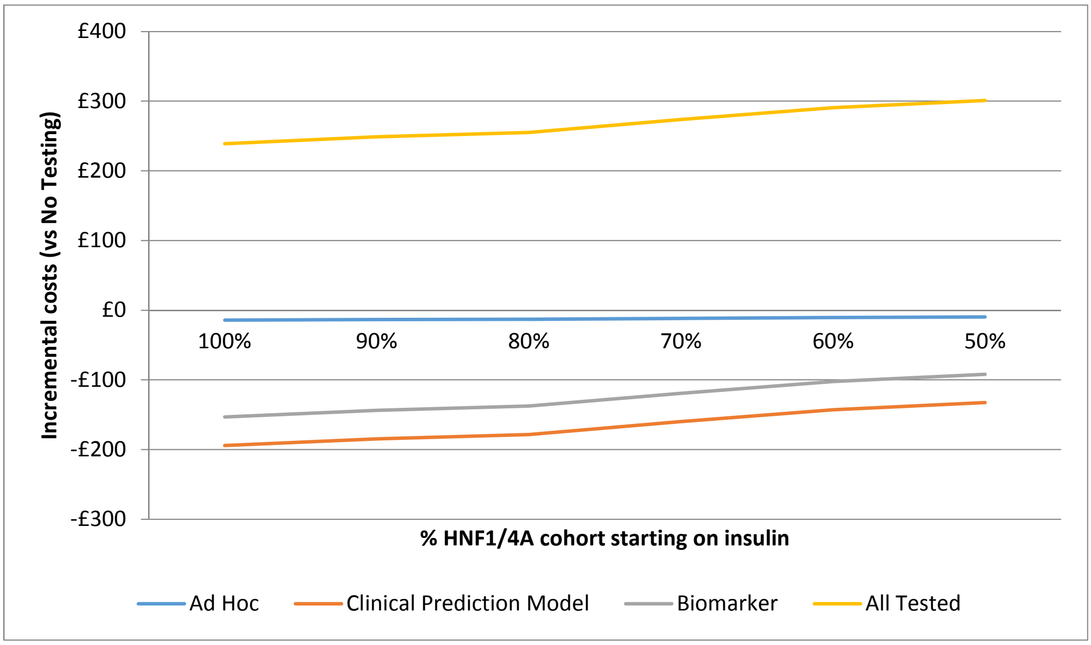

Fig 2C Incremental costs (vs No Testing) for the Biomarker Testing strategy with reducing levels of UCPCR and antibody testing uptake

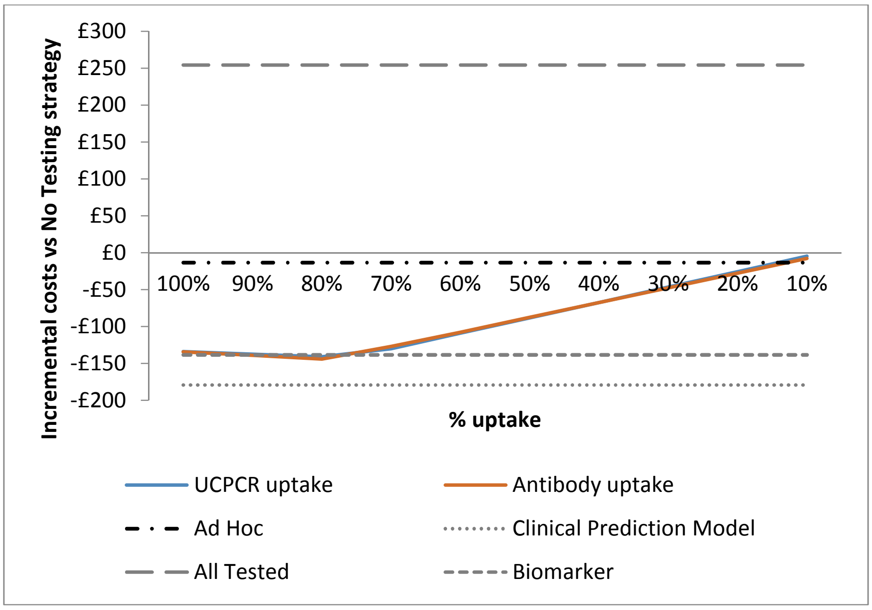

Fig 2D Incremental costs (vs No Testing) for the Biomarker Testing strategy with reducing estimates of sensitivity and specificity for the UCPCR and antibody tests

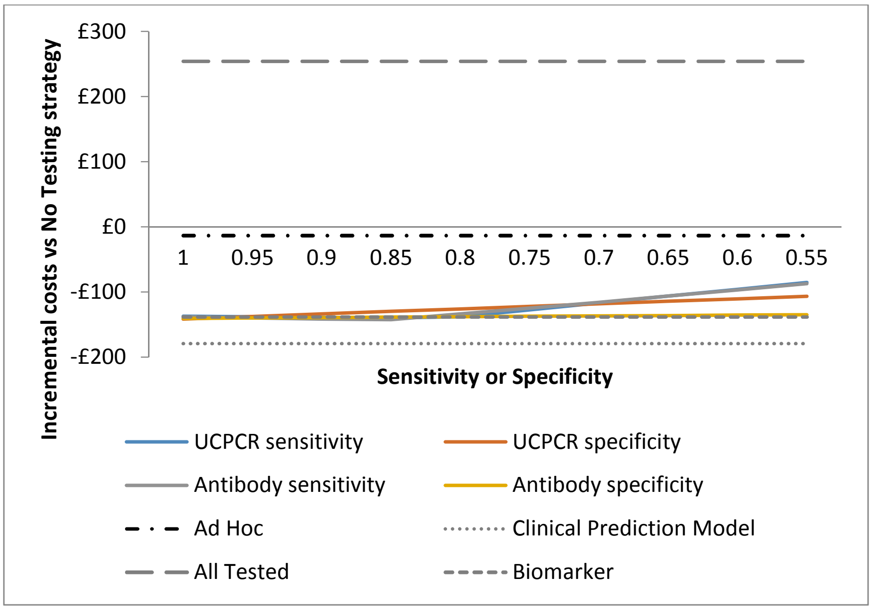

Fig 2E Incremental costs for the Biomarker Testing strategy (vs No Testing) with increasing estimates of repeat samples and UCPCR and autoantibody tests

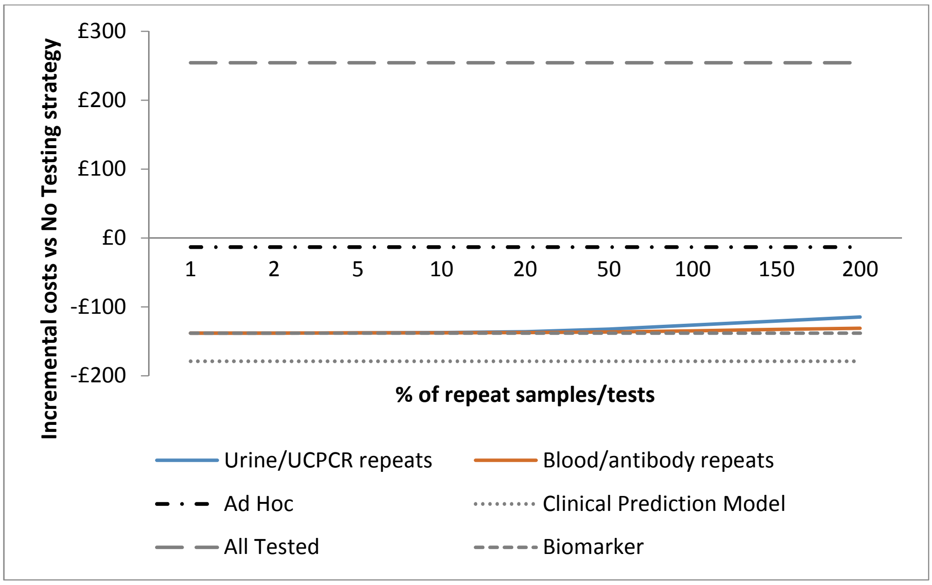

Fig 2F Incremental costs (vs No Testing) for all strategies when genetic test costs are reduced

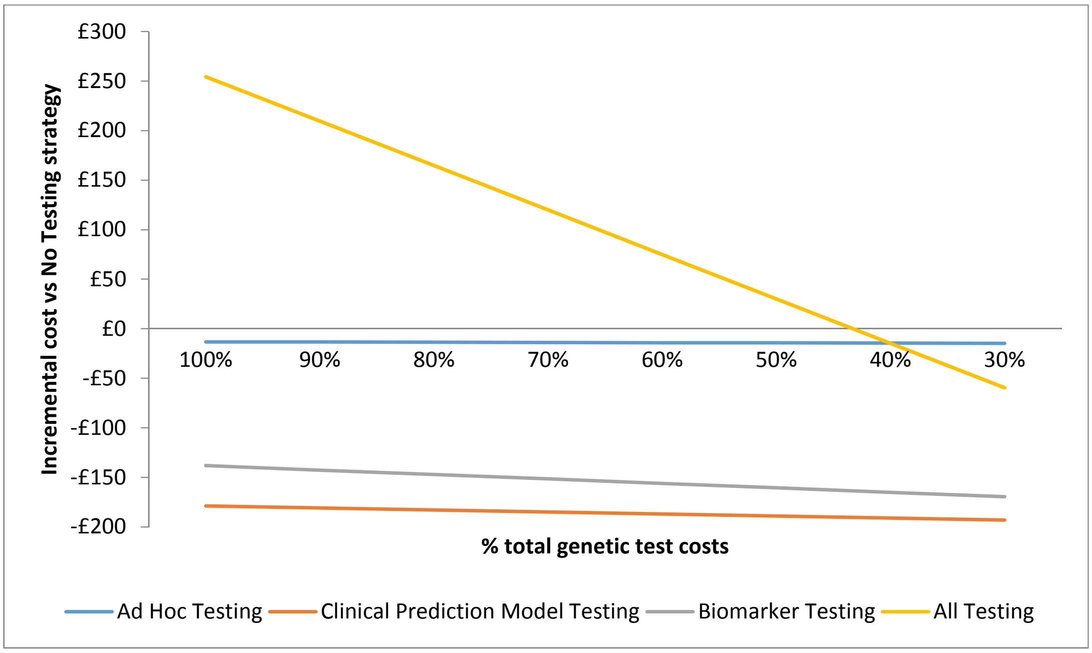

Utility improvement sensitivity analysis

In this sensitivity analysis it was assumed that individuals with HNF1A and HNF4A mutations who successfully transferred to sulphonylureas experienced an improvement in utility of 0.02 from one year after changing treatment (based on data from the associated clinical study). Please note that these analyses were run on an updated version of CORE (v9.0 rather than v8.5, as v8.5 no longer available). The total costs and QALYs are different, but importantly the incremental costs are the same as the results from v8.5.

Table 2I. Results of assuming improved utility for those successfully changing to sulphonylureas

Strategy	Total undiscounted costs [a]	Total discounted costs [a]	Incremental costs vs No Testing strategy [a]	Total discounted QALYs	Incremental QALYs vs No Testing strategy	% who are genetically tested		ICER vs No Testing [a]
						With monogenic diabetes	Without monogenic diabetes	
Clinical Prediction Model Testing[b]	£133,200	£65,900	-£100	10.3865	0.0013	92	3	-£111,700
Biomarker Testing	£133,300	£65,900	-£100	10.3865	0.0013	92	8	-£80,500
Ad Hoc Testing	£133,500	£66,000	0	10.3853	<0.001	6	<1	-£103,400
No Testing	£133,600	£66,000	NA	10.3852	NA	0	0	NA
All Testing	£133,700	£66,300	£300	10.3865	0.0013	92	92	£225,700

[a] rounded to nearest £100.

The total discounted QALYs for the Clinical Prediction Model, Biomarker and All Testing strategies are all the same (10.3865). This is because a maximum proportion of individuals with MODY are assumed to accept testing (92%), which is the case for these three strategies. The assumed proportion of individuals with HNF1A or HNF4A mutations who successfully change treatment (100%) does not depend on the testing strategy used. Thus, there is no difference in the proportion of people with HNF1A and HNF4A mutations who successfully change treatment between these three strategies, and so the total QALYs are the same. It is the relative costs of the strategies which allows some distinction between the Clinical Prediction Model, Biomarker and All Testing strategies.

For instance, the results suggest that the All Testing strategy would not be considered cost-effective by NICE willingness to pay per QALY gained thresholds (of £20,000 to £30,000). This is because it is estimated to cost £300 more, and produce a utility incremental of 0.0013 over the No Testing strategy, giving an ICER of £225,700.

As the ICERs for the Ad Hoc, Clinical Prediction Model and Biomarker Testing strategies are all estimated to cost less but produce more QALYs than the No Testing strategy (Fig X), there are all considered to be cost-effective options.

In a fully incremental analysis, the Clinical Prediction Model is considered to be the most cost-effective strategy – it produces the most QALYs at the least cost.

Fig 2G Cost-effectiveness plane for the sensitivity analysis which assumes an improvement in utility of 0.02 for those with HNF1A and HNF4A who successfully change treatment

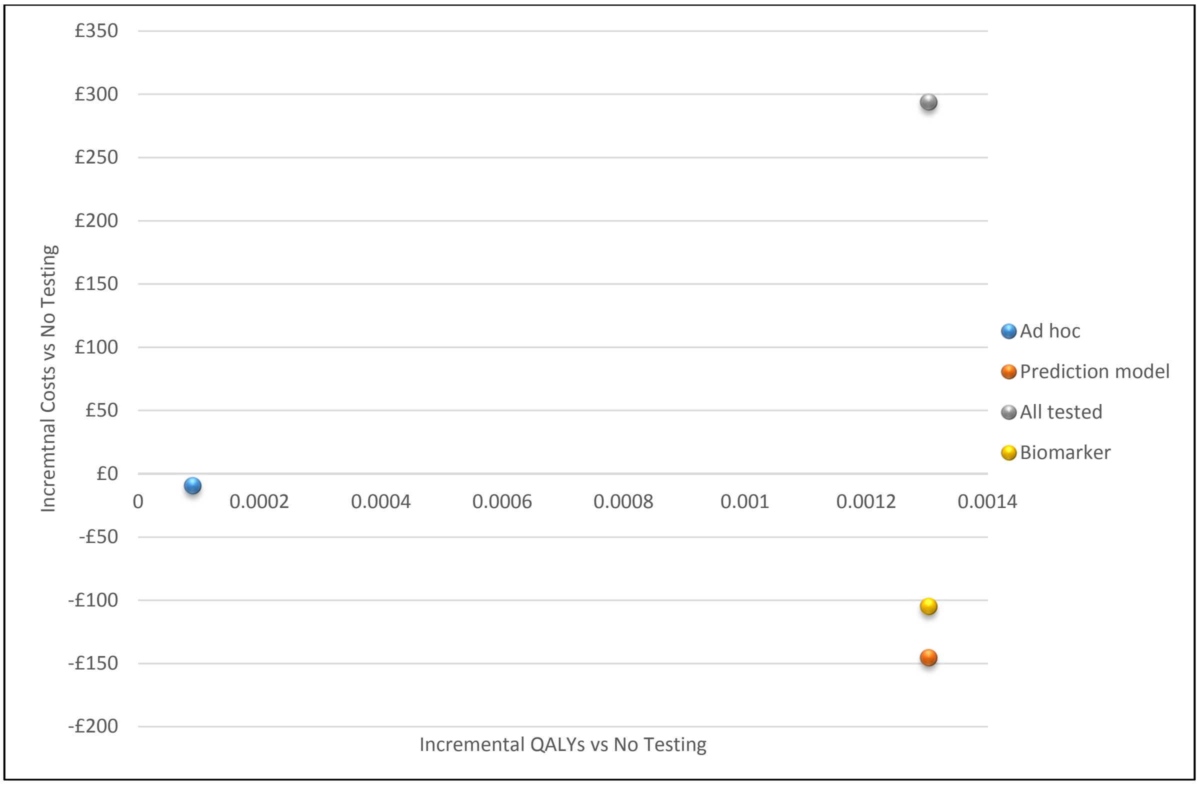

References

1. Shields BM, Shepherd M, Hudson M, et al. Population-Based Assessment of a Biomarker-Based Screening Pathway to Aid Diagnosis of Monogenic Diabetes in Young-Onset Patients. *Diabetes Care* 2017;40(8):1017-25. doi: 10.2337/dc17-0224 [published Online First: 2017/07/14]
2. Llaurado G, Gonzalez-Clemente J-M, Maymo-Masip E, et al. Serum levels of TWEAK and scavenger receptor CD163 in type 1 diabetes mellitus: relationship with cardiovascular risk factors. A case-control study. *PLoS One* 2012;7(8):e43919.
3. Office for National Statistics. Population Estimates by Ethnic Group 2002-2009. Statistical Bulletin, 2011.
4. Shields BM, Hicks S, Shepherd MH, et al. Maturity-onset diabetes of the young (MODY): how many cases are we missing? *Diabetologia* 2010;53:2504-08.
5. Besser REJ, Shepherd MH, McDonald TJ, et al. Urinary c-peptide-to-creatinine ratio is a practical outpatient tool for identifying hepatocyte nuclear factor 1-α/hepatocyte nuclear factor 4-α maturity-onset diabetes of the young from long-duration type 1 diabetes. *Diabetes Care* 2011;34:1-6.
6. McDonald TJ, Colclough K, Brown R, et al. Islet autoantibodies can discriminate maturity-onset diabetes of the young (MODY) from type 1 diabetes. *Diabetic Medicine* 2011;28:1028-33.
7. Royal Devon and Exeter NHS Foundation Trust. [Available from: http://www.rdehospital.nhs.uk/prof/molecular_genetics/tests/Full_Test_List.htm accessed 13th October 2014.
