# Supplementary material for: Strategies to identify individuals with monogenic diabetes: results of an economic evaluation
Source: BMJ Open. 2020 Mar 18;10(3):e034716. doi: 10.1136/bmjopen-2019-034716 (PMC7150598; doi:10.1136/bmjopen-2019-034716)
Supplement: Supplementary data [file bmjopen-2019-034716supp003.pdf]

Supplementary Data 3: Test-related parameters

Table 3A Summary of the tests involved and estimates of sensitivity and specificity used in the economic evaluation

Test-treatment strategy	Tests used	Sensitivity	Specificity	Data sources
Ad Hoc Testing	Clinical referral based on patient characteristics	0·04	0·996	Shields et al[1]; 2011 census data; Clinical study; Unpublished prevalence data
	Genetic test	1	1	Assumption
Clinical Prediction Model Testing	Type 1 clinical prediction model	0·5 - 0·96	0·65 - 0·996	Shields et al[2]. Estimates of sensitivity and specificity depend on the combination of the probability thresholds used from both clinical prediction models.
	Type 2 clinical prediction model	0·8 - 0·99	0·73 - 0·99	Shields et al[2]. Estimates of sensitivity and specificity depend on the combination of the probability thresholds used from both clinical prediction models.
	Genetic test	1	1	Assumption
Biomarker Testing	UCPCR test	0·94	0·96	Besser et al[3]
	Autoantibody test	0·99	0·82	McDonald et al[4]
	Genetic test	1	1	Assumption
All Testing	Genetic test	1	1	Assumption

UCPCR, urinary c-peptide to creatinine ratio

Peters JL, *et al. BMJ Open* 2020; 10:e034716. doi: 10.1136/bmjopen-2019-034716

Table 3B Sensitivity and specificity of the Ad Hoc Testing strategy by regions in the UK

Region	Sensitivity	Specificity
Northern Ireland[a]	0.038	0.996
Wales	0.044	0.998
Scotland	0.132	0.988
England	0.086	0.993
South West England	0.196	0.977
South East England	0.080	0.995
London	0.049	0.995
East England	0.060	0.996
West Midlands England	0.077	0.994
East Midlands England	0.074	0.995
Yorkshire/Humberside England	0.084	0.996
North East England	0.122	0.994
North West England	0.074	0.995
UK	0.087	0.993
England and Wales	0.084	0.993

[a]Used in base case analysis

References

1. Shields BM, Hicks S, Shepherd MH, et al. Maturity-onset diabetes of the young (MODY): how many cases are we missing? *Diabetologia* 2010;53:2504-08.
2. Shields BM, McDonald TJ, Ellard S, et al. The development and validation of a clinical prediction model to determine the probability of MODY in patients with young-onset diabetes. *Diabetologia* 2012 [published Online First: 5th January 2012]
3. Besser REJ, Shepherd MH, McDonald TJ, et al. Urinary c-peptide-to-creatinine ratio is a practical outpatient tool for identifying hepatocyte nuclear factor 1-α/hepatocyte nuclear factor 4-α maturity-onset diabetes of the young from long-duration type 1 diabetes. *Diabetes Care* 2011;34:1-6.
4. McDonald TJ, Colclough K, Brown R, et al. Islet autoantibodies can discriminate maturity-onset diabetes of the young (MODY) from type 1 diabetes. *Diabetic Medicine* 2011;28:1028-33.
