# Supplementary material for: Strategies to identify individuals with monogenic diabetes: results of an economic evaluation
Source: BMJ Open. 2020 Mar 18;10(3):e034716. doi: 10.1136/bmjopen-2019-034716 (PMC7150598; doi:10.1136/bmjopen-2019-034716)
Supplement: Supplementary data [file bmjopen-2019-034716supp004.pdf]

Supplementary Data 4: Cost parameters

Table 4A Summary of the costs considered for each strategy

	No Testing	Ad Hoc Testing	Clinical Prediction Model Testing	Biomarker Testing	All Testing
Diabetes-specific consultations	o	o	o	o	o
Current treatment	o	o	o	o	o
HBGM on current treatment	o	o	o	o	o
Blood test (for genetic test or autoantibody testing)		o	o	o	o
UCPCR test				o	
Autoantibody test				o	
Genetic test		o	o	o	o
Treatment transfer assistance[a]		o	o	o	o
New treatment		o	o	o	o
HBGM on new treatment		o	o	o	o
Long-term management	o	o	o	o	o

[a]Includes telephone calls with nurse and visit(s) to GP for changes in treatment during 12 month follow-up. UCPCR, urinary c-peptide to creatinine ratio; HBGM, home blood glucose monitoring

Table 4B Costs of testing associated with the strategies

Cost	Value (£, 2018)	Source
GP nurse time for collecting blood sample	£6	10 minutes at £36 per 1hr GP nurse patient contact time[1]
Genetic test for GCK mutation	£350	Sanger sequence analysis from UK referral centre[2]
Genetic test for HNF1/4A mutation	£450	Sanger sequence analysis from UK referral centre[2]
Genetic test for known mutation	£100	Sanger sequence analysis from UK referral centre[2]
Nurse time for successful treatment transfer	£24	Four 10 minute phone calls (expert opinion) at £36 per 1hr GP nurse patient contact time[1]
GP time for informing patient of genetic test result and treatment change	£28	Cost of GP consultation[1]
UCPCR pack	£3·90	Postage
UCPCR test	£10·50	RD&E laboratory[2]
Autoantibody test	£20	RD&E laboratory[2]

UCPCR, urinary c-peptide to creatinine ratio

Peters JL, *et al. BMJ Open* 2020; 10:e034716. doi: 10.1136/bmjopen-2019-034716

Table 4C Cost estimates (£, 2018) used in the IMS CDM model

Event	Cost (£, 2018)	Source
**CVD complications**		
Myocardial infarction (MI) in 1st year of MI	£7,550	Clarke[3]
Second and subsequent yrs after an MI	£1,250	Clarke[3]
Angina in 1st year of angina	£250	Ward[4]
Second and subsequent yrs after an angina	£200	Ward[4]
Congestive heart failure (CHF) in 1st year of CHF	£3,500	Clarke[5]
Second and subsequent yrs after a CHF	£500	Clarke[5]
Stroke in 1st year of stroke	£4,600	Clarke[3]
Second and subsequent yrs after a stroke	£850	Clarke[3]
Stroke death within 30 days of stroke	£6,350	Clarke[3]
Peripheral vascular disease (PVD) in 1st year of PVD	£1,150	Clarke [5]
Second and subsequent yrs after a PVD	£450	Clarke[5]
**Renal complications**		
Hemodialysis in 1st year of needing hemodialysis	£43,500	Baboolal[6]
Hemodialysis in second & subsequent yrs of needing hemodialysis	£43,500	Baboolal [6]
Peritoneal dialysis in 1st year of needingperitoneal dialysis	£24,250	Baboolal [6]
Peritoneal dialysis in second & subsequent yrs of needing peritoneal dialysis	£24,250	Baboolal [6]
Renal transplant in 1st year of needing renal transplant	£13,100	NHS Schedule Reference costs[7]; Wight [8]
Renal transplant in second & subsequent yrs of needing renal transplant	£7,050	Wight[8]
**Acute events**		
Major hypoglyceamic event	£200	Hammer[9]
Minor hypoglyceamic event	£0	Would not require medical assistance
Ketoacidosis event	£1,250	Scuffham[10]
Lactic acid event	£2,500	Curtis[11]
Edema onset	£50	Curtis[11]
Edema follow-up	£0	Assume no follow-up
**Eye disease**		
Laser treatment	£100	NHS Schedule Reference costs[7]
Cataract operation	£800	NHS Schedule Reference costs[7]
Following cataract operation	£550	Clarke[3]
Blindness in the yr of onset	£7,250	Mitchell[12]
Blindness in the following yrs	£7,250	Mitchell[12]
**Neuropathy/foot ulcer**		
Neuropathy in the first yr	£150	BNF[13]
Neuropathy in subsequent yrs	£150	BNF[13]
Amputation (one-off cost)	£7,950	Kerr[14]

Amputation prosthesis (one-off cost)	£3,200	Kerr [14]
Gangrene treatment	£2,700	
After a healed ulcer	£0	Assumption
Infected ulcer	£4,050	Kerr [14]
Standard uninfected ulcer	£4,050	Kerr [14]
Healed ulcer in those with an amputation history	£0	Assumption
**Other**		
Statins	£0	NICE guidance and BNF[13]
Aspirin	£0	NICE guidance and BNF[13]
Angiotensin-converting enzyme (ACE)	£0	BNF
Screening for microalbuminuria	£0	NICE[15]
Screening for gross proteinuria	£0	Assume as for MA
Stopping ACEs due to side effects	£0	Assumptions
Eye screening	£50	NICE [15]
Foot screening programme	£100	NICE[16] and Curtis [17]
Non-standard ulcer treatment (e.g. Regranex)	£0	Assumptions
Anti-depression treatment	£0	Assumptions
Screening for depression	£0	Assumptions

Table 4D Annual number of primary care consultations (taken from Currie et al 2010[18])

Type of consultation	Type 1	Type 2	Type 1 control	Type 2 control	Cost per consultation
GP surgery	7·3	8·7	4·5	5·4	£34
GP home visit	0·3	0·6	0·1	0·4	£41
GP telephone	0·5	0·7	0·3	0·4	£20
Community nurse clinic	0·9	1·5	0·3	0·6	£12
Total cost	£278	£349	£165	£213	
Additional cost over controls	£113	£136			

Peters JL, *et al. BMJ Open* 2020; 10:e034716. doi: 10.1136/bmjopen-2019-034716

References

1. Curtis LA, Burns A. Unit costs of health and social care 2017: Personal Social Services Research Unit, University of Kent, 2017.
2. Royal Devon and Exeter NHS Foundation Trust. [Available from: http://www.rdehospital.nhs.uk/prof/molecular_genetics/tests/Full_Test_List.htm accessed 13th October 2014.
3. Clarke P, Gray A, Legood R, et al. The impact of diabetes-related complications on healthcare costs: results from the United Kingdom Prospective Diabetes Study (UKPDS 65). *Diabetic Medicine* 2003;20:442-50.
4. Ward S, Lloyd Jones M, Pandor A, et al. Statins for the prevention of coronary events. Technology assessment report commissioned by the HTA programme on behalf of the National Institute for Clinical Excellence, 2005.
5. Clarke PM, Glasziou P, Patel A, et al. Event rates, hospital utilization, and costs associated with major complications of diabetes: a multicountry comparative analysis. *PLoS Medicine* 2010;7(2)
6. Baboolal K, McEwan P, Sondhi S, et al. The cost of renal dialysis in a UK setting - a multicentre study. *Nephrology Dialysis Transplantation* 2008;23(6):1982-89.
7. Department of Health. National Schedule of Reference Costs 2012-2013, 2013.
8. Wight J, Chilcott J, Holmes M, et al. The clinical and cost-effectiveness of pulsatile machine perfusion versus cold storage of kidneys for transplantation retrieved from heart-beating and non-heart-beating donors. *Health Technology Assessment* 2003;7(25)
9. Hammer M, Lammert M, Mejias SM, et al. Costs of managing severe hypoglycaemia in three European countries. *Journal of Medical Economics* 2009;12(4)
10. Scuffham P, Carr L. The cost-effectiveness of continuous subcutaneous insulin infusion compared with multiple daily injections for the management of diabetes. *Diabetic Medicine* 2003;20(7):586-93.
11. Curtis L. Unit Costs of Health and Social Care 2013, 2013.
12. Mitchell P, Annemans L, Gallagher M, et al. Cost-effectiveness of ranibizumab in treatment of diabetic macular oedema (DME) causing vision impairment: evidence from the RESTORE trial. *British Journal of Opthalmology* 2011;early online
13. British National Formulary 67. BNF 67 (May 2014) 2014 [
14. Kerr M. Foot care for people with diabetes: the economic case for change: NHS Diabetes, 2012.
15. National Institute for Health and Clinical Excellence. Quality Standards Programme. NICE cost impact and commissioning assessment for diabetes in adults, 2011.
16. National Institute for Health and Clinical Excellence. Diabetes footcare commissioning and benchmarking tool, 2012.
17. Curtis L. Unit Costs of Health and Social Care 2011: PSSRU, 2012.
18. Currie CJ, Gale EAM, Poole CD. Estimation of primary care treatment costs and treatment efficacy for people with type 1 and Type 2 diabetes in the United Kingdom from 1997 to 2007. *Diabetic Medicine* 2010;27(8):938-48.

Peters JL, *et al. BMJ Open* 2020; 10:e034716. doi: 10.1136/bmjopen-2019-034716
