# Supplementary material for: Strategies to identify individuals with monogenic diabetes: results of an economic evaluation
Source: BMJ Open. 2020 Mar 18;10(3):e034716. doi: 10.1136/bmjopen-2019-034716 (PMC7150598; doi:10.1136/bmjopen-2019-034716)
Supplement: Author's manuscript [file bmjopen-2019-034716.draft_revisions.pdf]

BMJ Open

BMJ Open is committed to open peer review. As part of this commitment we make the peer review history of every article we publish publicly available.

When an article is published we post the peer reviewers' comments and the authors' responses online. We also post the versions of the paper that were used during peer review. These are the versions that the peer review comments apply to.

The versions of the paper that follow are the versions that were submitted during the peer review process. They are not the versions of record or the final published versions. They should not be cited or distributed as the published version of this manuscript.

BMJ Open is an open access journal and the full, final, typeset and author-corrected version of record of the manuscript is available on our site with no access controls, subscription charges or pay-per-view fees (http://bmjopen.bmj.com).

If you have any questions on BMJ Open's open peer review process please email info.bmjopen@bmj.com

BMJ Open

Strategies to Identify Individuals with Monogenic Diabetes: Results of an Economic Evaluation

Journal:	*BMJ Open*
Manuscript ID	bmjopen-2019-034716
Article Type:	Original research
Date Submitted by the Author:	02-Oct-2019
Complete List of Authors:	Peters, Jaime; University of Exeter Medical School, Exeter Test Group<br>Anderson, Rob; University of Exeter, ESMI (Evidence Synthesis & Modelling for Health Improvement)<br>Shields, Beverley; University of Exeter,<br>King, Sophie; Cardiff University, Centre for Trials Research<br>Hudson, Michelle; University of Exeter Medical School, Institute of Biomedical and Clinical Science<br>Shepherd, Maggie; University of Exeter Medical School, NIHR Clinical Research Facility<br>McDonald, Timothy; Royal Devon and Exeter NHS Foundation Trust, ; University of Exeter, NIHR Exeter Clinical Research Facility<br>Pearson, Ewan; University of Dundee, Division of Molecular & Clinical Medicine<br>Hattersley, Andrew; University of Exeter Medical School, Institute of Biomedical Science<br>Hyde, Chris; University of Exeter Medical School, Exeter Test Group
Keywords:	pharmacogenetics, monogenic diabetes, economic evaluation, decision analytic modelling, tests

SCHOLARONE™
Manuscripts

I, the Submitting Author has the right to grant and does grant on behalf of all authors of the Work (as defined in the below author licence), an exclusive licence and/or a non-exclusive licence for contributions from authors who are: i) UK Crown employees; ii) where BMJ has agreed a CC-BY licence shall apply, and/or iii) in accordance with the terms applicable for US Federal Government officers or employees acting as part of their official duties; on a worldwide, perpetual, irrevocable, royalty-free basis to BMJ Publishing Group Ltd ("BMJ") its licensees and where the relevant Journal is co-owned by BMJ to the co-owners of the Journal, to publish the Work in this journal and any other BMJ products and to exploit all rights, as set out in our licence.

The Submitting Author accepts and understands that any supply made under these terms is made by BMJ to the Submitting Author unless you are acting as an employee on behalf of your employer or a postgraduate student of an affiliated institution which is paying any applicable article publishing charge ("APC") for Open Access articles. Where the Submitting Author wishes to make the Work available on an Open Access basis (and intends to pay the relevant APC), the terms of reuse of such Open Access shall be governed by a Creative Commons licence – details of these licences and which Creative Commons licence will apply to this Work are set out in our licence referred to above.

Other than as permitted in any relevant BMJ Author's Self Archiving Policies, I confirm this Work has not been accepted for publication elsewhere, is not being considered for publication elsewhere and does not duplicate material already published. I confirm all authors consent to publication of this Work and authorise the granting of this licence.

For peer review only

Strategies to Identify Individuals with Monogenic Diabetes: Results of an Economic Evaluation

Jaime L Peters[1,2]*, Rob Anderson[3], Beverley M Shields[4], Sophie King[4,#a], Michelle Hudson[4], Maggie Shepherd[4], Timothy J McDonald[4], Ewan R Pearson[5], Andrew T Hattersley[4], Chris J Hyde[1]

[1]Exeter Test Group, University of Exeter Medical School, University of Exeter, South Cloisters, St Luke's Campus, Exeter, UK

[2]Collaboration for Leadership in Applied Health Research and Care South West Peninsula (NIHR CLAHRC South West Peninsula), University of Exeter Medical School, University of Exeter, South Cloisters, St Luke's Campus, Exeter, UK

[3]Evidence Synthesis & Modelling for Health Improvement, University of Exeter Medical School, University of Exeter, South Cloisters, St Luke's Campus, Exeter, UK

[4]NIHR Exeter Clinical Research Facility, University of Exeter Medical School, University of Exeter, Barrack Road, Exeter, UK

[5]Division of Cardiovascular & Diabetes Medicine, Medical Research Institute, University of Dundee, UK

[#a] Current Address: Peninsula Clinical Trials Unit, Plymouth University Peninsula Schools of Medicine and Dentistry, Plymouth Science Park, Plymouth, PL6 8BX, UK

* Corresponding author, email: j.peters@exeter.ac.uk

For peer review only

Abstract

Objectives: To evaluate and compare the lifetime costs associated with strategies to identify individuals with monogenic diabetes and change their treatment to more appropriate therapy.

Design: A decision analytic model from the perspective of the National Health Service (NHS) in England and Wales was developed and analysed. The model was informed by the literature, routinely collected data and a clinical study conducted in parallel with the modelling.

Setting: Secondary care in the UK.

Participants: Simulations based on characteristics of patients diagnosed with diabetes <30 years old.

Interventions: Four test-treatment strategies to identify individuals with monogenic diabetes in a prevalent cohort of diabetics diagnosed under the age of 30 years were modelled: clinician-based genetic test referral, targeted genetic testing based on clinical prediction models, targeted genetic testing based on biomarkers, and blanket genetic testing. The results of the test-treatment strategies were compared to a strategy of no genetic testing.

Primary and secondary outcome measures: Discounted lifetime costs, proportion of cases of monogenic diabetes identified.

Results: Based on current evidence, strategies using clinical characteristics or biomarkers were estimated to save approximately £100-£200 per person with diabetes over a lifetime

For peer review only

For peer review only - http://bmjopen.bmj.com/site/about/guidelines.xhtml

compared to no testing. Sensitivity analyses indicated that the prevalence of monogenic diabetes, the uptake of testing, and the frequency of home blood glucose monitoring had the largest impact on the results (ranging from savings of £400 to £50 per person), but did not change the overall findings. The model is limited by many model inputs being based on very few individuals, and some long-term data informed by clinical opinion.

Conclusions: Costs to the NHS could be saved with targeted genetic testing based on clinical characteristics or biomarkers. More research should focus on the economic case for the use of such strategies closer to the time of diabetes diagnosis.

Strengths and limitations of this study:

- This is the first UK study to evaluate and compare the costs of testing strategies to identify individuals with monogenic diabetes and change their treatment to more appropriate therapy.
- Although informed by the current evidence base, due to rarity of monogenic diabetes, many of the parameters were based on low numbers of patients.

Funding statement: This study was supported by the Department of Health and Wellcome Trust Health Innovation Challenge Award (HICF-1009-041 and WT-091985). JP is partly supported by the NIHR Collaboration for Leadership in Applied Health Research and Care for the South West Peninsula (PenCLAHRC). BMS, MH and ATH are core members of the National Institute for Health Research (NIHR) Exeter Clinical Research Facility. TJM is

For peer review only - http://bmjopen.bmj.com/site/about/guidelines.xhtml

supported by an NIHR Chief Scientist Office Fellowship. ATH is a Wellcome Trust Senior Investigator and an NIHR Senior Investigator. ERP is a Wellcome Trust New Investigator.

Keywords: costs, decision analytic model, economic evaluation, monogenic diabetes, pharmacogenetics, tests

For peer review only

For peer review only - http://bmjopen.bmj.com/site/about/guidelines.xhtml

Background

Monogenic diabetes is a form of diabetes caused by a mutation in a single gene, which is inherited in an autosomal dominant manner[1]. Therefore a child of an individual with monogenic diabetes has a 50% chance of inheriting the mutation (assuming the child's other parent does not have the mutation). Mutations in glucokinase (*GCK*), hepatocyte nuclear factor 1 alpha (*HNF1A*) and hepatocyte nuclear factor 4 alpha (*HNF4A*) genes are the most common forms of monogenic diabetes.[2] Individuals with mutations in the *GCK gene* have persistently moderately raised blood glucose levels from birth, that is rarely detrimental to health[3] and does not respond to treatment.[4] Therefore individuals with mutations in the *GCK* gene can be successfully treated by diet[4]. Individuals with *HNF1A* or *HNF4A* mutations have blood glucose levels which increase over time and can be successfully treated with sulphonylureas[5] but may, eventually, require insulin treatment.[6]

The minimum prevalence of monogenic diabetes in the UK has been estimated as 108 cases per million.[7] As it usually presents by 25-30 years of age,[1 2 8] individuals are often misdiagnosed with type 1 diabetes, and receive insulin treatment when less invasive and less costly treatment is more appropriate.

The National Health Service (NHS) in England and Wales currently has no national guidelines for identifying individuals with monogenic diabetes. Realistic strategies are available ranging from genetic testing of all individuals with diabetes to targeted genetic testing based on clinical characteristics[9] or biochemical[10] and immunological[11] tests. We report a UK-based economic evaluation of these realistic strategies to identify individuals with monogenic diabetes (defined here as mutations in *GCK*, *HNF1A* or *HNF4A* genes). The development of

For peer review only - http://bmjopen.bmj.com/site/about/guidelines.xhtml

the model-based economic evaluation has been published elsewhere.[12] The economic evaluation was undertaken alongside a clinical study whose aims included (i) investigating the prevalence of monogenic diabetes within two areas of the UK, and (ii) measuring the effects of a change of treatment following a positive diagnosis of monogenic diabetes. The clinical study recruited 1407 individuals who were diagnosed with diabetes <30 years old and who were <50 years old at recruitment[13]. Prospective quality of life (using the EQ-5D Index, a generic measure of health outcome[14]) and glycated haemoglobin (HbA1c) data for 45 individuals who were diagnosed with monogenic diabetes within the geographical areas of the clinical study were collected until 12 months after the genetic test result. Although the clinical study collected data on clinical outcomes, it was not designed, nor powered, to detect small changes in clinical outcomes. In the event no statistically significant change in the EQ-5D Index or HbA1c before and 12 months after changing treatment was observed making it impossible to confirm or refute the clinically suspected benefit of changing treatment in persons found to have monogenic diabetes, but on inappropriate treatment. Thus, only costs are considered in this economic evaluation, making this a conservative analysis of the testing strategies if patient benefit does occur. The implications of this are considered in the discussion.

The aim of this analysis is to evaluate and compare the lifetime costs of different realistic strategies in the NHS to identify individuals with monogenic diabetes and change their treatment to more appropriate therapy. This economic evaluation has been reported in line with CHEERS, the Consolidated Health Economic Evaluation Reporting Standards[15].

For peer review only - http://bmjopen.bmj.com/site/about/guidelines.xhtml

Materials and Methods

Model overview

A hybrid decision model was developed from the perspective of the NHS in England and Wales. A decision tree was developed in MicroSoft Excel to estimate the short-term (16 months) costs, which allowed a maximum of 4 months from referral to testing to change of treatment (for those identified as having monogenic diabetes), plus 12 months follow-up (coinciding with the accompanying clinical study). The IMS CORE Diabetes Model (IMS CDM) version 8.5[16] was used to estimate the lifetime costs associated with the strategies. Expert consultation and explicit critical appraisal of existing long-term diabetes models helped to inform the structure of the decision model and choice of the IMS CDM (see Peters et al[12] for more detail on model development). Evidence to inform the model came from a number of sources including published and unpublished data and clinical opinion. Details on the evidence used in the model are given below.

Strategies and comparator

Five strategies for identifying monogenic diabetes in individuals who were diagnosed with diabetes under the age of 30 years were defined: no genetic testing ("No Testing"), clinician-based genetic test referral ("Ad Hoc Testing"), targeted genetic testing based on clinical prediction models[9] ("Clinical Prediction Model Testing") or biochemical (urinary c-peptide to creatinine ratio, UCPCR[10]) and immunological (islet autoantibodies[11]) test results ("Biomarker Testing"), blanket genetic testing ("All Testing").

The No Testing strategy is the comparator for all other strategies, as it represents the current policy within England and Wales where there is no guidance on the identification of

For peer review only - http://bmjopen.bmj.com/site/about/guidelines.xhtml

individuals with monogenic diabetes. Thus, in this strategy all individuals remain on the diabetes treatment they were receiving at the start of the model, regardless of whether they truly have monogenic diabetes or not.

The Ad Hoc Testing strategy assumes no systematic referral of individuals for monogenic diabetes genetic testing. Instead, individuals are referred on an *ad hoc* basis depending on the awareness of local clinicians of monogenic diabetes (see Fig 1). Data on referral rates for monogenic diabetes genetic testing in the UK[7] were used to calculate estimates of sensitivity and specificity of *ad hoc* referral.

In the Clinical Prediction Model Testing strategy, it is assumed that an individual GP would complete the online monogenic diabetes prediction model (http://www.diabetesgenes.org/content/mody-probability-calculator [9]) to calculate a probability of the individual having monogenic diabetes (see Fig 1). Depending on the probability of the individual having monogenic diabetes as calculated from the prediction model, the GP would then refer them for monogenic diabetes genetic testing or not. Two versions of the prediction model exist, one to distinguish type 1 diabetes from monogenic diabetes (version 1) and the other to distinguish type 2 diabetes from monogenic diabetes (version 2). If the individual is currently receiving insulin, then version 1 of the prediction model is used, otherwise version 2 is used. For each version of the prediction model, nine thresholds are simulated in the decision model. Thus, the Clinical Prediction Model Testing strategy can be evaluated at 81 thresholds (9 from version 1 x 9 from version 2) for the simulated population. The decision model can then be used to identify the probability threshold for the prediction model that maximises the costs saved using the Clinical Prediction Model Testing strategy compared to the No Testing strategy.

For peer review only - http://bmjopen.bmj.com/site/about/guidelines.xhtml

In the Biomarker Testing strategy individuals receive biochemical and/or immunological tests depending on their demonstrated ability to produce insulin (see Fig 2). If individuals are currently receiving insulin treatment, they are offered a UCPCR test to determine whether they are producing insulin or not[10]. Those with a positive UCPCR test are then offered a test for glutamic acid decarboxylase (GAD) and islet antigen2 (IA2) autoantibodies[11]. If individuals are not currently receiving insulin treatment it is assumed they can produce their own insulin and so do not require a UCPCR test. Instead, those individuals not on insulin treatment are offered a test for GAD and IA2 autoantibodies. The aim of the GAD and IA2 autoantibodies test is to rule out those individuals with type 1 diabetes who are still producing insulin (i.e. in the 'honeymoon' period). Individuals not showing the presence of autoantibodies are then offered the monogenic diabetes genetic test. In the All Testing strategy, all individuals are offered monogenic diabetes genetic testing (see Fig 1).

[Fig 1 Simplified model structure for the Ad Hoc Testing, Clinical Prediction Model Testing and All Testing strategies.]

[Fig 2 Simplified model structure for the Biomarker Testing strategy]

Model input parameters

Population characteristics

The main analysis (modelled Cohort 1) simulated a prevalent cohort of individuals in England and Wales who were diagnosed with diabetes when <30 years old and were <50 years old at the start of the model. The prevalence of monogenic diabetes assumed in this

For peer review only - http://bmjopen.bmj.com/site/about/guidelines.xhtml

cohort is 2.4% (*GCK* mutation 0.7%, *HNF1A* mutation 1.5%, *HNF4A* mutation 0.2%). A subgroup analysis (modelled Cohort 2) was undertaken to represent a future incident cohort who would have had a diagnosis of diabetes for a shorter duration than those in Cohort 1. Cohort 2 is defined as individuals diagnosed with diabetes when <30 years old and who were <30 years old at the start of the model, leading to a prevalence of 2.2% having monogenic diabetes. All information relevant to Cohort 2, including parameter values and results, are in Supplementary Data 1. Further data on the prevalence and characteristics of Cohort 1 are given in Supplementary Data 2.

Test characteristics

Details of the test sensitivity and specificity used in the model are shown in Supplementary Data 3. To calculate the sensitivity and specificity of referral for monogenic diabetes genetic testing in the Ad Hoc Testing strategy, four datasets were used:

- diabetes prevalence from unpublished data for Tayside
- estimates of total population by age and area from national census[17]
- monogenic diabetes prevalence from the accompanying clinical study[13]
- monogenic diabetes genetic test referral rates[7].

The referral rates for monogenic diabetes genetic testing varied across the UK, with higher referral rates in areas where there is a strong research interest in monogenic diabetes, e.g. the South West of England, and Scotland. Estimates of sensitivity and specificity varied from sensitivity of 0.038 and specificity of 0.996 (Northern Ireland) to sensitivity 0.196 and specificity 0.977 (South West of England), see Supplementary Data 3. To account for the general low rates of referral in the UK, we assumed the referral rates for one of the lowest areas, Northern Ireland. In sensitivity analyses, data from all individual regions were used to

For peer review only - http://bmjopen.bmj.com/site/about/guidelines.xhtml

For peer review only

estimate sensitivity and specificity for the Ad Hoc Testing strategy. However, the cost of increased awareness in one area compared to other areas is not known, and so it is not possible to estimate the additional cost of increased awareness of monogenic diabetes in the Ad Hoc Testing strategy, such as the South West of England and Scotland.

For the Clinical Prediction Model Testing strategy the probability thresholds of 10-90% for the two versions of the test were taken from Shields et al[9], with sensitivity ranging from 0.5-0.99 and specificity ranging from 0.65-0.996. All 81 combinations of probability thresholds were evaluated in the decision model. No adjustments were made to the clinical prediction model as the population on which it would be applied (individuals with diabetes in England and Wales) is very similar to that on which it is based. In the Biomarker Testing strategy, sensitivity of 0.94 and specificity of 0.96 for the UCPCR test was used based on a UCPCR cut-off of ≥0.2nmol/mmol to discriminate individuals with *HNF1A* and *HNF4A* mutations who were insulin treated from individuals with type 1 diabetes[10]. Besser et al did not report on the sensitivity and specificity of this cut-off to discriminate insulin-treated type 2 from *GCK*, *HNF1A* and *HNF4A* mutations, or to discriminate type 1 from *GCK* mutations. Since use of a different UCPCR cut-off for type 1 or insulin-treated type 2 would be difficult in practice (Besser et al[10]), we assumed that the UCPCR cut-off of ≥ 0·2nmol/mmol could be used to discriminate type 1 from insulin-treated type 2, *HNF1A* and *HNF4A* mutations. Furthermore, Besser et al report that UCPCR cannot be used to discriminate *GCK* from *HNF1A* and *HNF4A* mutations. Thus, we assume that the UCPCR cut-off of ≥0.2nmol/mmol can be used to discriminate type 1 diabetes from insulin-treated type 2, *GCK*, *HNF1A* and mutations. The impact on the model results of using different estimates of sensitivity and specificity is assessed in sensitivity analyses. Data from McDonald et al[11] were used to inform the

For peer review only - http://bmjopen.bmj.com/site/about/guidelines.xhtml

sensitivity and specificity for the GAD and IA2 autoantibody tests (see Supplementary Data 3). For all testing strategies, individuals referred for the monogenic diabetes genetic test were either tested for mutations in the *GCK* gene only, the *HNF1A* and *HNF4A* genes together, or all three genes (see Supplementary Data 2).

Uptake and repeat tests

Using data from the accompanying clinical study, for Cohort 1, it was assumed that 8·2% of individuals would decline the offer of genetic testing (6.9% for Cohort 2). This percentage was applied to all of the strategies where genetic testing was an option. For the Biomarker Testing strategy it was assumed that 11·9% for Cohort 1 (12.8% for Cohort 2) of individuals offered the UCPCR test and 8·2% for Cohort 1 (6.9% for Cohort 2) of individuals offered the autoantibody test would not accept. Estimates of the number of repeat tests required for both cohorts in the Biomarker Testing strategy are reported in Supplementary Data 2.

Family genetic testing

It was assumed in the model that identification of an individual with monogenic diabetes from any of the defined strategies would lead to first degree family members (who fit the defined cohort) also being genetically tested. Once individuals identified from the testing strategies have had the genetic test and are found to have monogenic diabetes, their family members receive the monogenic diabetes genetic tests. In Cohort 1, it was assumed that for every 10 individuals identified by the testing strategies as having monogenic diabetes, a further 6·3 family members are genetically tested, with 5.9 of these assumed to have the mutation (based on UK referral rate data[7]). These ratios were applied to the Ad Hoc Testing, Clinical Prediction Model Testing and Biomarker Testing strategies.

For peer review only - http://bmjopen.bmj.com/site/about/guidelines.xhtml

Treatment for diabetes

The treatment pattern assumed at the model start is given in Supplementary Data 2. These data are from the accompanying clinical study where the treatment pattern for those truly having monogenic diabetes is based on just 45 individuals. The impact on the model results of the type of treatment at the start of the model is assessed in sensitivity analyses. Only individuals with a positive genetic test were offered a treatment change; which was cessation of diabetes treatment for those with the *GCK* mutation or to sulphonylureas for individuals with the *HNF1A* or *HNF4A* mutations. Data from the clinical study informed the likely treatment pattern once individuals are diagnosed with monogenic diabetes. For Cohort 1, at 1 month after treatment change it was assumed that 86% of individuals with *HNF1A* or *HNF4A* mutations were receiving a more appropriate treatment, at 3 months this was 86%, at 6 months this was 89% and at 12 months this was 77% (see Supplementary Data 2). Some individuals having a positive genetic test result may not successfully change to sulphonylurea treatment alone and may continue to receive insulin.[18] For individuals with *HNF1A* or *HNF4A* mutations it was assumed that they would require insulin treatment eventually, and how much insulin and when they would start taking it would depend upon whether they had previously received sulphonylureas and progressed to insulin or had started on insulin initially. As no data are available two experts in monogenic diabetes (ATH and EP) were consulted for their opinion (see Supplementary Data 2). Based on data from the accompanying clinical study it was assumed that 93% of individuals identified to have the GCK mutation, would successfully stop all diabetes treatment.

For peer review only - http://bmjopen.bmj.com/site/about/guidelines.xhtml

Resource use

The type of NHS costs (£, inflated to 2018 prices using the Hospital and Community Health Services pay an prices index[19]) considered within each strategy are summarised in Supplementary Data 4.

All treatment costs were estimated using the reported doses from the clinical study and the BNF[20]. The costs associated with the tests include costs for the collection of blood and urine samples, costs of the UCPCR and autoantibody tests and genetic test costs. The costs of nurse time spent providing assistance to those individuals with monogenic diabetes who are changing to a more appropriate treatment were also included. See Supplementary Data 4.

The costs associated with home blood glucose monitoring (HBGM) were also included in the model. The frequency of HBGM before and after diagnosis of monogenic diabetes, and any subsequent change in treatment, was estimated from the clinical study for individuals truly having monogenic diabetes (see Supplementary Data 2). Data from the literature were used to inform HBGM frequency in individuals with type 1 and type 2 diabetes[21 22]. It was assumed that individuals who have a *GCK*, *HNF1A* or *HNF4A* mutation, but did not have a genetic test or change treatment would have the same HBGM frequency as at the start of the model. Costs of HBGM were based on use of the Accu-Check Aviva meter (£16.09 for 50 strips[20]).

The costs of diabetes-related complications for individuals with type 1 diabetes, type 2 diabetes, and *HNF1A* or *HNF4A* mutations were identified from reviewing the published literature and using data from the National Schedule of Reference Costs 2016/17. Only cost data from the UK were modelled in the IMS CDM (see Supplementary Data 4). The majority of cost estimates from the literature were associated with uncertainty, mainly in inflating

For peer review only - http://bmjopen.bmj.com/site/about/guidelines.xhtml

the costs to 2018 due to the age of the evidence available, therefore all of the long-term costs inputted into the model were rounded to the nearest £50 to avoid spurious precision. It is assumed that individuals with *GCK* mutations do not experience long-term diabetes-related complications[3] and once identified as having a mutation in the GCK gene, they no longer incur the costs of diabetes-specific consultations. Data from Curtis 2017[19] and Currie et al 2010[23] were used to inform the costs of diabetes-specific consultations (see Supplementary Data 4).

Survival

It was assumed that individuals with *GCK* mutations have the same mortality rate as the general population[17]. Due to limited data on long-term complications and mortality of individuals with *HNF1A* and *HNF4A* mutations, it was assumed that these individuals have the same pattern of long-term complications and mortality as individuals with type 1 diabetes as modelled in the IMS CDM.

Model outcomes

All costs (£, 2018) beyond the first year are discounted at a rate of 3·5% per annum to account for the preference for deferring future costs in economic evaluations.[24] Discounted and undiscounted total costs are reported in the results section alongside the estimated discounted incremental costs per person with diabetes over a lifetime for each strategy compared to the No Testing strategy and the proportion of monogenic diabetes cases identified by each strategy.

For peer review only - http://bmjopen.bmj.com/site/about/guidelines.xhtml

Analysis

The results of a "base case" analysis are presented, but due to the uncertainty surrounding many of the parameter estimates alternative combinations of assumptions may be equally plausible. Therefore, wide-ranging one-way sensitivity and threshold analyses have been conducted to explore the different sources of uncertainty. Details of the sensitivity and threshold analyses undertaken for Cohort 1 can be found in Supplementary Data 2 (see Supplementary Data 1 for details on Cohort 2 analyses). In contrast to our planned analysis[12], we decided not to do a probabilistic analysis because important structural uncertainties in this model could not be fully captured by a probabilistic analysis (it would therefore be misleading). There was no patient and public involvement in the development or analysis of the model.

Results

Cohort 1: diagnosed <30 years old, <50 years old at start of model

For the "base case" analysis, the total discounted costs per person with diabetes over a lifetime were estimated to be £53,500 to £54,000 depending on the strategy used (see Table 1). The All Testing strategy was estimated as the most costly (£54,000), the cheapest options were the Clinical Prediction Model Testing (where the probability thresholds were chosen to maximise costs saved compared to No Testing) and Biomarker Testing strategies (£53,600). The No Testing and Ad Hoc Testing strategies were both estimated as £53,700 per person with diabetes over a lifetime. The Ad Hoc Testing strategy was estimated to identify very few cases of monogenic diabetes (6%) compared to the All Testing strategy

For peer review only - http://bmjopen.bmj.com/site/about/guidelines.xhtml

which was estimated to identify 92% of monogenic diabetes cases. No more than 92% of monogenic diabetes cases can be identified by any strategy due to the assumption that 8% of individuals will not accept an offer of genetic testing for monogenic diabetes. Family testing boosts the detection of monogenic diabetes cases to 92% in the Clinical Prediction Model Testing and Biomarker Testing strategies. The costs saved for these two strategies over the No Testing strategy relate to more individuals getting a monogenic diabetes diagnosis and changing to receive more appropriate treatment which is cheaper and also leads to a reduction in the frequency of HBGM. The All Testing strategy is the most expensive since although more monogenic diabetes diagnoses are made, resulting in fewer treatment and HBGM costs, the costs of genetically testing all individuals diagnosed with diabetes are very high.

Table 1 Summary of the per person lifetime costs[a] and percentage of cases and non-cases genetically tested for each strategy (ordered by increasing cost of strategy)

Strategy	Total undiscounted costs [a]	Total discounted costs [a]	Incremental costs vs No Testing strategy [a]	% who are genetically tested	
				With monogenic diabetes	Without monogenic diabetes
Clinical Prediction Model Testing[b]	£133,200	£53,600	-£100	92	3
Biomarker Testing	£133,300	£53,600	-£100	92	8

For peer review only - http://bmjopen.bmj.com/site/about/guidelines.xhtml

Ad Hoc Testing	£133,500	£53,700	0	6	<1
No Testing	£133,600	£53,700	NA	0	0
All Testing	£133,700	£54,000	£300	92	92

[a] rounded to nearest £100.
[b]probability thresholds chosen to maximise costs saved vs No Testing are 12.6% for type 1 vs monogenic diabetes and 75.5% for type 2 vs monogenic diabetes.

As there are 81 different combinations of probability thresholds for the clinical prediction model, the combination of thresholds which maximises the costs saved for the Clinical Prediction Model Testing strategy have been reported above. In Fig 3, all 81 threshold combinations for the clinical prediction model are shown. The Clinical Prediction Model Testing strategy is estimated to identify 74% or 92% of monogenic diabetes cases depending on the probability threshold combinations used to refer individuals for genetic testing. The lifetime costs saved per person with these threshold combinations compared to No Testing vary from £0 to £150.

[Fig 3. Base case incremental costs (vs No Testing) and the proportion of monogenic diabetes cases identified for each strategy.]

Sensitivity analysis results suggest that the impacts on costs in the different scenarios are insensitive to wide-ranging, plausible changes to key model parameters, (see Figs 4a-4d). No plausible parameter value changes the finding that the Ad Hoc Testing and Clinical Prediction Model Testing strategies are always estimated to save costs compared to the No Testing strategy. Only extreme assumptions on the uptake of genetic and UCPCR testing (just 10% uptake) suggest fewer costs are saved from the Biomarker Testing strategy when compared to the No Testing strategy. Except for assumptions on test uptake, the estimated

For peer review only - http://bmjopen.bmj.com/site/about/guidelines.xhtml

cost savings are in the region of £0-£50 per person over a lifetime for the Ad Hoc Testing strategy (see Fig 4a), £50-£300 for the Clinical Prediction Model Testing strategy (see Fig 4b) and £50-£250 for the Biomarker Testing strategy (see Fig 4c). The All Testing strategy is estimated to cost an additional £150-£350 per person over a lifetime compared to the No Testing strategy except when the cost of the genetic test is assumed to be <60% of its current cost (see Fig 4d).

[Fig 4a. Sensitivity analyses: incremental costs per person over a lifetime for Ad Hoc Testing strategy vs No Testing strategy.]

[Fig 4b. Sensitivity analyses: incremental costs per person over a lifetime for Clinical Prediction Model Testing strategy vs No Testing strategy.]

[Fig 4c. Sensitivity analyses: incremental costs per person over a lifetime for Biomarker Testing strategy vs No Testing strategy.]

[Fig 4d. Sensitivity analyses: incremental costs per person over a lifetime for All Testing strategy vs No Testing strategy.]

For peer review only

As Figs 4a-4d show, the findings are most sensitive to:

- the estimated prevalence of monogenic diabetes within the cohort – increasing prevalence (from 2.4% in Cohort 1 to 4.8%) leads to greater costs saved for the Ad Hoc Testing, Clinical Prediction Model Testing and Biomarker Testing strategies compared to the No Testing strategy,

For peer review only - http://bmjopen.bmj.com/site/about/guidelines.xhtml

- the uptake of testing - reduced uptake leads to fewer costs saved for all strategies compared to the No Testing strategy,
- the frequency of HBGM pre and post-treatment change - assuming that individuals change their frequency of HBGM by only a small amount after a diagnosis of monogenic diabetes leads to fewer costs saved compared to the No Testing strategy,
- the proportion of individuals with monogenic diabetes who receive insulin before their monogenic diabetes diagnosis – the larger the proportion receiving insulin before being diagnosed as having monogenic diabetes, the greater the costs saved for all strategies compared to No Testing.

Threshold analysis results (see Supplementary Data 2) suggest that when the genetic tests are reduced to approximately 35% of their current costs, the All Testing strategy incurs no additional costs compared to the No Testing strategy. However, in this situation, the Biomarker Testing and Clinical Prediction Model Testing strategies are estimated to save, approximately £150 per person over a lifetime, compared to the No Testing strategy. Reducing the percentage of individuals with monogenic diabetes who are receiving only insulin at the start of the model has little impact on the incremental costs estimated: even if 10% of individuals with *GCK* mutations or 10% of individuals with *HNF1A* or *HNF4A* mutations are on tablets at the start of the model, slight cost savings are still estimated with the Clinical Prediction Model Testing and Biomarker Testing strategies compared to the No Testing strategy (see Figs 4b and 4c).

Threshold analyses specific to the Biomarker Testing strategy demonstrate that once uptake of the UCPCR and autoantibody tests is reduced to less than 70%, the costs saved with the

For peer review only

For peer review only - http://bmjopen.bmj.com/site/about/guidelines.xhtml

Biomarker Testing strategy compared to the No Testing strategy reduce. Costs saved with the Biomarker Testing strategy are most sensitive to reductions in the sensitivity of the UCPCR and autoantibody tests. Increases in the number of repeat urine or blood samples and tests required within the Biomarker Testing strategy have little impact on the estimate of costs saved compared to the No Testing strategy.

Cohort 2: diagnosed <30 years, <30 years at start of model

As in Cohort 1, the Clinical Prediction Model Testing and Biomarker Testing strategies are estimated to save £100 per person with diabetes over a lifetime compared to the No Testing strategy, while the All Testing strategy is assumed to cost an additional £300 compared to the No Testing strategy. When compared to Cohort 1, the Clinical Prediction Model Testing and Biomarker Testing strategies are not estimated to save any more costs because of the trade-off between individuals being less likely to be on insulin prior to genetic testing in Cohort 2 (67% vs 83% in Cohort 1) even though they are more likely to successfully change to sulphonylureas than Cohort 1 (100% vs 79% in Cohort 1). Individuals in Cohort 2 were estimated to monitor their blood glucose less frequently before receiving a diagnosis of monogenic diabetes compared to Cohort 1, and so fewer costs are saved from reducing further the HBGM frequency than is the case for Cohort 1. See Supplementary Data 1 for further results, including sensitivity analyses which suggest that estimates of prevalence and testing uptake have the largest impact on the findings (as for Cohort 1).

Discussion

For peer review only - http://bmjopen.bmj.com/site/about/guidelines.xhtml

The Clinical Prediction Model Testing and Biomarker Testing strategies modelled here have been estimated to be cost saving for identifying individuals with monogenic diabetes and changing their treatment compared to the current practice of no genetic testing. Assumptions about the prevalence of monogenic diabetes within the simulated cohort, the uptake of testing and the frequency of HBGM before and after receiving a diagnosis of monogenic diabetes had the largest impact on the findings, but did not change the overall conclusions that targeted strategies are estimated to save costs compared to the No Testing or All Testing strategies. Data on prevalence and test uptake were taken directly from the accompanying clinical study, which is the first to systematically estimate prevalence of monogenic diabetes in the UK[13]. Information on the frequency of HBGM before and after a diagnosis of monogenic diabetes is based on just a small number of individuals, but is currently the best evidence available.

This is the first UK-based economic evaluation of strategies to identify individuals with monogenic diabetes. A published paper documented the development of the model and the intended analysis,[12] and the minor departures from the protocol have been declared and justified. UK data have been used to inform many of the model inputs, for which there was previously no credible evidence. However, due to the rarity of monogenic diabetes, many inputs specific to individuals with monogenic diabetes are based on very few individuals, especially for Cohort 2, or assumptions. For instance, it was assumed that treatment and HBGM frequency data taken from the clinical study at 12 month follow-up remained constant over time in the model, with additional long-term treatment data informed by clinical opinion. Until longer follow-up data are available, it is unclear what impact these assumptions may have on the model results.

For peer review only - http://bmjopen.bmj.com/site/about/guidelines.xhtml

For peer review only

We simulated 2 cohorts, both based on data from the clinical study. The aim of Cohort 2 was to assess the impact of strategies for identifying monogenic diabetes in individuals more recently diagnosed with diabetes than those in Cohort 1. Although it was anticipated that individuals in Cohort 2 would find it easier to change to more appropriate treatment (because they had not been on their existing treatment for a long time), we actually found that individuals in Cohort 2 were less likely to be on insulin at that point, so costs saved from changing treatment were smaller than for Cohort 1, even though more individuals changed treatment. However this analysis was limited by the low number of participants close to diagnosis for which data were available. Furthermore, the performance of the Clinical Prediction Model Testing and Biomarker Testing strategies are based on prevalent cohorts[9-11] which will impact on their generalisability to an incident cohort (Cohort 2). Thus, there are still many uncertainties associated with the results, including that the IMS CDM has not been validated for monogenic diabetes, so these results should be interpreted with this in mind. Nevertheless, the numerous sensitivity and threshold analyses estimated cost-savings for the Clinical Prediction Model Testing (when choice of thresholds was maximised to save costs) and Biomarker Testing strategies compared to No Testing.

Naylor et al[25] conducted an economic evaluation of genetic testing (akin to our All Testing strategy) for monogenic diabetes in individuals aged 25-40 years who were newly diagnosed with type 2 diabetes compared to no genetic testing from a US health system perspective. Individuals identified as having *HNF1A* or *HNF4A* mutations who successfully transferred to sulphonylureas were assumed a HbA1c reduction of 16.4mmol/mol compared to those not changing treatment (based on 6 individuals at 3 months follow-up after treatment change[26]) and a utility increase of 0·13 for transferring from insulin to sulphonylurea treatment (based

For peer review only - http://bmjopen.bmj.com/site/about/guidelines.xhtml

on evidence from 519 individuals aged 65 years and older with type 2 diabetes[27]). Naylor et al reported a gain of 0·012 quality-adjusted life-years (QALYs) for the testing strategy at an additional cost of $2,400 per person over a lifetime compared to their no testing strategy, resulting in an incremental cost-effectiveness ratio of $205,000 per QALY gained[25]. The additional costs for the genetic testing strategy in Naylor et al[25] are much greater than the All Testing strategy in our evaluation ($2,400 vs £300) because of differences in the populations simulated. In our evaluation a younger diabetes population is assumed, with individuals who truly have monogenic diabetes being more likely to be misdiagnosed with type 1 and receive insulin. The simulated population in Naylor et al is older and explicitly those diagnosed with type 2, therefore are less likely to receive insulin treatment, so have fewer cost savings from changing treatment.

The health impacts assumed by Naylor et al[25] are also different from those observed in our accompanying clinical study. Using the EQ-5D Index, we found little evidence over the 12 month treatment change period for an improvement in utility associated with more appropriate treatment, although the EQ-5D visual analogue scale did suggest an increase in quality of life at 12 months. Furthermore, in the sample of 28 individuals with *HNF1A* or *HNF4A* mutations who successfully changed to sulphonylureas no statistically significant impact on HbA1c at 12 months after treatment change was found (mean difference of 3·43 mmol/mol (95% confidence interval -2·18, 9·04)). Due to the lack of evidence suggesting an effect on quality of life and HbA1c we took the decision to assume there were no differences in quality of life and HbA1c between those identified as having monogenic diabetes and subsequently changing treatment, and those not identified. Our evaluation was conservative, as evidence shows that changing treatment can have a substantial

For peer review only - http://bmjopen.bmj.com/site/about/guidelines.xhtml

beneficial impact on individuals[28 29]. However, generic and relatively simple quality of life measures (e.g. EQ-5D) are likely to be insensitive to the magnitude and type of changes individuals with diabetes might experience when changing to more appropriate treatment. Measuring such changes to quality of life is also limited by the ceiling effect, since these individuals generally constitute a well-controlled, young diabetes population with a good quality of life. Given these limitations we have not considered any reductions in quality of life that may occur during the testing period, especially for those tested but not found to have monogenic diabetes.

The results suggest that within the context of the NHS, the additional costs of genetically testing (a relatively large number of) individuals are likely to be offset by the lifetime savings from the subsequent treatment changes in a very small proportion of individuals. Although the estimated cost-savings are relatively small per person (approximately £100-£200 over a lifetime), assuming there are approximately 200,000 individuals (personal communication) in England and Wales who are <50 years old and have had a diagnosis of diabetes before the age of 30 years, between £20million and £40million could be saved if such strategies are used. To be able to apply these findings to other populations the cost of the testing in particular will need to be updated. If the genetic test costs are significantly higher, then it is unclear whether the Clinical Prediction model Testing and Biomarker Testing strategies could be considered cost-saving, or even cost-neutral. However, further collection of treatment pattern, HBGM frequency, HbA1c and quality of life data for individuals with monogenic diabetes is required to better inform the decision model, especially to model an incident cohort. Additional strategies to better identify those with monogenic diabetes are

For peer review only - http://bmjopen.bmj.com/site/about/guidelines.xhtml

feasible, and in development, but will also require evaluation for their effectiveness and cost-effectiveness.

Conclusions

Targeted strategies to identify individuals with monogenic diabetes and change to more appropriate treatment may be cost saving to the NHS. However, collection of longer-term treatment and frequency of HBGM data would be valuable to reduce the main uncertainties in the modelling. Future work to evaluate the use of genetic testing strategies soon after diagnosis of diabetes would be useful to policy-makers.

Checklist for reporting: see supplementary file for CHEERS checklist.

Data sharing statement: The decision analytic model described in this manuscript is not available due to the IMS CDM being under license for the current study.

Competing interests: The authors declare that they have no competing interests.

Authors' contributions: JP designed the decision model, contributed to data collection, undertook analysis and interpretation of the model results and drafted the manuscript. RA and CH helped design and analyse the decision model, and contributed to the interpretation of the results drafting of the manuscript. BS, MH, MS, TM, EP and AH contributed to the study design and data collection, and commented on the manuscript. SK contributed to data collection and commented on the manuscript.

Acknowledgements: We would like to thank IMS Health for use of the IMS CDM. This research was supported by the National Institute for Health Research (NIHR) Collaboration for Leadership in Applied Health Research and Care South West Peninsula (NIHR CLAHRC

For peer review only - http://bmjopen.bmj.com/site/about/guidelines.xhtml

South West Peninsula). The views expressed are those of the author(s) and not necessarily those of the NHS, the NIHR or the Department of Health and Social Care.

References

1. Tattersall RB. Mild familial diabetes with dominant inheritance. *Quarterly Journal of Medicine* 1974;43(170):339-57.
2. Murphy R, Ellard S, Hattersley AT. Clinical implications of a molecular genetic classification of monogenic beta-cell diabetes. *Nature Clinical Practice Endocrinology & Metabolism* 2008;4(4):200-13. doi: 10.1038/ncpendmet0778
3. Steele AM, Shields BM, Wensley KJ, et al. Prevalence of vascular complications among patients with glucokinase mutations and prolonged, mild hyperglycemia. *JAMA-Journal of the American Medical Association* 2014;311(3):279-86.
4. Stride A, Shields B, Gill-Carey O, et al. Cross-sectional and longitudinal studies suggest pharmacological treatment used in patients with glucokinase mutations does not alter glycaemia. *Diabetologia* 2014;57(1):54-56.
5. Pearson ER, Starkey BJ, Powell RJ, et al. Genetic cause of hyperglycaemia and response to treatment in diabetes. *Lancet* 2003;362:1275-81.
6. Thanabalasingham G, Owen KR. Diagnosis and management of maturity onset diabetes of the young (MODY). *British Medical Journal* 2011;343(d6044)
7. Shields BM, Hicks S, Shepherd MH, et al. Maturity-onset diabetes of the young (MODY): how many cases are we missing? *Diabetologia* 2010;53:2504-08.
8. Fajans SS, Bell GI, Bowden DW, et al. Maturity-onset diabetes of the young. *Life Sciences* 1994;55(6):413-22.
9. Shields BM, McDonald TJ, Ellard S, et al. The development and validation of a clinical prediction model to determine the probability of MODY in patients with young-onset diabetes. *Diabetologia* 2012 [published Online First: 5th January 2012]
10. Besser REJ, Shepherd MH, McDonald TJ, et al. Urinary c-peptide-to-creatinine ratio is a practical outpatient tool for identifying hepatocyte nuclear factor 1-α/hepatocyte nuclear factor 4-α maturity-onset diabetes of the young from long-duration type 1 diabetes. *Diabetes Care* 2011;34:1-6.
11. McDonald TJ, Colclough K, Brown R, et al. Islet autoantibodies can discriminate maturity-onset diabetes of the young (MODY) from type 1 diabetes. *Diabetic Medicine* 2011;28:1028-33.
12. Peters JL, Anderson R, Hyde C. Development of an economic evaluation of diagnostic strategies: the case of monogenic diabetes. *BMJ Open* 2013;3
13. Shields BM, Shepherd M, Hudson M, et al. Population-Based Assessment of a Biomarker-Based Screening Pathway to Aid Diagnosis of Monogenic Diabetes in Young-Onset Patients. *Diabetes Care* 2017;40(8):1017-25. doi: 10.2337/dc17-0224 [published Online First: 2017/07/14]
14. The EuroQol Group. EuroQol - a new facility for the measurement of health-related quality of life. *Health Policy* 1990;16(3):199-208.
15. Husereau D, Drummond M, Petrou S, et al. Consolidated Health Economic Evaluation Reporting Standards (CHEERS) statement. *Pharmacoeconomics* 2013;31(5):361-7. doi: 10.1007/s40273-013-0032-y [published Online First: 2013/03/27]
16. Palmer AJ, Roze S, Valentine WJ, et al. The CORE Diabetes Model: Projecting long-term clinical outcomes, costs and cost-effectiveness of interventions in diabetes mellitus (types 1 and 2) to support clinical and reimbursement decision-making. *Current Medical Research and Opinion* 2004;20:S5-S26. doi: 10.1185/030079904x1980

For peer review only - http://bmjopen.bmj.com/site/about/guidelines.xhtml

17. Office for National Statistics. Population Estimates by Ethnic Group 2002-2009. Statistical Bulletin, 2011.
18. Shepherd M, Shields B, Ellard S, et al. A genetic diagnosis of HNF1A diabetes alters treatment and improves glycaemic control in the majority of insulin-treated patients. *Diabetic Medicine* 2009;26:437-41.
19. Curtis LA, Burns A. Unit costs of health and social care 2017: Personal Social Services Research Unit, University of Kent, 2017.
20. National Institute for Health and Clinical Excellence. BNF, 2018.
21. Farmer AJ, Wade AN, French DP, et al. Blood glucose self-monitoring in type 2 diabetes: a randomised controlled trial. *Health Technol Assess* 2009;13(15):iii-iv, ix-xi, 1-50. doi: 10.3310/hta13150 [published Online First: 2009/03/04]
22. Yeaw J, Chan Lee W, Aagren M, et al. Cost of self-monitoring of blood glucose in the United States among patients on an insulin regimen for diabetes. *Journal of Managed Care Pharmacy* 2012;18(1):21-32.
23. Currie CJ, Gale EAM, Poole CD. Estimation of primary care treatment costs and treatment efficacy for people with type 1 and Type 2 diabetes in the United Kingdom from 1997 to 2007. *Diabetic Medicine* 2010;27(8):938-48.
24. National Institute for Health and Clinical Excellence. Guide to the methods of technology appraisal, 2008.
25. Naylor RN, John PM, Winn AN, et al. Cost-effectiveness of MODY genetic testing: translating genomic advances into practical health applications. *Diabetes Care* 2014;37:202-09.
26. Thanabalasingham G, Pal A, Selwood MP, et al. Systematic assessment of etiology in adults with a clinical diagnosis of young-onset type 2 diabetes is a successful strategy for identifying maturity-onset diabetes of the young. *Diabetes Care* 2012;35(6):1206-21.
27. Huang ES, Shook M, Jin L, et al. The impact of patient preferences on the cost-effectiveness of intensive glucose control in older patients with new-onset diabetes. *Diabetes Care* 2006;29(2):259-64.
28. Shepherd M, Hattersley AT. 'I don't feel like a diabetic any more': the impact of stopping insulin in patients with maturity onset diabetes of the young following genetic testing. *Clinical Medicine* 2004;4(2):144-47.
29. Shepherd M, Miles S, Jones J, et al. Differential diagnosis: Identifying people with monogenic diabetes. *Journal of Diabetes Nursing* 2010;14(9):342-47.

For peer review only

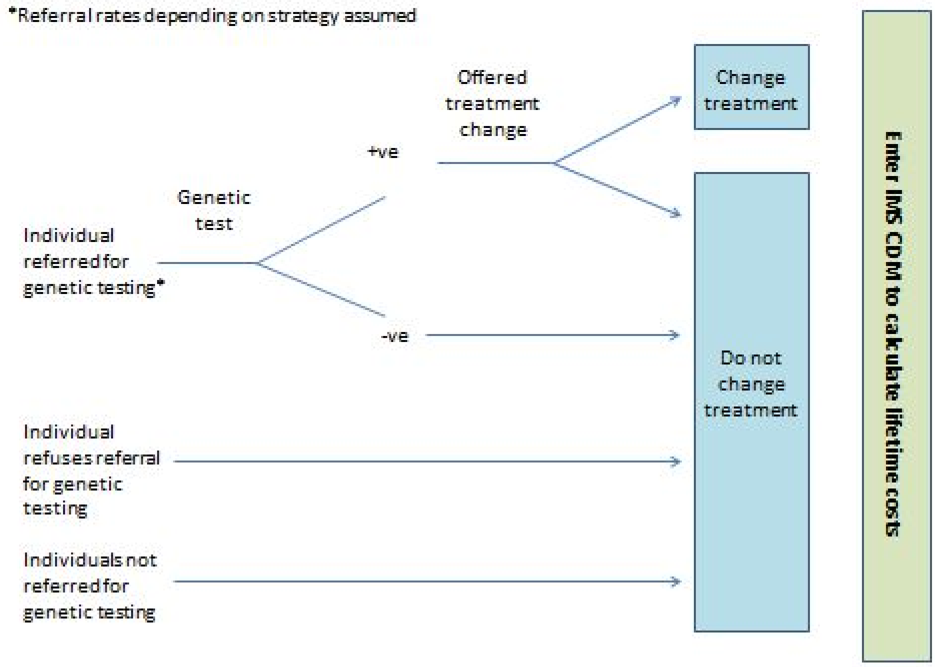

For peer review only

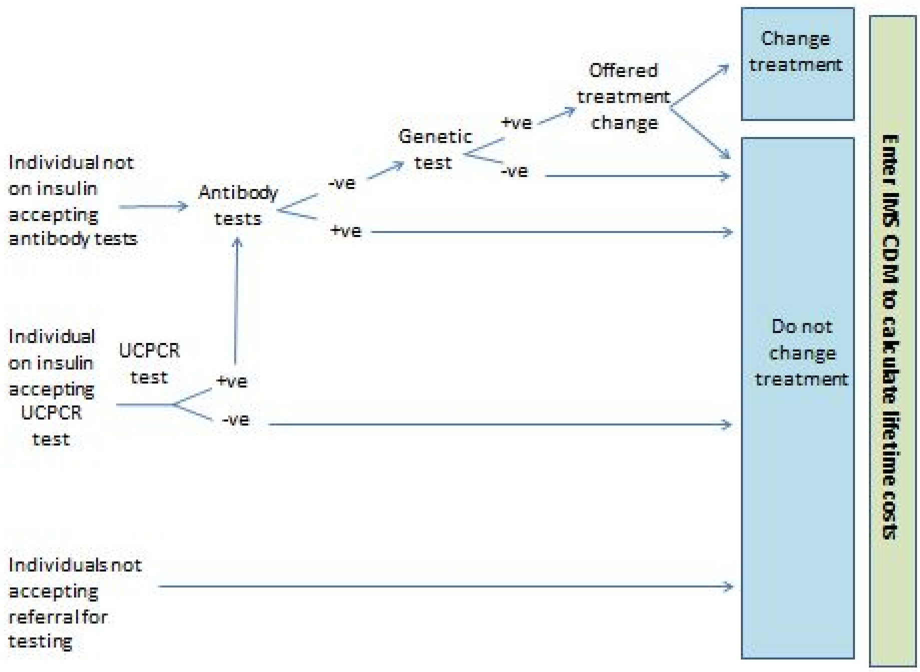

For peer review only

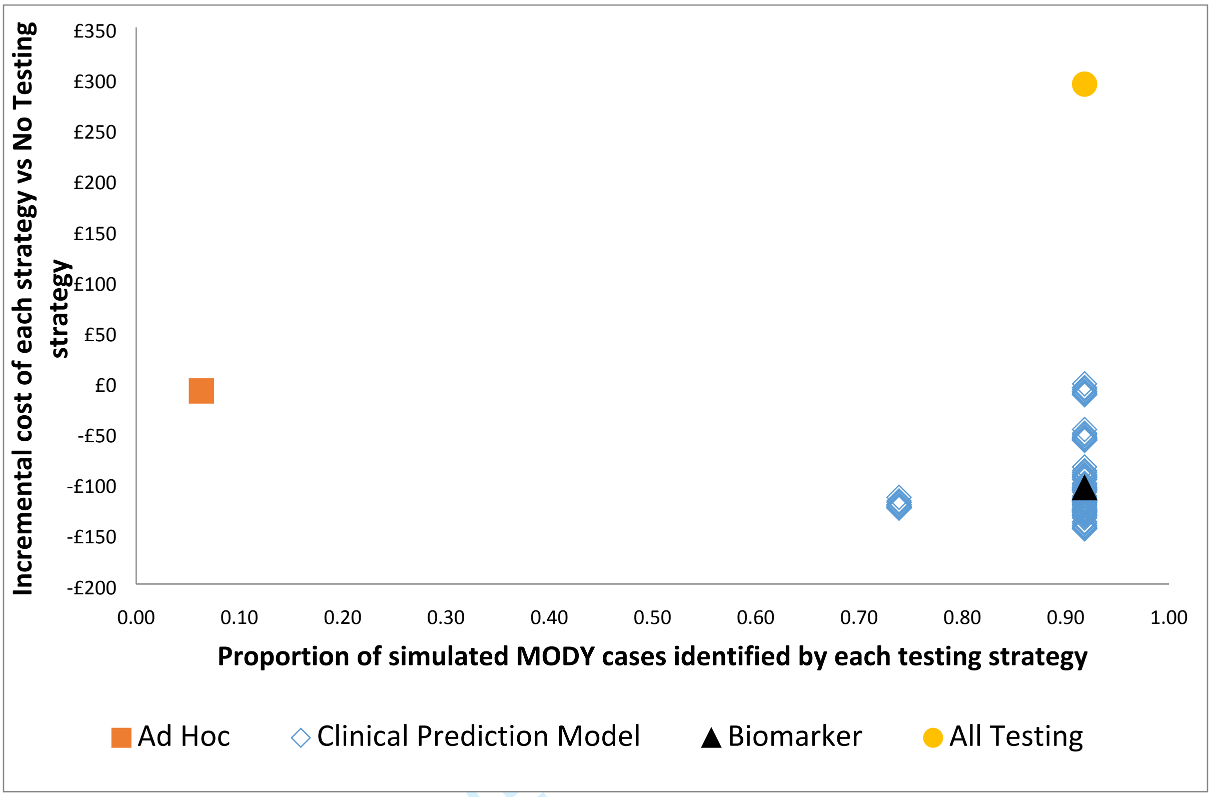

For peer review only

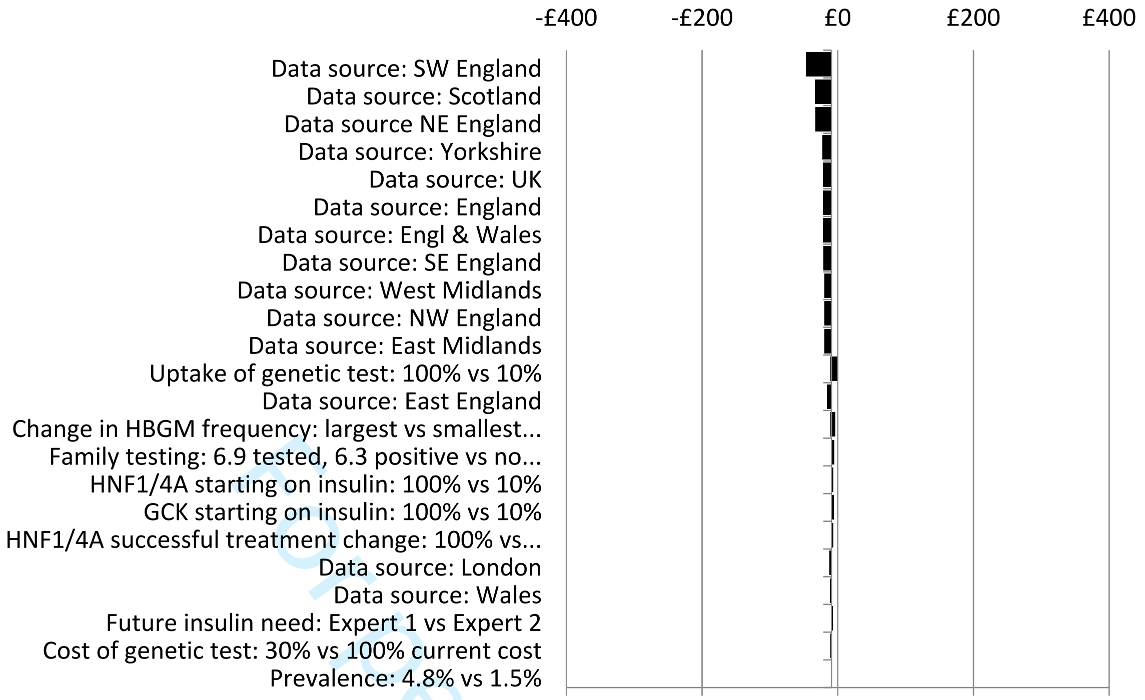

For peer review only

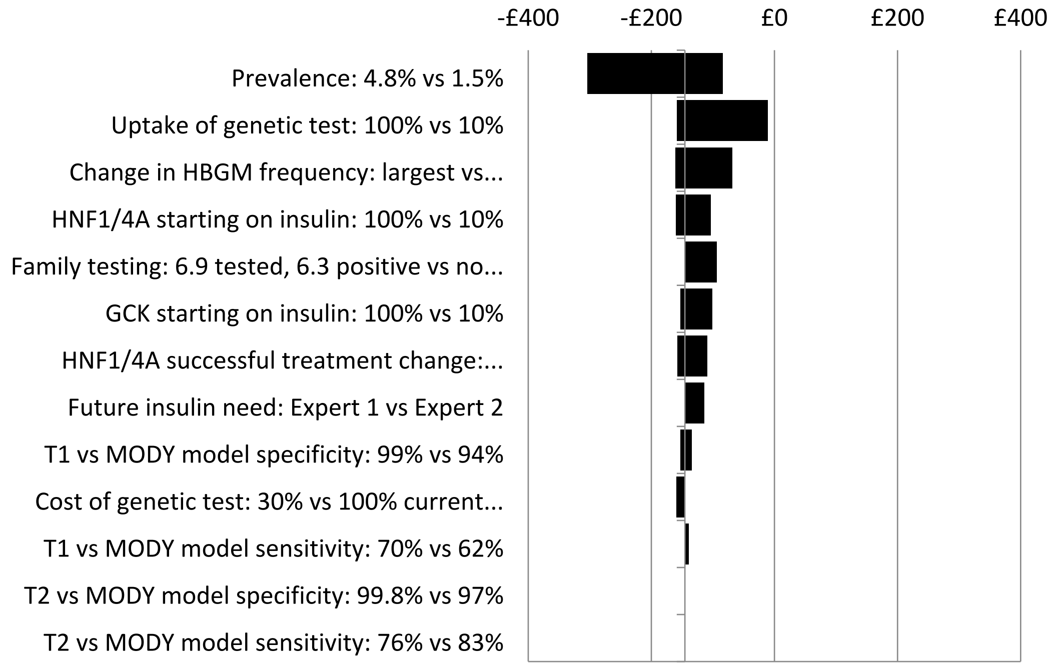

For peer review only

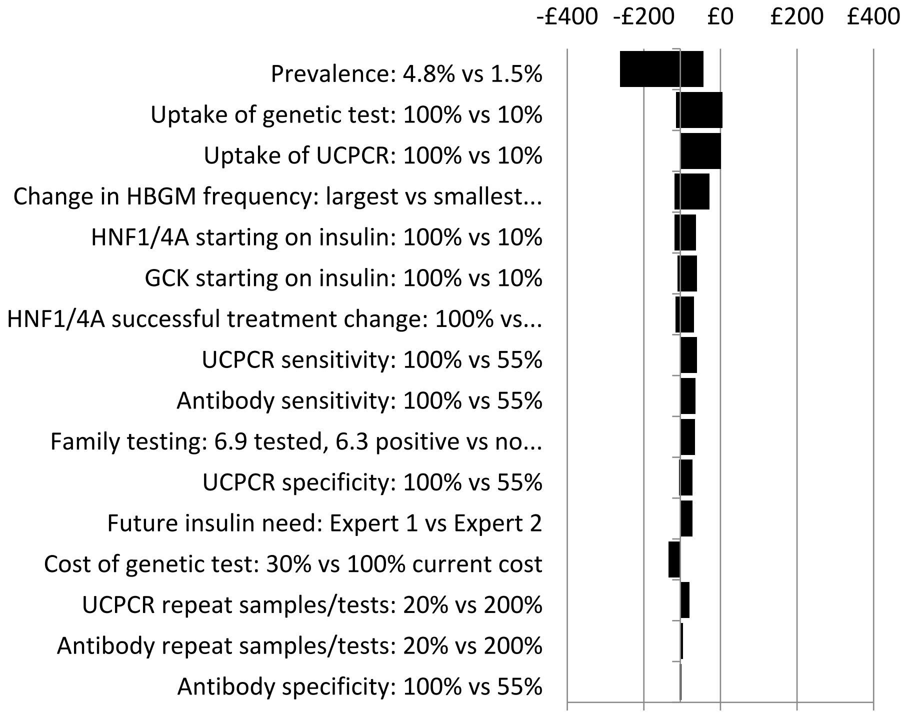

For peer review only

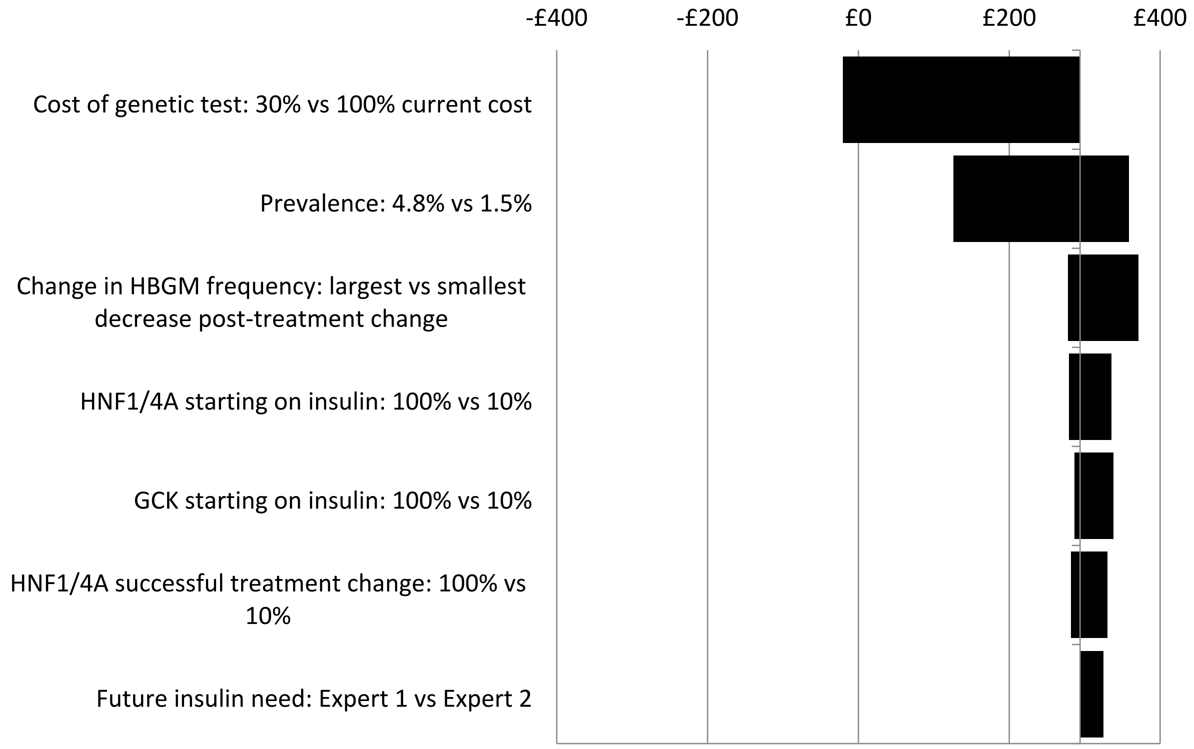
-£400
-£200
£0
£200
£400
Cost of genetic test: 30% vs 100% current cost
Prevalence: 4.8% vs 1.5%
Change in HBGM frequency: largest vs smallest decrease post-treatment change
HNF1/4A starting on insulin: 100% vs 10%
GCK starting on insulin: 100% vs 10%
HNF1/4A successful treatment change: 100% vs 10%
Future insulin need: Expert 1 vs Expert 2

Supplementary Data 1: Parameters and results for Cohort 2

Cohort 2 - Diagnosed with diabetes <30yrs old and still <30 yrs old at start of model

Table 1A Characteristics of the modelled Cohort 2 at entry to the model

Characteristic	Parameter value	Evidence source
Prevalence (95% confidence interval)		
GCK mutation	1·2% (0.5%, 2.3%)	Shields et al[1] & unpublished data from accompanying clinical study (N=687)
HNF1A mutation	0·9% (0.3%, 1.9%)	Shields et al[1] & unpublished data from accompanying clinical study (N=687)
HNF4A mutation	0.1% (0%, 0.5%)	Shields et al[1] & unpublished data from accompanying clinical study (N=687)
Type 1 diabetes[a]	93·4% (91.3%, 95.2%)	Unpublished data from accompanying clinical study (N=687)
Type 2 diabetes	4·5% (3.1%, 6.3%)	Unpublished data from accompanying clinical study (N=687)
Age (years) [b]	19	Unpublished data from accompanying clinical study (N=687)
Time since diagnosis (years) [b]	8	
Body mass index [b]	25·7	
HbA1c (mmol/mol) [b]	59.8	
Female	50%	
Systolic blood pressure [b]	131·7	[2]
Total cholesterol [b]	4·74	[2]
High density lipoprotein [b]	1·31	[2]
Low density lipoprotein [b]	2·61	[2]
Triglycerides [b]	0·83	[2]
Caucasian	89%	[3]

For peer review only

For peer review only - http://bmjopen.bmj.com/site/about/guidelines.xhtml

Black	4%	3
Asian	7%	3

[a] Defined as receiving insulin treatment within 12 months of diabetes diagnosis.
[b]Mean.

Table 1B Percentage (95% CI) of referred individuals tested for mutations in GCK and/or HNF1A and HNF4A genes by true diagnosis (from unpublished UK referral centre data)

True diabetes diagnosis	Percentage (95% CI) [N=1399]		
	***GCK* only**	***HNF1A* and *HNF4A***	***GCK*, *HNF1A* and *HNF4A***
Not monogenic	15.8% (13.4%, 18.4%)	69.0% (65.8%, 72.0%)	15.2% (12.9%, 17.8%)
GCK mutation	94.6% (91.0%, 97.1%)		5.3% (2.9%, 9.0%)
HNF1A mutation		95.0% (91.0%, 97.6%)	5.0% (2.4%, 9.0%)
HNF4A mutation		96.4% (89.8%, 99.2%)	3.6% (0.8%, 10.2%)

Table 1C Percentage (95% CI) of cohort not accepting offer of testing, or requiring multiple tests for the Biomarker Testing strategy

	Percentage (95% CI)	
Number of tests	UCPCR (including urine sample) N=1299	Autoantibody (including blood sample) N=419
0	12·8% (11.0%, 14.7%)	6·9% (4.7%, 9.8%)
1	84·6% (82.5%, 86.5%)	90·5% (87.2%, 93.1%)
2	2·4% (1.6%, 3.4%)	2·6% (1.3%, 4.6%)
3	0·1% (0.04%, 0.7%)	0%

UCPCR, urinary c-peptide creatinine ratio. Unpublished data from accompanying clinical study.

Table 1D Multipliers (and 95% confidence intervals) to inform cascade genetic testing of diabetic family members

**Number of relatives test per true monogenic diabetes case identified**	**Cohort 2 multiplier**	**Data source**
Relatives positive for monogenic diabetes	5.6 (4.7, 6.5)	Re-analysis of Shields et al[4] (specific to definition of modelled cohort)
Relatives negative for monogenic diabetes	0.6 (0.3, 1.0)	

For peer review only - http://bmjopen.bmj.com/site/about/guidelines.xhtml

For peer review only

Table 1E Pre-genetic treatment pattern, cost and frequency of HBGM by true diagnosis

	Treatment	% receiving treatment	Mean monthly treatment costs	Mean frequency of HBGM[a]
Type 1	Insulin only	100%	£52	78
Type 2	Insulin only	0%	£55	43
	Insulin + tablets	19%	£50	43
	Tablets only	68%	£2	17
	No diabetes treatment	13%	£0	0
GCK	Insulin only	75% (19%, 99%)	£5	52 (0, 110)
	Tablets only	25% (0.6%, 81%)	£1	0
HNF1A or HNF4A	Insulin only	67% (35%, 90%)	£18	63 (37, 90)
	Insulin + tablets	0%		
	Tablets	25.0% (6%, 57%)	£1	
	No diabetes treatment	8% (0.2%, 38%)	£0	0

[a]HBGM, home blood glucose monitoring

Table 1F Post-diagnosis HBGM frequency by treatment changed to and true diagnosis

	Time since diagnosis of monogenic diabetes			
	1 month	3 months	6 months	12 months
GCK – no diabetes treatment	0	0	0	0
HNF1A and HNF4A – tablets only	41 (19, 62)	23 (5, 41)	19 (6, 33)	16 (3, 28)

Table 1G Percentage of individuals with HNF1A or HNF4A mutations changing to more appropriate treatment after receiving a diagnosis of monogenic diabetes

	Time since treatment change (month)			
	1	3	6	12
Percentage changing to more appropriate treatment	100% (73%, 100%)	100% (73%, 100%)	100% (73%, 100%)	100% (73%, 100%)

For peer review only

For peer review only - http://bmjopen.bmj.com/site/about/guidelines.xhtml

Table 1H Summary of base case, sensitivity and threshold analyses

**Parameter**	**Base case justification**	**Justification of sensitivity/threshold analyses**
Long-term insulin need for individuals with HNF1A or HNF4A mutations	Expert 1 (see Supplementary Data 2)	Expert 2, who assumed greater insulin need sooner.
Prevalence of monogenic diabetes	In the accompanying clinical study, the total number of cases of monogenic diabetes was 14 from a total of 687 individuals screened. This leads to an estimated prevalence within the definition of Cohort 1 of 14/687 = 2% (see Table 1A above).	In sensitivity analyses it was assumed that:<br>1. all if the remaining 993 who were eligible to be screened in the accompanying clinical study would fit the definition for Cohort 2, but were not cases of monogenic diabetes, therefore a lower prevalence of monogenic diabetes was assumed (14/1670 = 0.8%).<br>2. as an upper limit, the prevalence of monogenic diabetes was doubled (28/687 = 4%).
Sensitivity and specificity of the Ad Hoc Testing strategy	Based on referral rate data for Northern Ireland (the region with the lowest referral rates)[4]	Analysed all regions using estimates of sensitivity and specificity given in Supplementary Data 3.
Genetic test cost	UK referral centre costs[5]: £350 for GCK mutation; £450 for HNF1A and HNF4A mutations.	Threshold analyses to identify at what cost of the GCK and HNF1A and HNF4A genetic tests would the All Tested strategy incur no additional costs over the No Testing strategy. Costs of tests for GCK and HNF1A and HNF4A mutations were reduced in 10% steps to just 10% of their base case costs: £35 for GCK and £45 for HNF1A and HNF4A.
Uptake of UCPCR test	Based on data from the accompanying clinical study which investigated the application of the Biomarker Testing strategy.<br>Uptake of UCPCR was assumed to be 87% (see Table 1C above).	Threshold analyses where UCPCR test uptake was assumed to range from 100% to just 10%.<br>It was hypothesised that test uptake in practice is likely to be lower than test uptake in the accompanying clinical study where individuals have consented to participating in a study.
Uptake of autoantibody test	Based on data from the accompanying clinical study which investigated the application of the Biomarker Testing strategy.<br>Uptake of autoantibody testing was assumed to be 93% (see Table 1C above).	Threshold analyses where autoantibody test uptake was assumed to range from 100% to just 10%.<br>It was hypothesised that test uptake in practice is likely to be lower than test uptake in the accompanying clinical study where individuals have consented to participating in a study
Uptake of genetic test	Based on data from the accompanying clinical study which investigated the application of the Biomarker Testing strategy.<br>Uptake of genetic testing was assumed to be the same as for autoantibody testing (93%) since the same blood sample for autoantibody testing was used for the genetic testing (see Table 1C above).	Threshold analyses where genetic test uptake was assumed to range from 100% to just 10%.<br>It was hypothesised that test uptake in practice is likely to be lower than test uptake in the accompanying clinical study where individuals have consented to participating in a study

For peer review only - http://bmjopen.bmj.com/site/about/guidelines.xhtml

Repeat urine samples and UCPCR tests	Based on data from the accompanying clinical study which investigated the application of the Biomarker Testing strategy. The percentage of repeat urine samples and UCPCR tests was assumed to be 3% (see Table 1C above).	Threshold analyses were undertaken assuming no repeats, 1%, 5%, 10%, 20%, 50%, 100%, 150% and 200% of samples and tests needed to be repeated. 200% repeat samples and tests can be interpreted as every individual requiring another 2 urine samples and UCPCR tests to be done, so that in total every individual has provided 3 urine samples and 3 UCPCR tests have been done – an extreme assumption.
Repeat blood samples and autoantibody tests	Based on data from the accompanying clinical study which investigated the application of the Biomarker Testing strategy. The percentage of repeat blood samples and autoantibody tests was assumed to be 3% (see Table 1C above).	Threshold analyses were undertaken assuming no repeats, 1%, 5%, 10%, 20%, 50%, 100%, 150% and 200% of samples and tests needed to be repeated. 200% repeat samples and tests can be interpreted as every individual requiring another 2 blood samples and autoantibody tests to be done, so that in total every individual has provided 3 blood samples and 3 autoantibody tests have been done – an extreme assumption.
Sensitivity of UCPCR test	Based on data from Besser et al[6] which used a prevalent case-control diagnostic study design: 0.94 (see Supplementary Data 3).	Since the sensitivity estimate for the UCPCR test is from a case-control diagnostic study, it is likely that the reported estimate will be greater than in practice. Threshold analyses have therefore been undertaken to investigate the impact of assuming lower sensitivity values in particular.<br><br>Threshold analyses assumed sensitivity estimates between 1 and 0·55.
Specificity of UCPCR test	Based on data from Besser et al[6] which used a prevalent case-control diagnostic study design: 0.96 (see Supplementary Data 3).	Since the specificity estimate for the UCPCR test is from a case-control diagnostic study, it is likely that the reported estimate will be greater than in practice. Threshold analyses have therefore been undertaken to investigate the impact of assuming lower specificity values in particular.<br><br>Threshold analyses assumed specificity estimates between 1 and 0·55.
Sensitivity of autoantibody test	Based on data from MacDonald et al[7] which used a prevalent case-control diagnostic study design: 0.99 (see Supplementary Data 3).	Since the sensitivity estimate for the autoantibody test is from a case-control diagnostic study, it is likely that the reported estimate will be greater than in practice. Threshold analyses have therefore been undertaken to investigate the impact of assuming lower sensitivity values in particular.<br><br>Threshold analyses assumed sensitivity estimates between 1 and 0·55.
Specificity of autoantibody test	Based on data from MacDonald et al[7] which used a prevalent case-control diagnostic study design: 0.82 (see Supplementary Data 3).	Since the specificity estimate for the autoantibody test is from a case-control diagnostic study, it is likely that the reported estimate will be greater than in practice. Threshold analyses have therefore been undertaken to investigate the impact of assuming different specificity values.<br><br>Threshold analyses assumed specificity estimates between 1 and 0·55.
Percentage of individuals with GCK mutation	Based on data from the accompanying clinical study which investigated the	Threshold analyses assuming 100% to 10% (in 10% decrements) of individuals with GCK mutations are receiving insulin at the start of the model.

For peer review only

who are receiving insulin treatment at the start of the model	application of the Biomarker Testing strategy. 75% of individuals with GCK mutation are receiving insulin treatment at the start of the model, while 25% are receiving tablets (metformin and sulphonylureas). See **Error! Reference source not found.** above.	
Percentage of individuals with HNF1A or HNF4A mutation who are receiving insulin treatment at the start of the model	Based on data from the accompanying clinical study which investigated the application of the Biomarker Testing strategy. 67% of individuals with HNF1A or HNF4A mutation are receiving insulin treatment at the start of the model, 25% are receiving tablets (metformin and sulphonylureas) and 8% are not treated pharmacologically. See **Error! Reference source not found.** above.	Threshold analyses assuming 100% to 10% (in 10% decrements) of individuals with HNF1A or HNF4A mutations are receiving insulin at the start of the model.
Percentage of individuals with HNF1A or HNF4A mutations who remain on most appropriate treatment after a diagnosis of monogenic diabetes	Based on data from the accompanying clinical study which investigated the application of the Biomarker Testing strategy. At every follow-up point after treatment change, 100% of individuals with HNF1A or HNF4A mutations remained on the most appropriate treatment (see **Error! Reference source not found.** above).	The base case estimates are based on a small number of participants. Threshold analyses have been conducted to investigate the percentage of individuals with HNF1A or HNF4A mutations who need to remain on tablets for the strategies to be cost-saving compared to No Testing.<br><br>It was assume that for all follow-up time periods after a monogenic diabetes diagnosis, the percentage receiving tablets is: 86%, 77%, 50%, 25% or 10%.
Cascade family testing	Analysis of referral rate data[4] indicate that for every 10 case of monogenic diabetes identified, 6.2 family members are also genetically tested: with 5.6 being positive for monogenic diabetes and 0.6 being negative for monogenic diabetes (see **Error! Reference source not found.**).	The impact of family cascade testing in the Ad Hoc Testing, Clinical Prediction Model Testing and Biomarker Testing strategies was investigated by removing all cascade family testing from the strategies.<br><br>Estimates of the magnitude of cascade family testing based on the 95% confidence interval limits are used to investigate the impact of this parameter: 4.7 to 6.5 family members who are found to be positive for monogenic diabetes, and 0. 3 to 1 family members who are found to be negative for monogenic diabetes.
Frequency of HBGM before and after changing treatment due to a diagnosis of monogenic diabetes	Based on data from the accompanying clinical study which investigated the application of the Biomarker Testing strategy. Data suggested that individuals with GCK mutations stopped HBGM after their diagnosis of monogenic diabetes, while individuals with HNF1A or HNF4A mutations	The 95% confidence limits for the estimated frequency of HBGM at the start of the model and at follow-up after a treatment change for individuals with HNF1A or HNF4A mutations were used in sensitivity analyses. The change in frequency of HBGM before and after a diagnosis of monogenic diabetes was maximised (which would favour strategies to identify cases of monogenic diabetes) by assuming the upper 95% confidence limit at baseline and the lower 95% confidence limits at follow-up. Conversely, the change in frequency of HBGM was

For peer review only - http://bmjopen.bmj.com/site/about/guidelines.xhtml

	significantly reduced their frequency of HBGM after a diagnosis of monogenic diabetes.	minimised (which would not be as favourable to strategies to identify cases of monogenic diabetes) by assuming the lower 95% confidence limit at baseline and the upper 95% confidence limit at follow-up.

Table 1I Summary of “base case” results

**Strategy**	**Total undiscounted LYs**	**Total discounted QALYs**	**Total discounted costs[a]**	**Incremental costs vs No Testing strategy[a]**	**% who are genetically tested**	
					**With monogenic diabetes**	**Without monogenic diabetes**
Clinical Prediction Model[b]	38·4	11·9	£54,000	-£100	93	3
Biomarker			£54,000	-£100	93	5
Ad Hoc			£54,100	0	7	<1
No Testing			£54,100	NA	0	0
All Testing			£54,400	£300	93	93

[a] rounded to nearest £100; [b] thresholds chosen to maximise costs saved

Fig 1A Incremental costs (vs No Testing) and the proportion of monogenic diabetes cases identified for each strategy

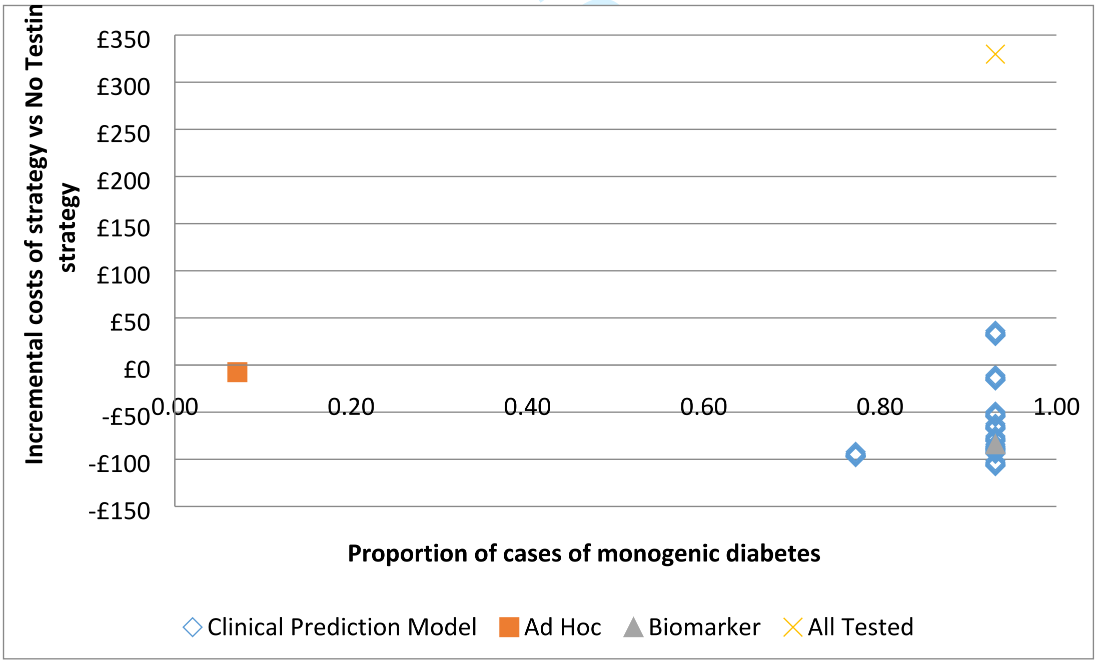

For peer re

Fig 1B Tornado plot of sensitivity analyses for the Ad Hoc Testing strategy

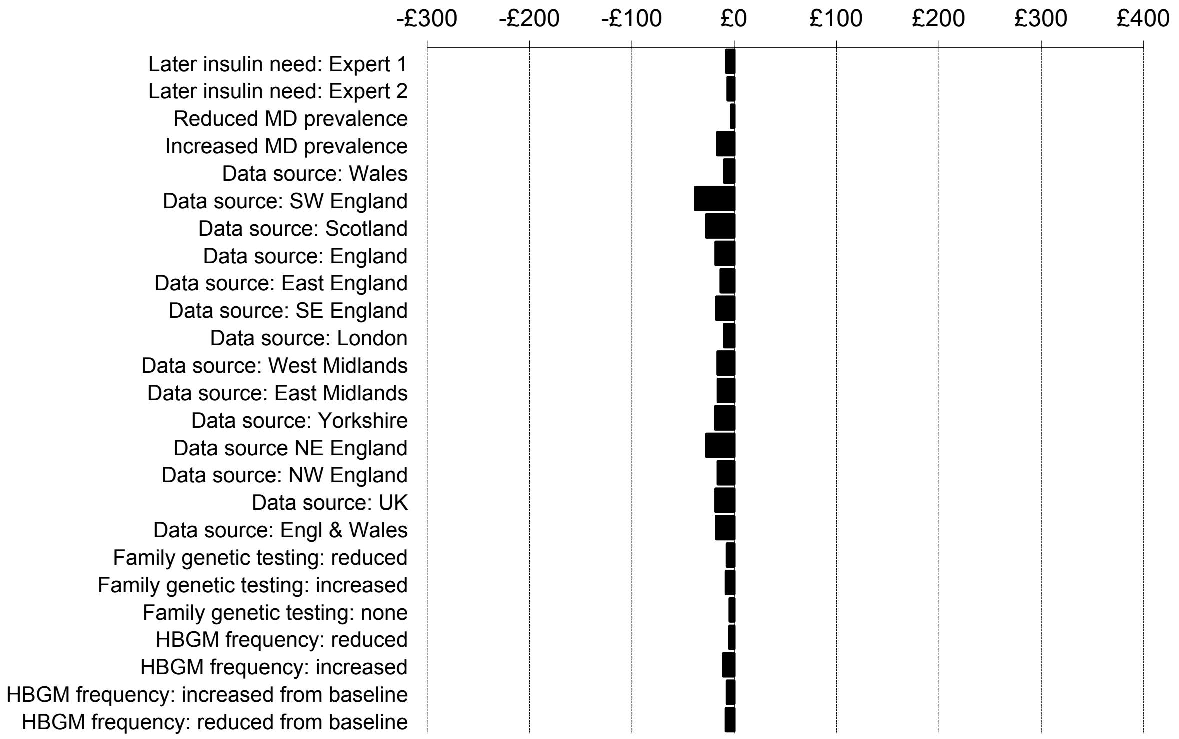

Fig 1C Tornado plot for the Clinical Prediction Model Testing strategy

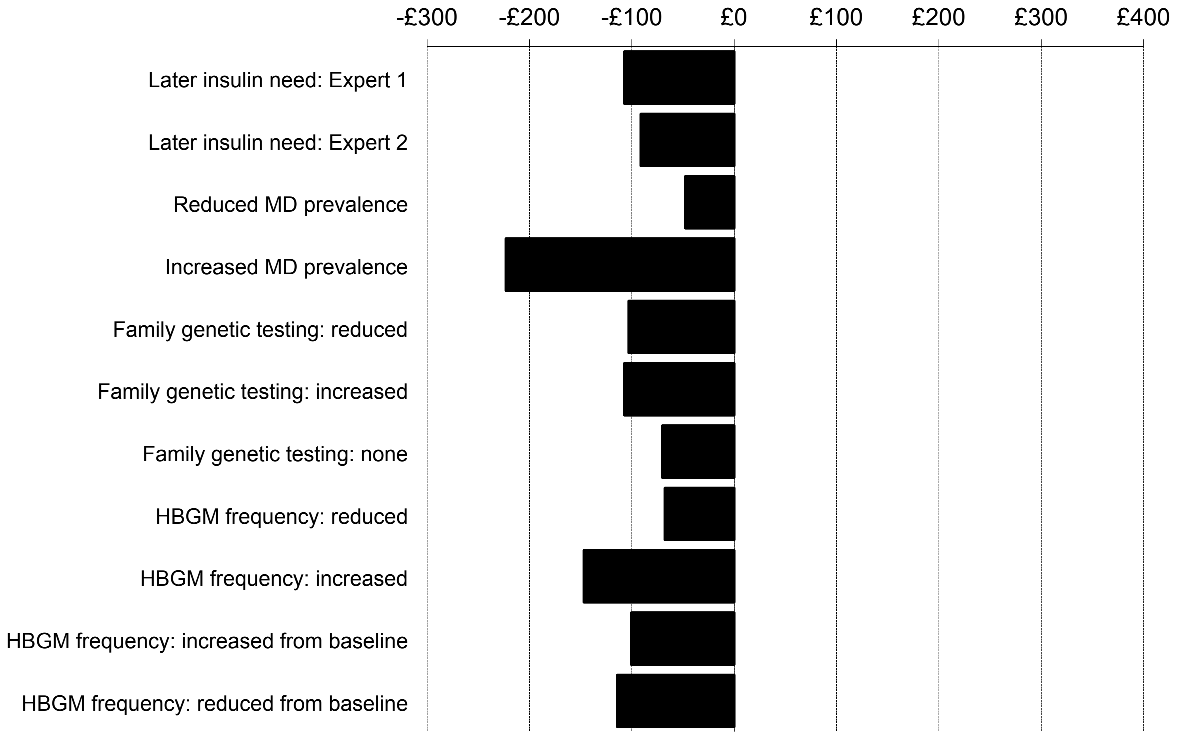

For peer review only - http://bmjopen.bmj.com/site/about/guidelines.xhtml

Fig 1D Tornado plot for the Biomarker Testing strategy

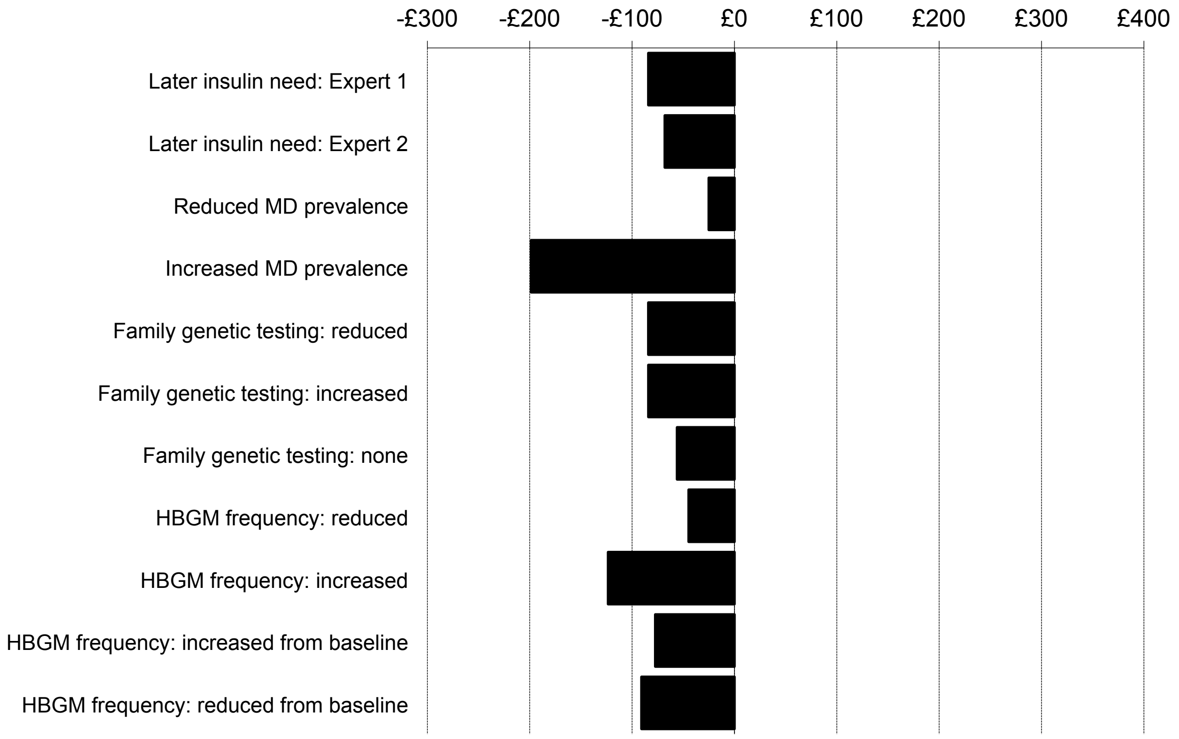

Fig 1E Tornado plot for the All Testing strategy

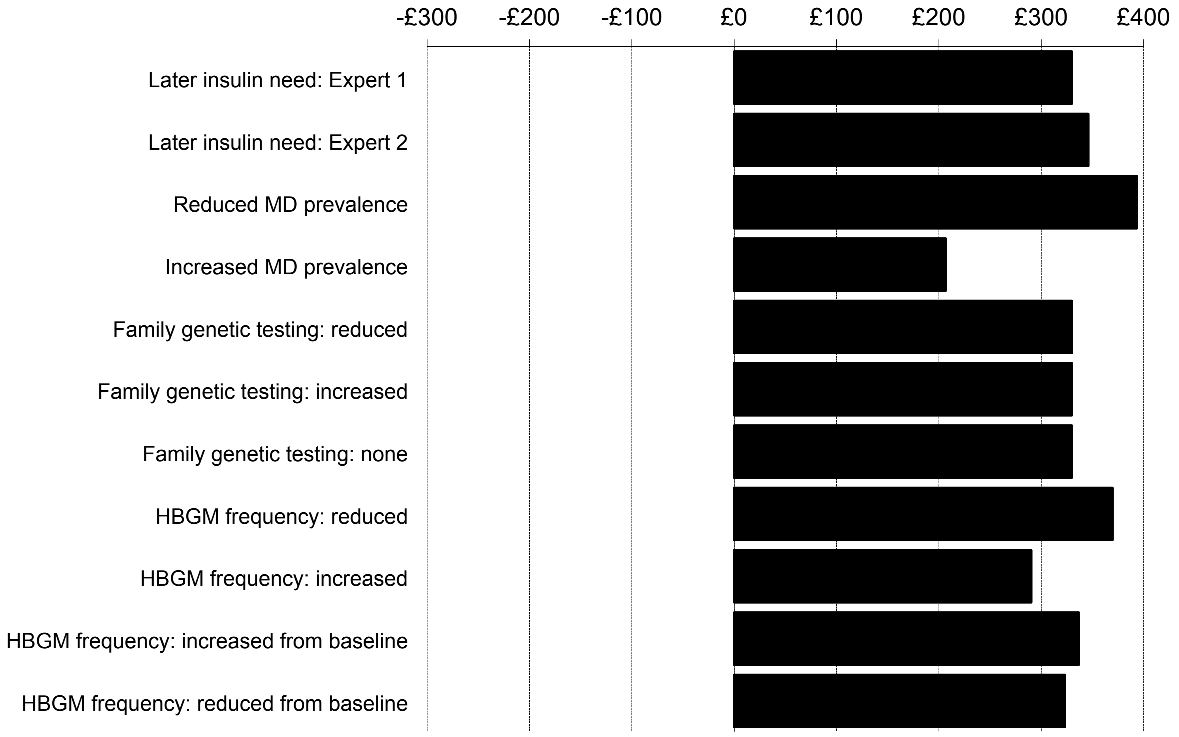

For peer review only - http://bmjopen.bmj.com/site/about/guidelines.xhtml

Fig 1F Incremental costs (vs No Testing) for all strategies for reducing percentage of GCK cohort starting on insulin

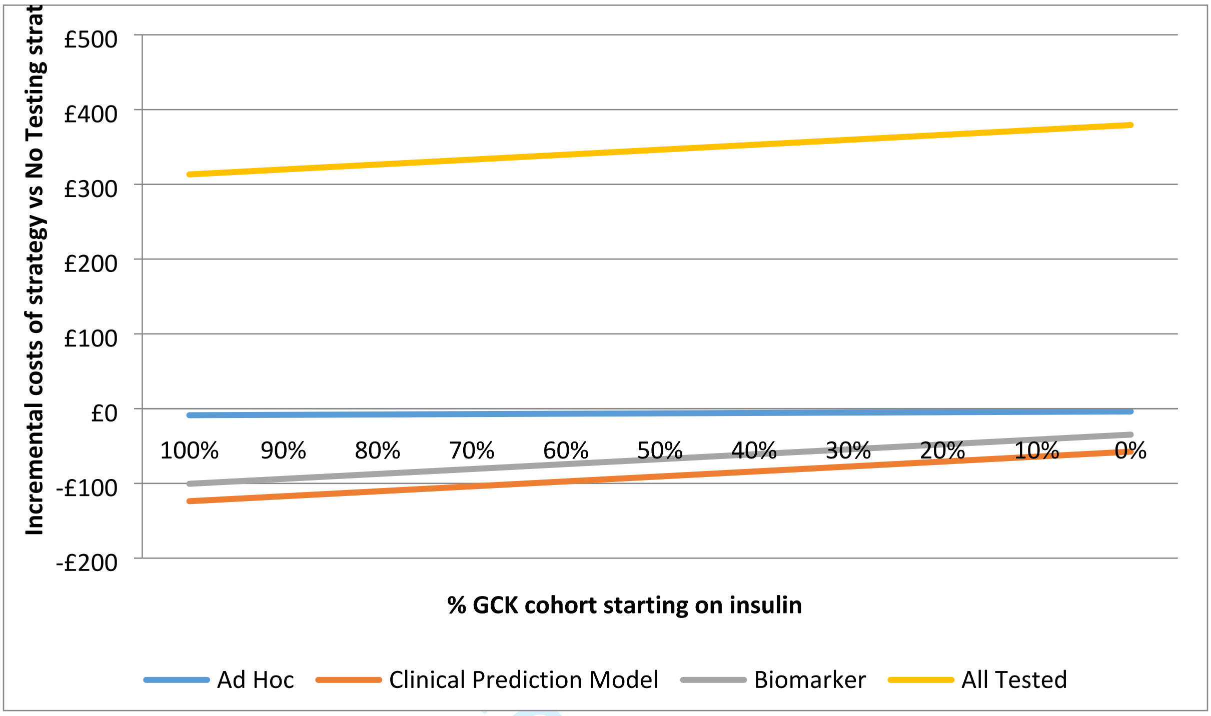

Fig 1G Incremental costs (vs No Testing) for all strategies for reducing percentage of HNF1A and HNF4A cohort starting on insulin

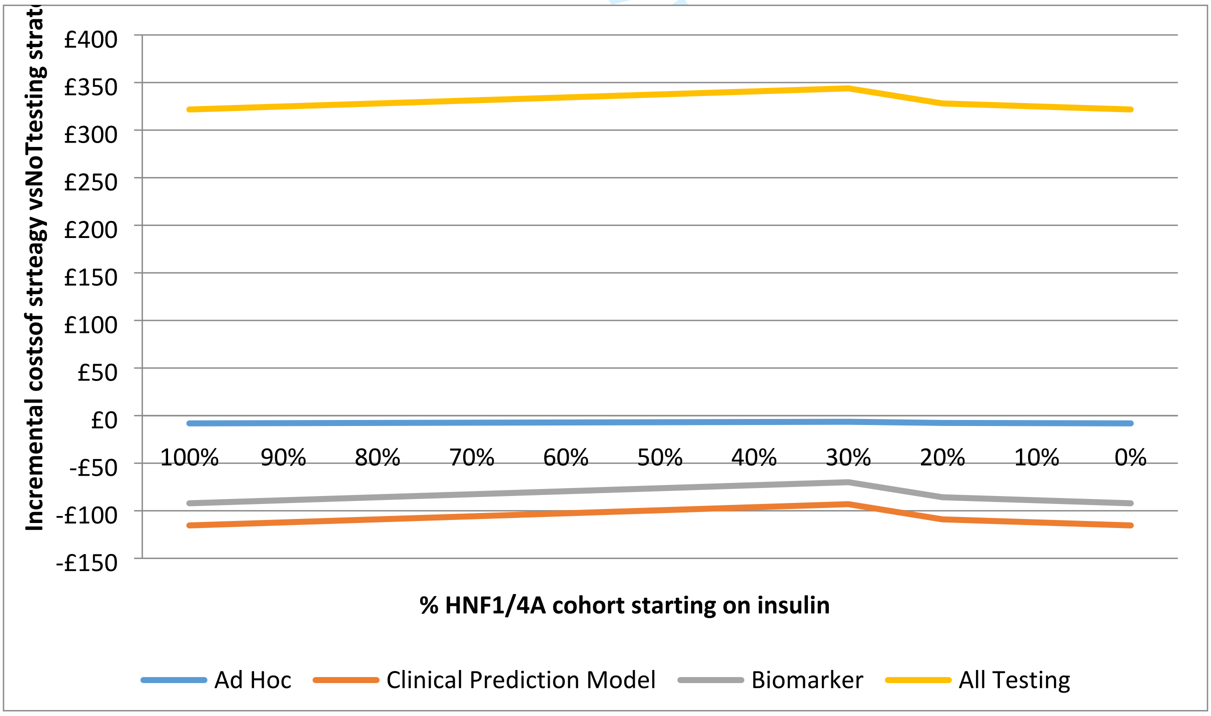

For peer review only - http://bmjopen.bmj.com/site/about/guidelines.xhtml

Fig 1H Incremental costs (vs No Testing) for the Biomarker Testing strategy with reducing levels of UCPCR and antibody testing uptake

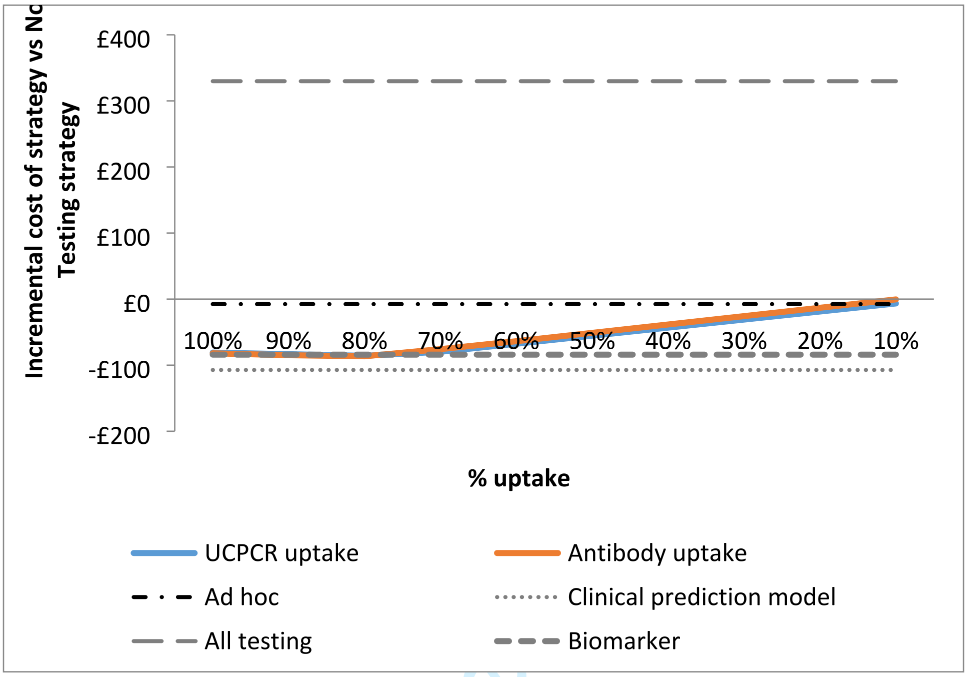

Fig 1I Incremental costs (vs No Testing) for the Biomarker Testing strategy with reducing estimates of sensitivity and specificity for the UCOCR and antibody tests

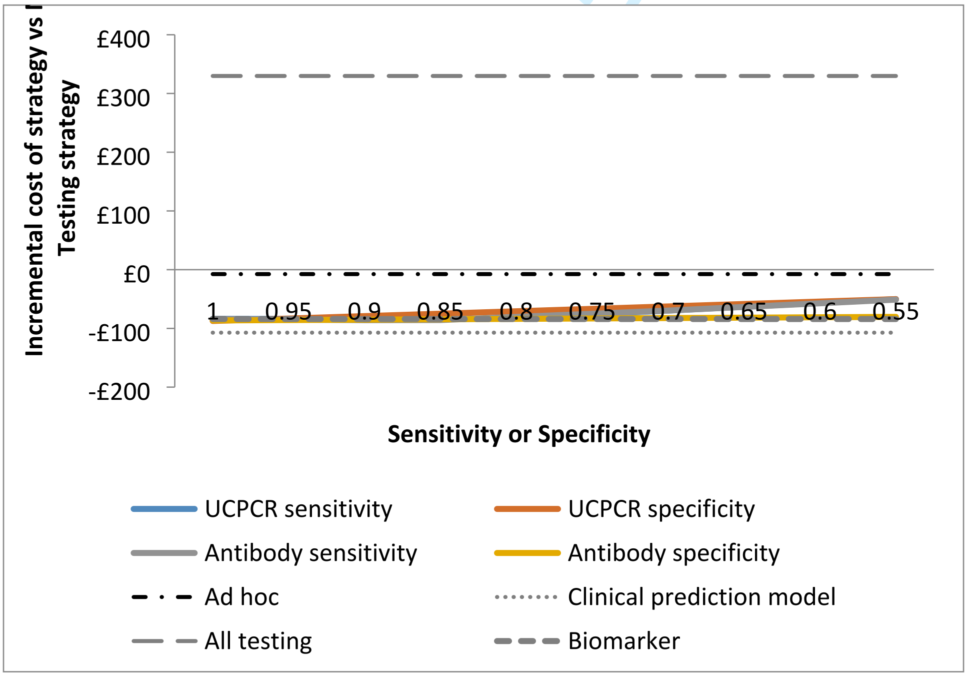

For peer review only - http://bmjopen.bmj.com/site/about/guidelines.xhtml

Fig 1J Incremental costs for the Biomarker Testing strategy (vs No Testing) with increasing estimates of repeat samples and UCPCR and autoantibody tests

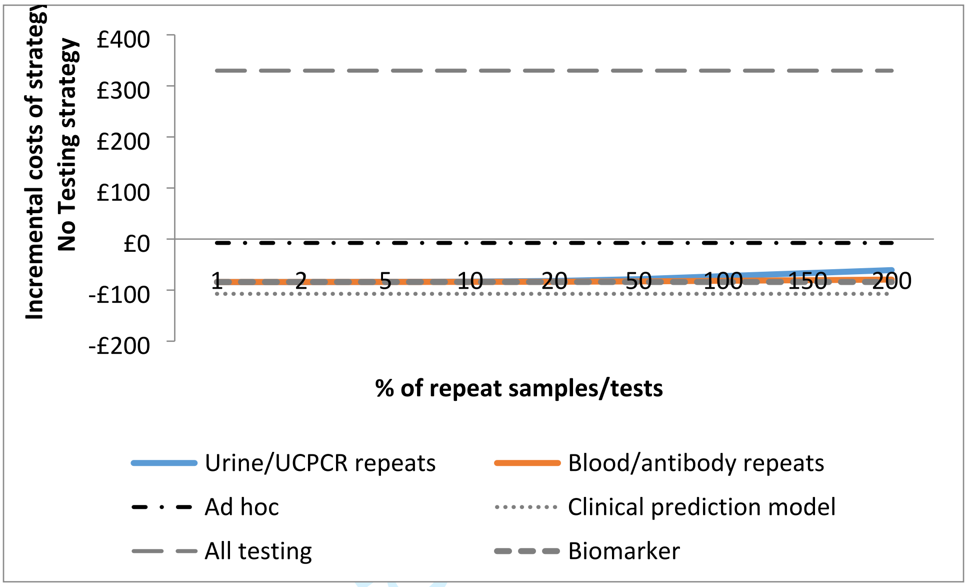

Fig 1K Incremental costs (vs No Testing) for all strategies when genetic test costs are reduced

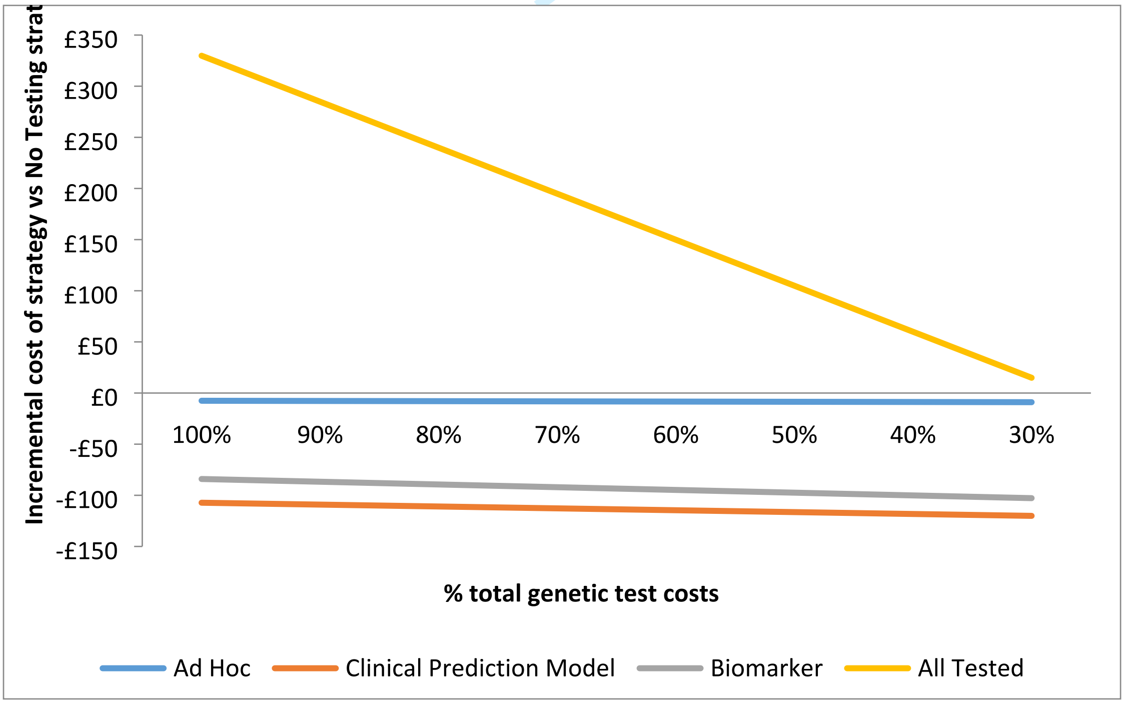

For peer review only - http://bmjopen.bmj.com/site/about/guidelines.xhtml

References

1. Shields BM, Shepherd M, Hudson M, et al. Population-Based Assessment of a Biomarker-Based Screening Pathway to Aid Diagnosis of Monogenic Diabetes in Young-Onset Patients. Diabetes Care 2017;40(8):1017-25. doi: 10.2337/dc17-0224 [published Online First: 2017/07/14]
2. Llaurado G, Gonzalez-Clemente J-M, Maymo-Masip E, et al. Serum levels of TWEAK and scavenger receptor CD163 in type 1 diabetes mellitus: relationship with cardiovascular risk factors. A case-control study. PLoS One 2012;7(8):e43919.
3. Office for National Statistics. Population Estimates by Ethnic Group 2002-2009. Statistical Bulletin, 2011.
4. Shields BM, Hicks S, Shepherd MH, et al. Maturity-onset diabetes of the young (MODY): how many cases are we missing? Diabetologia 2010;53:2504-08.
5. Royal Devon and Exeter NHS Foundation Trust. [Available from: http://www.rdehospital.nhs.uk/prof/molecular_genetics/tests/Full_Test_List.htm accessed 13th October 2014.
6. Besser REJ, Shepherd MH, McDonald TJ, et al. Urinary c-peptide-to-creatinine ratio is a practical outpatient tool for identifying hepatocyte nuclear factor 1-α/hepatocyte nuclear factor 4-α maturity-onset diabetes of the young from long-duration type 1 diabetes. Diabetes Care 2011;34:1-6.
7. McDonald TJ, Colclough K, Brown R, et al. Islet autoantibodies can discriminate maturity-onset diabetes of the young (MODY) from type 1 diabetes. Diabetic Medicine 2011;28:1028-33.

For peer review only

For peer review only - http://bmjopen.bmj.com/site/about/guidelines.xhtml

Supplementary Data 2: Parameters and results for Cohort 1

Cohort 1 - Diagnosed with diabetes <30yrs old and still <50 yrs old at start of model

Table 2A Characteristics of the modelled cohorts 1 and 2 at entry to the model

Characteristic	Parameter value	Evidence source
Prevalence (95% confidence interval)		
GCK mutation	0·7%<br>(0.4%, 1.4%)	Shields et al[1] & unpublished data from accompanying clinical study (N=1407)
HNF1A mutation	1·5%<br>(1.2%, 2.7%)	Shields et al[1] & unpublished data from accompanying clinical study (N=1407)
HNF4A mutation	0·2%<br>(0.1%, 0.6%)	Shields et al[1] & unpublished data from accompanying clinical study (N=1407)
Type 1 diabetes[a]	88·6%<br>(86.4%, 89.9%)	Unpublished data from accompanying clinical study (N=1407)
Type 2 diabetes	9.0%<br>(7.4%, 10.5%)	Unpublished data from accompanying clinical study (N=1407)
Age (years) [b]	25	Unpublished data from accompanying clinical study (N=1407)
Time since diagnosis (years) [b]	12	
Body mass index [b]	24·4	
HbA1c (mmol/mol) [b]	64.2	
Female (%)	50	
Systolic blood pressure [b]	131·7	[2]
Total cholesterol [b]	4·74	[2]
High density lipoprotein [b]	1·31	[2]
Low density lipoprotein [b]	2·61	[2]
Triglycerides [b]	0·83	[2]
Caucasian	89%	[3]

For peer review only

For peer review only - http://bmjopen.bmj.com/site/about/guidelines.xhtml

Black	4%	[3]
Asian	7%	[3]

[a] Defined as receiving insulin treatment within 12 months of diabetes diagnosis.
[b]Mean.

Table 2B Percentage (95% CI) of referred individuals tested for mutations in *GCK* and/or *HNF1A* and *HNF4A* genes by true diagnosis (from unpublished UK referral centre data)

True diabetes diagnosis	Percentage (95% CI) [N=2294]		
	***GCK* only**	***HNF1A* and *HNF4A***	***GCK*, *HNF1A* and *HNF4A***
Not monogenic	14.1% (12.3%, 16.0%)	70.0% (67.5%, 72.4%)	15.9% (14.0%, 18.0%)
GCK mutation	95.2% (92.3%, 97.3%)		4.8% (2.7%, 7.7%)
HNF1A mutation		96.2% (94.0%, 97.8%)	3.5% (2.0%, 5.7%)
HNF4A mutation		97.3% (93.2%, 99.2%)	2.7% (0.7%, 6.8%)

Table 2C Percentage (95% CI) of cohort not accepting offer of testing, or requiring multiple tests for the Biomarker Testing strategy

	Cohort 1	
Number of tests	UCPCR (including urine sample) N=2017	Autoantibody (including blood sample) N=624
0	11·9% (10.6%, 13.4%)	8·2% (6.1%, 10.6%)
1	86·1% (84.5%, 87.6%)	90·0% (87.4%, 92.3%)
2	1·8% (1.3%, 2.5%)	1·8% (0.9%, 3.1%)
3	0·1% (0.03%, 0.4%)	0%

UCPCR, urinary c-peptide creatinine ratio. Unpublished data from accompanying clinical study.

Table 2D Multipliers (and 95% confidence intervals) to inform cascade genetic testing of diabetic family members

**Number of relatives test per true monogenic diabetes case identified**	Multipliers (and 95% CIs)	**Data source**
Relatives positive for monogenic diabetes	5.9 (5.4, 6.3)	Re-analysis of Shields et al[4] (specific to definition of modelled cohort)
Relatives negative for monogenic diabetes	0.4 (0.2, 0.6)	

For peer review only - http://bmjopen.bmj.com/site/about/guidelines.xhtml

Table 2E Pre-genetic test treatment pattern, cost and frequency of HBGM by true diagnosis

Diabetes type	Treatment	% receiving treatment	Mean monthly treatment costs	Mean frequency of HBGM[a]
Type 1	Insulin only	100%	£52	78
Type 2	Insulin only	36%	£55	43
	Insulin + tablets	54%	£50	43
	Tablets only	3%	£2	17
	No diabetes treatment	7%	£0	0
GCK mutation	Insulin only	87.5% (47.3%, 99.7%)	£10	63 (19, 107)
	Tablets only	12.5% (0.3%, 52.6%)	£1	0
HNF1A and *HNF4A* mutation	Insulin only	78.4% (61.8%, 90.2%)	£23	
	Insulin + tablets	13.5% (4.5%, 28.8%)	£16	76 (52, 99)
	Tablets	5.4% (0.1%, 18.2%)	£2	
	No diabetes treatment	2.7% (0.1%, 14.2%)	£0	0

[a] HBGM, home blood glucose monitoring

Table 2F Estimated dose and timing of future insulin requirements for individuals identified as having *HNF1A* or *HNF4A* mutations

Population	Expert 1		Expert 2	
	**Years after start of model**	**Insulin need (u)**	**Years after start of model**	**Insulin need (U/kg)**
Tablets only	0-19<br>20-24<br>25-29<br>≥30 yrs	As at model start<br>10 + tablets<br>20+ tablets<br>30 + tablets	0-9<br>10-14<br>15-24<br>≥2 yrs	As at model start<br>0.25 + tablets<br>0.4 + tablets<br>0.5 (no tablets)
Tablets and insulin	0-4<br>5-14<br>≥15 yrs	As at model start<br>20 + tablets<br>30 + tablets	0-9<br>10-14<br>≥15 yrs	As at start of model<br>0.4 + tablets<br>0.5 (no tablets)
Insulin only	0-9<br>10-24<br>≥25 yrs	As at model start<br>50<br>60	≥0 yrs	0.5

Table 2G Post-diagnosis HBGM frequency (95%CI) by treatment changed to and true diagnosis

	Time since diagnosis of monogenic diabetes (months)			
**Mutation - Treatment received**	**1**	**3 months**	**6 months**	**12 months**
GCK mutation – no diabetes treatment	0	0	0	0
HNF1/4A mutation – tablets only	50 (27, 73)	36 (14, 57)	22 (11, 33)	21 (10, 32)
HNF1/4A mutation – insulin and tablets	89 (56, 121)	66 (44, 87)	70 (46, 93)	43 (25, 60)

For peer review only

Table 2H Justification of parameter values and variations used in base case and sensitivity analyses

Parameter	Base case justification	Justification of sensitivity/threshold analyses
Prevalence of monogenic diabetes	In the accompanying clinical study, the total number of cases of monogenic diabetes was 34 from a total of 1407 individuals screened. This leads to an estimated prevalence within the definition of Cohort 1 of 34/1407 = 2·4% (see **Error! Reference source not found.** above).	Although the total screened population was 1407 in the accompanying clinical study[1], the total eligible population in the defined geographical area was 2288. We could therefore assume:<br>1. that no more cases would have been found in the remaining eligible population not screened, i.e. the remaining 881 were not screened as they were quite obviously ***not*** cases of monogenic diabetes, therefore a lower estimate of the prevalence of monogenic diabetes might be appropriate (34/2288 = 1·5%),<br>2. there were no differences between those not screened and those who were screened, and so the base case numbers would not change (34/1407 = 2·4%)<br>3. those 881 who did not complete screening were ***more*** likely to be cases of monogenic diabetes. As an upper estimate, we assume the prevalence of monogenic diabetes in the defined cohort is doubled (68/1407 = 4·8%).<br>To investigate an increase or decrease in the prevalence of monogenic diabetes, sensitivity analyses assumed scenarios 1 and 3 above.
Sensitivity and specificity of the Ad Hoc Testing strategy	Based on referral rate data for Northern Ireland (the region with the lowest referral rates)[4]	Sensitivity analyses were based on all regions analysed by Shields et al[4]
Sensitivity of UCPCR test	Based on data from Besser et al[5] which used a prevalent case-control diagnostic study design: 0.94 (see Supplementary Data 3).	Since the sensitivity estimate for the UCPCR test is from a case-control diagnostic study, it is likely that the reported estimate will be greater than in practice.<br><br>Threshold analyses assumed sensitivity estimates for the UCPCR test between 1 and 0·55 (in 0.05 decrements). Results assuming a sensitivity of 1 or 0.55 are presented.
Specificity of UCPCR test	Based on data from Besser et al[5] which used a prevalent case-control diagnostic study design: 0.96 (see Supplementary Data 3).	Since the specificity estimate for the UCPCR test is from a case-control diagnostic study, it is likely that the reported estimate will be greater than in practice.<br><br>Threshold analyses assumed specificity estimates for the UCPCR test between 1 and 0·55 (in 0.05 decrements). Results assuming a specificity of 1 or 0.55 are shown.
Sensitivity of autoantibody test	Based on data from MacDonald et al[6] which used a prevalent case-control diagnostic study design: 0.99 (see Supplementary Data 3)	Since the sensitivity estimate for the autoantibody test is from a case-control diagnostic study, it is likely that the reported estimate will be greater than in practice.<br><br>Threshold analyses assumed sensitivity estimates for the autoantibody test between 1 and 0·55 (in 0.05 decrements).<br>Results assuming a sensitivity of 1 or 0.55 are shown.

For peer review only - http://bmjopen.bmj.com/site/about/guidelines.xhtml

Specificity of autoantibody test	Based on data from MacDonald et al[6] which used a prevalent case-control diagnostic study design: 0.82 (see Supplementary Data 3)	Since the specificity estimate for the autoantibody test is from a case-control diagnostic study, it is likely that the reported estimate will be greater than in practice.<br><br>Threshold analyses assumed specificity estimates for the autoantibody test between 1 and 0·55 (in 0.05 decrements).<br>Results assuming a sensitivity of 1 or 0.55 are shown.
Uptake of UCPCR test	Based on data from the accompanying clinical study which investigated the application of the Biomarker strategy.<br>Uptake of UCPCR was assumed to be 88% (see Table 2C above).	Threshold analyses where UCPCR test uptake was assumed to range from 100% to just 10% (in 10% decrements).<br>It was hypothesised that test uptake in practice is likely to be lower than test uptake in the accompanying clinical study where individuals have consented to participating in a study.<br><br>Results of assumptions that uptake of UCPCR is 100% or 10% are reported.
Uptake of autoantibody test	Based on data from the accompanying clinical study which investigated the application of the Biomarker strategy.<br>Uptake of autoantibody testing was assumed to be 92% (see **Error! Reference source not found.** above).	Threshold analyses where autoantibody test uptake was assumed to range from 100% to just 10% (in 10% decrements).<br><br>It was hypothesised that test uptake in practice is likely to be lower than test uptake in the accompanying clinical study where individuals have consented to participating in a study.<br><br>Results of assumptions that uptake of autoantibody testing is 100% or 10% are reported.
Uptake of genetic test	Based on data from the accompanying clinical study which investigated the application of the Biomarker strategy.<br>Uptake of genetic testing was assumed to be the same as for autoantibody testing (92%) since the same blood sample for autoantibody testing was used for the genetic testing (see **Error! Reference source not found.** above).	Threshold analyses where genetic test uptake was assumed to range from 100% to just 10% (in 10% decrements).<br><br>It was hypothesised that test uptake in practice is likely to be lower than test uptake in the accompanying clinical study where individuals have consented to participating in a study.<br><br>Results of assumptions that uptake of genetic testing is 100% or 10% are reported.
Repeat urine samples and UCPCR tests	Based on data from the accompanying clinical study which investigated the application of the Biomarker strategy. The percentage of repeat urine samples and UCPCR tests was assumed to be 2% (see **Error! Reference source not found.** above).	Threshold analyses were undertaken assuming no repeats, 1%, 5%, 10%, 20%, 50%, 100%, 150% and 200% of samples and tests needed to be repeated. 200% repeat samples and tests can be interpreted as every individual requiring another 2 urine samples and UCPCR tests to be done, so that in total every individual has provided 3 urine samples and 3 UCPCR tests have been done – an extreme assumption.<br><br>Results for assuming 200% repeat samples and tests are presented.

For peer review only - http://bmjopen.bmj.com/site/about/guidelines.xhtml

Repeat blood samples and autoantibody tests	Based on data from the accompanying clinical study which investigated the application of the Biomarker strategy. The percentage of repeat blood samples and autoantibody tests was assumed to be 2% (see **Error! Reference source not found.** above).	Threshold analyses were undertaken assuming no repeats, 1%, 5%, 10%, 20%, 50%, 100%, 150% and 200% of samples and tests needed to be repeated. 200% repeat samples and tests can be interpreted as every individual requiring another 2 blood samples and autoantibody tests to be done, so that in total every individual has provided 3 blood samples and 3 autoantibody tests have been done, clearly an extreme assumption.<br><br>Results for assuming 200% repeat samples and tests are presented.
Percentage of individuals with *GCK* mutation who are receiving insulin treatment at the start of the model	Based on data from the accompanying clinical study which investigated the application of the Biomarker strategy. 88% of individuals with *GCK* mutation are receiving insulin treatment at the start of the model, while 12% are receiving tablets (metformin and sulphonylureas). See **Error! Reference source not found.** above.	Threshold analyses assuming 100% to 10% (in 10% decrements) of individuals with *GCK* mutations are receiving insulin at the start of the model.<br><br>Results from assuming 100% or 10% are receiving insulin at the start of the model are presented.
Percentage of individuals with *HNF1A* or *HNF4A* mutation who are receiving insulin treatment at the start of the model	Based on data from the accompanying clinical study which investigated the application of the Biomarker strategy. 78% of individuals with *HNF1A* or *HNF4A* mutation are receiving insulin treatment at the start of the model, 5% are receiving insulin and tablets (metformin and sulphonylureas), 14% are receiving tablets and 3% are not treated pharmacologically. See **Error! Reference source not found.** above	Threshold analyses assuming 100% to 10% (in 10% decrements) of individuals with *HNF1A* or *HNF4A* mutations are receiving insulin at the start of the model.<br><br>Results from assuming 100% or 10% are receiving insulin at the start of the model are presented.
Genetic test cost	UK referral centre costs[7]: £350 for *GCK* mutation; £450 for *HNF1A* and *HNF4A* mutations, see Supplementary Data 4.	Threshold analyses were conducted to identify at what cost of genetic tests would the All Tested strategy incur no additional costs over the No Testing strategy. Costs of tests for GCK and HNF1A and HNF4A mutations were reduced in 10% steps to just 10% of their base case costs: £35 for GCK and £45 for HNF1A and HNF4A.<br><br>Results of assumptions that genetic costs are 100% or 10% of their current costs are reported.
Long-term insulin need for individuals with *HNF1A* or *HNF4A* mutations	Expert 1	Expert 2, who assumed a larger dose of insulin would generally be required sooner than that stated by Expert 1.

For peer review only - http://bmjopen.bmj.com/site/about/guidelines.xhtml

Percentage of individuals with *HNF1A* or *HNF4A* mutations who remain on most appropriate treatment after a diagnosis of monogenic diabetes	Based on data from the accompanying clinical study which investigated the application of the Biomarker strategy. At 1 and 3 months after changing to more appropriate treatment, 86% are receiving tablets only (sulphonylureas and metformin). At 6 and 12 months 89% and 77% are on tablets only, respectively.	The base case estimates are based on a small number of participants. Threshold analyses have been conducted to investigate the percentage of individuals with *HNF1A* or *HNF4A* mutations who need to remain on tablets for the strategies to be cost-saving compared to No Testing.<br><br>It was assume that for all follow-up time periods after a monogenic diabetes diagnosis, the percentage receiving tablets is: 100%, 50%, 25% or 10%.<br><br>Results assuming 100% and 10% receive tablets are presented.
Cascade family testing	Analysis of referral rate data[7] indicate that for every 10 case of monogenic diabetes identified, 6.3 family members are also genetically tested: with 5.9 being positive for monogenic diabetes and 0.4 being negative for monogenic diabetes.	The impact of family cascade testing in the Ad Hoc, Clinical Prediction Model and Biomarker strategies was investigated by removing all cascade family testing from the strategies.<br><br>Estimates of the magnitude of cascade family testing based on the upper 95% confidence interval limits are used where 6.3 family members are found to be positive for monogenic diabetes, and 0.6 are found to be negative for monogenic diabetes, compared to the scenario where there is no family testing.
Frequency of HBGM before and after changing treatment due to a diagnosis of monogenic diabetes	Based on data from the accompanying clinical study which investigated the application of the Biomarker strategy. Data suggested that individuals with *GCK* mutations stopped HBGM after their diagnosis of monogenic diabetes, while individuals with *HNF1A* or *HNF4A* mutations significantly reduced their frequency of HBGM after a diagnosis of monogenic diabetes.	The 95% confidence limits for the estimated frequency of HBGM at the start of the model and at follow-up after a treatment change for individuals with *HNF1A* or *HNF4A* mutations were used in sensitivity analyses. The change in frequency of HBGM before and after a diagnosis of monogenic diabetes was maximised (which would favour strategies to identify cases of monogenic diabetes) by assuming the upper 95% confidence limit at baseline and the lower 95% confidence limits at follow-up. Conversely, the change in frequency of HBGM was minimised (which would not be as favourable to strategies to identify cases of monogenic diabetes) by assuming the lower 95% confidence limit at baseline and the upper 95% confidence limit at follow-up.

UCPCR, urinary c-peptide to creatinine ratio; HBGM, home blood glucose monitoring

For peer review only - http://bmjopen.bmj.com/site/about/guidelines.xhtml

Fig 2A Incremental costs (vs No Testing) for all strategies for reducing percentage of *GCK* cohort starting on insulin

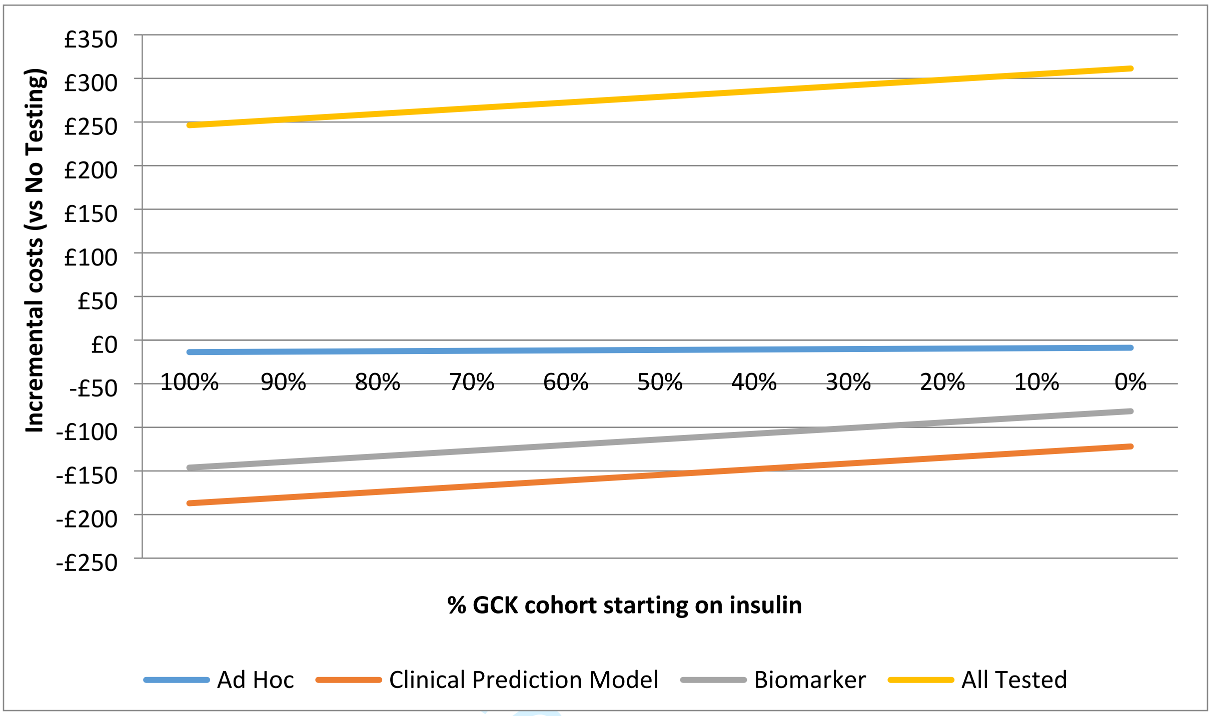

Fig 2B Incremental costs (vs No Testing) for all strategies for reducing percentage of *HNF1A* and *HNF4A* cohort starting on insulin

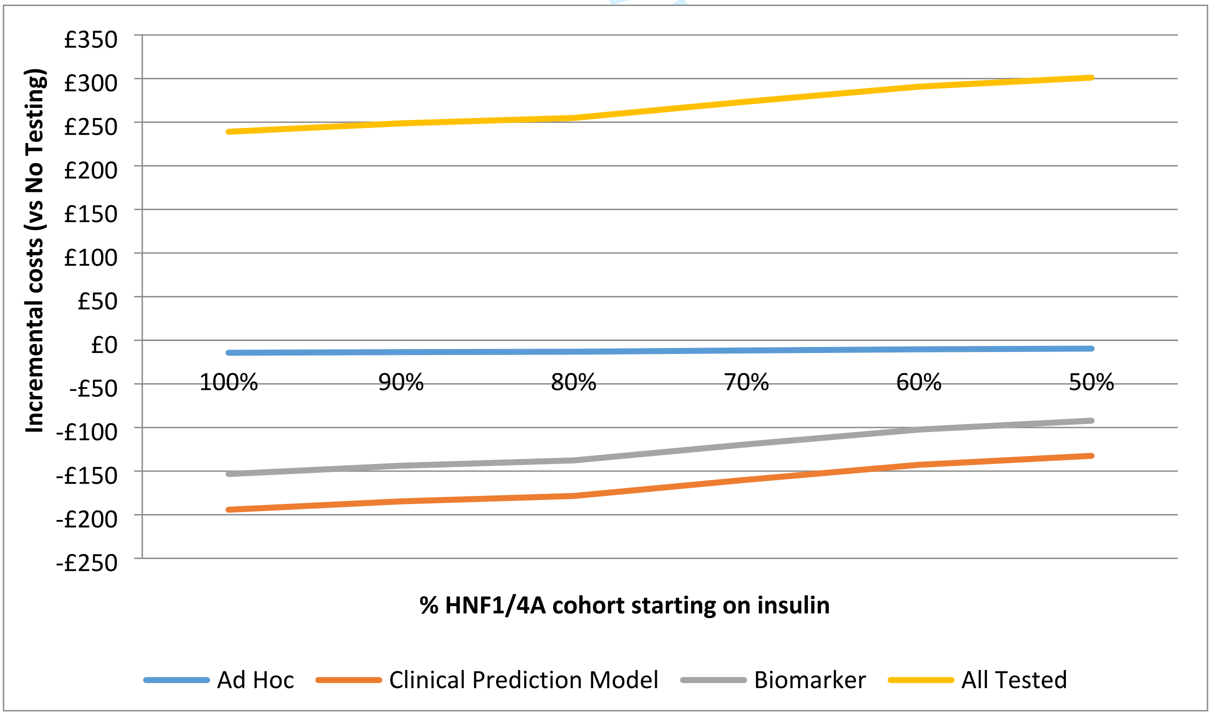

For peer review only - http://bmjopen.bmj.com/site/about/guidelines.xhtml

Fig 2C Incremental costs (vs No Testing) for the Biomarker Testing strategy with reducing levels of UCPCR and antibody testing uptake

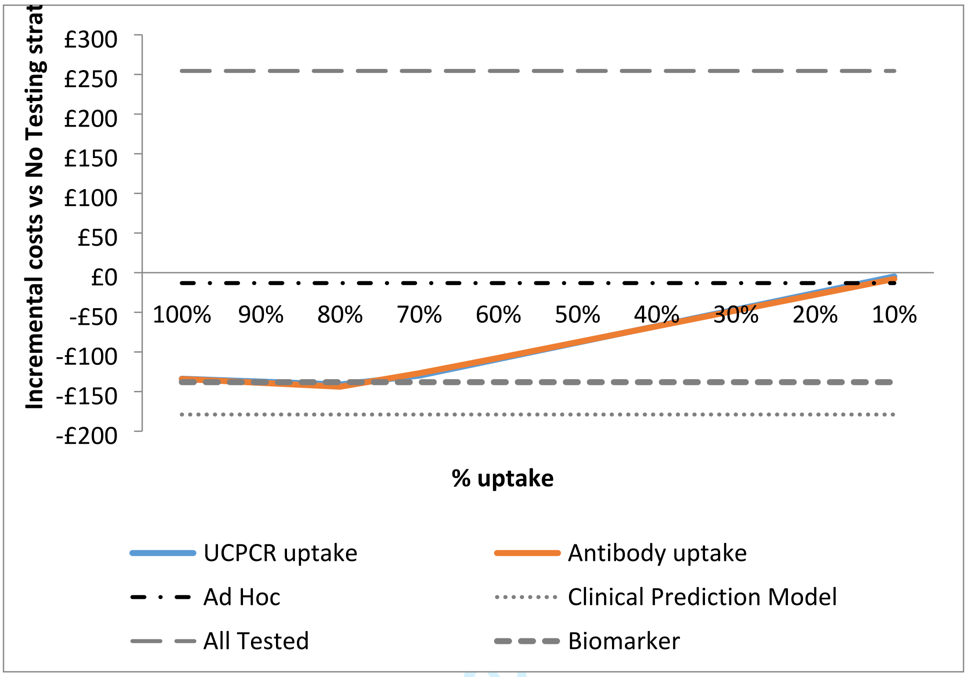

Fig 2D Incremental costs (vs No Testing) for the Biomarker Testing strategy with reducing estimates of sensitivity and specificity for the UCPCR and antibody tests

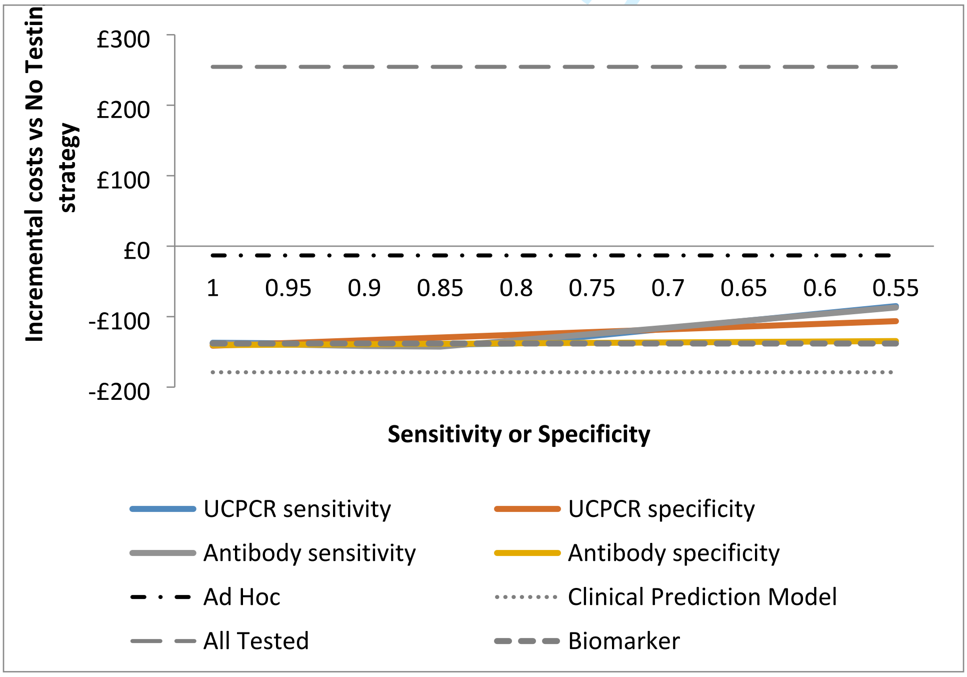

For peer review only - http://bmjopen.bmj.com/site/about/guidelines.xhtml

Fig 2E Incremental costs for the Biomarker Testing strategy (vs No Testing) with increasing estimates of repeat samples and UCPCR and autoantibody tests

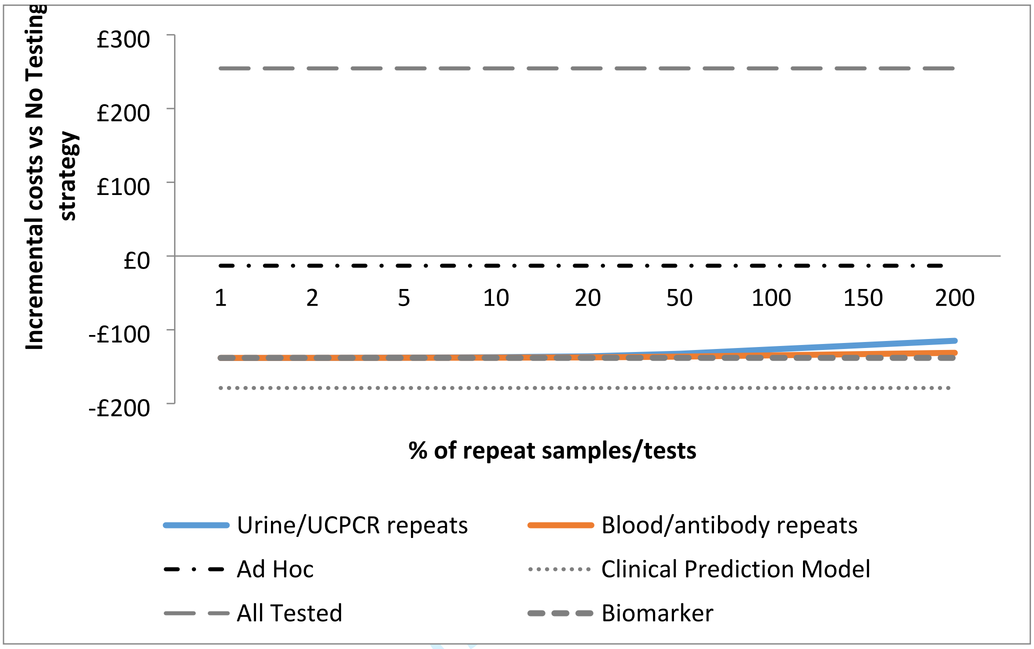

Fig 2F Incremental costs (vs No Testing) for all strategies when genetic test costs are reduced

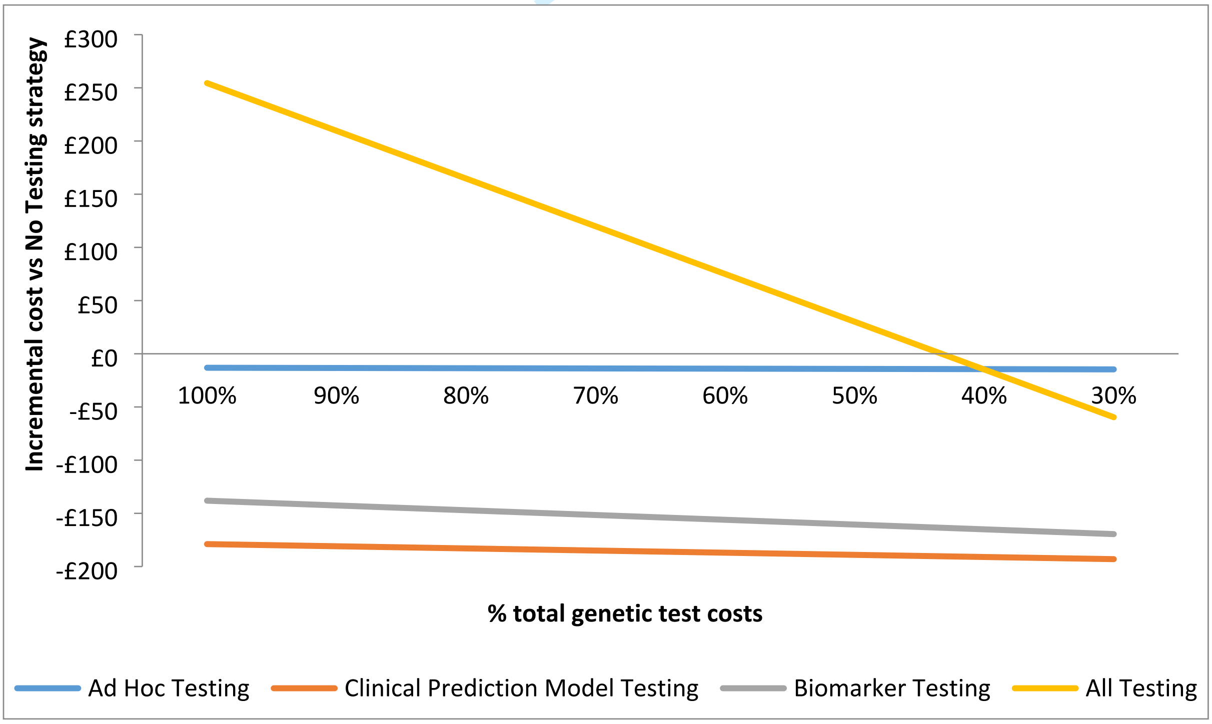

For peer review only - http://bmjopen.bmj.com/site/about/guidelines.xhtml

References

1. Shields BM, Shepherd M, Hudson M, et al. Population-Based Assessment of a Biomarker-Based Screening Pathway to Aid Diagnosis of Monogenic Diabetes in Young-Onset Patients. *Diabetes Care* 2017;40(8):1017-25. doi: 10.2337/dc17-0224 [published Online First: 2017/07/14]
2. Llaurado G, Gonzalez-Clemente J-M, Maymo-Masip E, et al. Serum levels of TWEAK and scavenger receptor CD163 in type 1 diabetes mellitus: relationship with cardiovascular risk factors. A case-control study. *PLoS One* 2012;7(8):e43919.
3. Office for National Statistics. Population Estimates by Ethnic Group 2002-2009. Statistical Bulletin, 2011.
4. Shields BM, Hicks S, Shepherd MH, et al. Maturity-onset diabetes of the young (MODY): how many cases are we missing? *Diabetologia* 2010;53:2504-08.
5. Besser REJ, Shepherd MH, McDonald TJ, et al. Urinary c-peptide-to-creatinine ratio is a practical outpatient tool for identifying hepatocyte nuclear factor 1-α/hepatocyte nuclear factor 4-α maturity-onset diabetes of the young from long-duration type 1 diabetes. *Diabetes Care* 2011;34:1-6.
6. McDonald TJ, Colclough K, Brown R, et al. Islet autoantibodies can discriminate maturity-onset diabetes of the young (MODY) from type 1 diabetes. *Diabetic Medicine* 2011;28:1028-33.
7. Royal Devon and Exeter NHS Foundation Trust. [Available from: http://www.rdehospital.nhs.uk/prof/molecular_genetics/tests/Full_Test_List.htm accessed 13th October 2014.

For peer review only

For peer review only - http://bmjopen.bmj.com/site/about/guidelines.xhtml

Supplementary Data 3: Test-related parameters

Table 3A Summary of the tests involved and estimates of sensitivity and specificity used in the economic evaluation

Test-treatment strategy	Tests used	Sensitivity	Specificity	Data sources
Ad Hoc Testing	Clinical referral based on patient characteristics	0·04	0·996	Shields et al[1]; 2011 census data; Clinical study; Unpublished prevalence data
	Genetic test	1	1	Assumption
Clinical Prediction Model Testing	Type 1 clinical prediction model	0·5 - 0·96	0·65 - 0·996	Shields et al[2]. Estimates of sensitivity and specificity depend on the combination of the probability thresholds used from both clinical prediction models.
	Type 2 clinical prediction model	0·8 - 0·99	0·73 - 0·99	Shields et al[2]. Estimates of sensitivity and specificity depend on the combination of the probability thresholds used from both clinical prediction models.
	Genetic test	1	1	Assumption
Biomarker Testing	UCPCR test	0·94	0·96	Besser et al[3]
	Autoantibody test	0·99	0·82	McDonald et al[4]
	Genetic test	1	1	Assumption
All Testing	Genetic test	1	1	Assumption

UCPCR, urinary c-peptide to creatinine ratio

For peer review only - http://bmjopen.bmj.com/site/about/guidelines.xhtml

Table 3B Sensitivity and specificity of the Ad Hoc Testing strategy by regions in the UK

Region	Sensitivity	Specificity
Northern Ireland[a]	0.038	0.996
Wales	0.044	0.998
Scotland	0.132	0.988
England	0.086	0.993
South West England	0.196	0.977
South East England	0.080	0.995
London	0.049	0.995
East England	0.060	0.996
West Midlands England	0.077	0.994
East Midlands England	0.074	0.995
Yorkshire/Humberside England	0.084	0.996
North East England	0.122	0.994
North West England	0.074	0.995
UK	0.087	0.993
England and Wales	0.084	0.993

[a]Used in base case analysis

References

1. Shields BM, Hicks S, Shepherd MH, et al. Maturity-onset diabetes of the young (MODY): how many cases are we missing? *Diabetologia* 2010;53:2504-08.
2. Shields BM, McDonald TJ, Ellard S, et al. The development and validation of a clinical prediction model to determine the probability of MODY in patients with young-onset diabetes. *Diabetologia* 2012 [published Online First: 5th January 2012]
3. Besser REJ, Shepherd MH, McDonald TJ, et al. Urinary c-peptide-to-creatinine ratio is a practical outpatient tool for identifying hepatocyte nuclear factor 1-α/hepatocyte nuclear factor 4-α maturity-onset diabetes of the young from long-duration type 1 diabetes. *Diabetes Care* 2011;34:1-6.
4. McDonald TJ, Colclough K, Brown R, et al. Islet autoantibodies can discriminate maturity-onset diabetes of the young (MODY) from type 1 diabetes. *Diabetic Medicine* 2011;28:1028-33.

For peer review only - http://bmjopen.bmj.com/site/about/guidelines.xhtml

Supplementary Data 4: Cost parameters

Table 4A Summary of the costs considered for each strategy

	No Testing	Ad Hoc Testing	Clinical Prediction Model Testing	Biomarker Testing	All Testing
Diabetes-specific consultations	o	o	o	o	o
Current treatment	o	o	o	o	o
HBGM on current treatment	o	o	o	o	o
Blood test (for genetic test or autoantibody testing)		o	o	o	o
UCPCR test				o	
Autoantibody test				o	
Genetic test		o	o	o	o
Treatment transfer assistance[a]		o	o	o	o
New treatment		o	o	o	o
HBGM on new treatment		o	o	o	o
Long-term management	o	o	o	o	o

[a]Includes telephone calls with nurse and visit(s) to GP for changes in treatment during 12 month follow-up. UCPCR, urinary c-peptide to creatinine ratio; HBGM, home blood glucose monitoring

Table 4B Costs of testing associated with the strategies

Cost	Value (£, 2018)	Source
GP nurse time for collecting blood sample	£6	10 minutes at £36 per 1hr GP nurse patient contact time[1]
Genetic test for GCK mutation	£350	Sanger sequence analysis from UK referral centre[2]
Genetic test for HNF1/4A mutation	£450	Sanger sequence analysis from UK referral centre[2]
Genetic test for known mutation	£100	Sanger sequence analysis from UK referral centre[2]
Nurse time for successful treatment transfer	£24	Four 10 minute phone calls (expert opinion) at £36 per 1hr GP nurse patient contact time[1]
GP time for informing patient of genetic test result and treatment change	£28	Cost of GP consultation[1]
UCPCR pack	£3·90	Postage
UCPCR test	£10·50	RD&E laboratory[2]
Autoantibody test	£20	RD&E laboratory[2]

UCPCR, urinary c-peptide to creatinine ratio

For peer review only - http://bmjopen.bmj.com/site/about/guidelines.xhtml

Table 4C Cost estimates (£, 2018) used in the IMS CDM model

Event	Cost (£, 2018)	Source
CVD complications		
Myocardial infarction (MI) in 1st year of MI	£7,550	Clarke[3]
Second and subsequent yrs after an MI	£1,250	Clarke[3]
Angina in 1st year of angina	£250	Ward[4]
Second and subsequent yrs after an angina	£200	Ward[4]
Congestive heart failure (CHF) in 1st year of CHF	£3,500	Clarke[5]
Second and subsequent yrs after a CHF	£500	Clarke[5]
Stroke in 1st year of stroke	£4,600	Clarke[3]
Second and subsequent yrs after a stroke	£850	Clarke[3]
Stroke death within 30 days of stroke	£6,350	Clarke[3]
Peripheral vascular disease (PVD) in 1st year of PVD	£1,150	Clarke [5]
Second and subsequent yrs after a PVD	£450	Clarke[5]
Renal complications		
Hemodialysis in 1st year of needing hemodialysis	£43,500	Baboolal[6]
Hemodialysis in second & subsequent yrs of needing hemodialysis	£43,500	Baboolal [6]
Peritoneal dialysis in 1st year of needingperitoneal dialysis	£24,250	Baboolal [6]
Peritoneal dialysis in second & subsequent yrs of needing peritoneal dialysis	£24,250	Baboolal [6]
Renal transplant in 1st year of needing renal transplant	£13,100	NHS Schedule Reference costs[7]; Wight [8]
Renal transplant in second & subsequent yrs of needing renal transplant	£7,050	Wight[8]
Acute events		
Major hypoglyceamic event	£200	Hammer[9]
Minor hypoglyceamic event	£0	Would not require medical assistance
Ketoacidosis event	£1,250	Scuffham[10]
Lactic acid event	£2,500	Curtis[11]
Edema onset	£50	Curtis[11]
Edema follow-up	£0	Assume no follow-up
Eye disease		
Laser treatment	£100	NHS Schedule Reference costs[7]
Cataract operation	£800	NHS Schedule Reference costs[7]
Following cataract operation	£550	Clarke[3]
Blindness in the yr of onset	£7,250	Mitchell[12]
Blindness in the following yrs	£7,250	Mitchell[12]
Neuropathy/foot ulcer		
Neuropathy in the first yr	£150	BNF[13]
Neuropathy in subsequent yrs	£150	BNF[13]
Amputation (one-off cost)	£7,950	Kerr[14]

For peer review only - http://bmjopen.bmj.com/site/about/guidelines.xhtml

Amputation prosthesis (one-off cost)	£3,200	Kerr [14]
Gangrene treatment	£2,700	?
After a healed ulcer	£0	Assumption
Infected ulcer	£4,050	Kerr [14]
Standard uninfected ulcer	£4,050	Kerr [14]
Healed ulcer in those with an amputation history	£0	Assumption
Other		
Statins	£0	NICE guidance and BNF[13]
Aspirin	£0	NICE guidance and BNF[13]
Angiotensin-converting enzyme (ACE)	£0	BNF
Screening for microalbuminuria	£0	NICE[15]
Screening for gross proteinuria	£0	Assume as for MA
Stopping ACEs due to side effects	£0	Assumptions
Eye screening	£50	NICE [15]
Foot screening programme	£100	NICE[16] and Curtis [17]
Non-standard ulcer treatment (e.g. Regranex)	£0	Assumptions
Anti-depression treatment	£0	Assumptions
Screening for depression	£0	Assumptions

Table 4D Annual number of primary care consultations (taken from Currie et al 2010[18])

Type of consultation	Type 1	Type 2	Type 1 control	Type 2 control	Cost per consultation
GP surgery	7·3	8·7	4·5	5·4	£34
GP home visit	0·3	0·6	0·1	0·4	£41
GP telephone	0·5	0·7	0·3	0·4	£20
Community nurse clinic	0·9	1·5	0·3	0·6	£12
Total cost	£278	£349	£165	£213	
Additional cost over controls	£113	£136			

For peer review only - http://bmjopen.bmj.com/site/about/guidelines.xhtml

References

1. Curtis LA, Burns A. Unit costs of health and social care 2017: Personal Social Services Research Unit, University of Kent, 2017.
2. Royal Devon and Exeter NHS Foundation Trust. [Available from: http://www.rdehospital.nhs.uk/prof/molecular_genetics/tests/Full_Test_List.htm accessed 13th October 2014.
3. Clarke P, Gray A, Legood R, et al. The impact of diabetes-related complications on healthcare costs: results from the United Kingdom Prospective Diabetes Study (UKPDS 65). *Diabetic Medicine* 2003;20:442-50.
4. Ward S, Lloyd Jones M, Pandor A, et al. Statins for the prevention of coronary events. Technology assessment report commissioned by the HTA programme on behalf of the National Institute for Clinical Excellence, 2005.
5. Clarke PM, Glasziou P, Patel A, et al. Event rates, hospital utilization, and costs associated with major complications of diabetes: a multicountry comparative analysis. *PLoS Medicine* 2010;7(2)
6. Baboolal K, McEwan P, Sondhi S, et al. The cost of renal dialysis in a UK setting - a multicentre study. *Nephrology Dialysis Transplantation* 2008;23(6):1982-89.
7. Department of Health. National Schedule of Reference Costs 2012-2013, 2013.
8. Wight J, Chilcott J, Holmes M, et al. The clinical and cost-effectiveness of pulsatile machine perfusion versus cold storage of kidneys for transplantation retrieved from heart-beating and non-heart-beating donors. *Health Technology Assessment* 2003;7(25)
9. Hammer M, Lammert M, Mejias SM, et al. Costs of managing severe hypoglycaemia in three European countries. *Journal of Medical Economics* 2009;12(4)
10. Scuffham P, Carr L. The cost-effectiveness of continuous subcutaneous insulin infusion compared with multiple daily injections for the management of diabetes. *Diabetic Medicine* 2003;20(7):586-93.
11. Curtis L. Unit Costs of Health and Social Care 2013, 2013.
12. Mitchell P, Annemans L, Gallagher M, et al. Cost-effectiveness of ranibizumab in treatment of diabetic macular oedema (DME) causing vision impairment: evidence from the RESTORE trial. *British Journal of Opthalmology* 2011;early online
13. British National Formulary 67. BNF 67 (May 2014) 2014 [
14. Kerr M. Foot care for people with diabetes: the economic case for change: NHS Diabetes, 2012.
15. National Institute for Health and Clinical Excellence. Quality Standards Programme. NICE cost impact and commissioning assessment for diabetes in adults, 2011.
16. National Institute for Health and Clinical Excellence. Diabetes footcare commissioning and benchmarking tool, 2012.
17. Curtis L. Unit Costs of Health and Social Care 2011: PSSRU, 2012.
18. Currie CJ, Gale EAM, Poole CD. Estimation of primary care treatment costs and treatment efficacy for people with type 1 and Type 2 diabetes in the United Kingdom from 1997 to 2007. *Diabetic Medicine* 2010;27(8):938-48.

For peer review only - http://bmjopen.bmj.com/site/about/guidelines.xhtml

Section/item	Recommendation	Reported on page
**Title and abstract**		
Title	Identify the study as an economic evaluation or use more specific terms such as "cost-effectiveness analysis", and describe the interventions compared	1
Abstract	Provide a structured summary of objectives, perspective, setting, methods (including study design and inputs), results (including base case and uncertainty analyses), and conclusions.	2
**Introduction**		
Background and objectives	Provide an explicit statement of the broader context for the study.<br>Present the study question and its relevance for health policy or practice decisions.	5-6
**Methods**		
Target population and subgroups	Describe characteristics of the base case population and subgroups analysed, including why they were chosen.	9-10
Setting and location	State relevant aspects of the system(s) in which the decision(s) need(s) to be made.	7
Study perspective	Describe the perspective of the study and relate this to the costs being evaluated.	14
Comparators	Describe the interventions or strategies being compared and state why they were chosen.	7-9
Time horizon	State the time horizon(s) over which costs and consequences are being evaluated and say why appropriate	7
Discount rate	Report the choice of discount rate(s) used for costs and outcomes and say why appropriate.	15
Choice of health outcomes	Describe what outcomes were used as the measure(s) of benefit in the evaluation and their relevance for the type of analysis performed.	15
Measurement of effectiveness	*Single study-based estimates:* Describe fully the design features of the single effectiveness study and why the single study was a sufficient source of clinical effectiveness data.	
	Synthesis-based estimates: Describe fully the methods used for identification of included studies and synthesis of clinical effectiveness data.	10-12, 13
Measurement and valuation of preference based outcomes	If applicable, describe the population and methods used to elicit preferences for outcomes.	NA
Estimating resources and costs	*Single study-based economic evaluation:* Describe approaches used to estimate resource use associated with the alternative interventions. Describe primary or secondary research methods	

For peer review only - http://bmjopen.bmj.com/site/about/guidelines.xhtml

	for valuing each resource item in terms of its unit cost.<br>Describe any adjustments made to approximate to opportunity costs.	
	Model-based economic evaluation: Describe approaches and data sources used to estimate resource use associated with model health states. Describe primary or secondary research methods for valuing each resource item in terms of its unit cost. Describe any adjustments made to approximate to opportunity costs.	14-15
Currency, price date and conversion rate	Report the dates of the estimated resource quantities and unit costs. Describe methods for adjusting estimated unit costs to the year of reported costs if necessary. Describe methods for converting costs into a common currency base and the exchange rate.	14
Choice of model	Describe and give reasons for the specific type of decision analytical model used. Providing a figure to show model structure is strongly recommended	7
Assumptions	Describe all structural or other assumptions underpinning the decision-analytical model.	7-9, 12, 15
Analytical methods	Describe all analytical methods supporting the evaluation. This could include methods for dealing with skewed, missing, or censored data; extrapolation methods; methods for pooling data; approaches to validate or make adjustments (such as half cycle corrections) to a model; and methods for handling population heterogeneity and uncertainty.	10, 16
**Results**		
Study parameters	Report the values, ranges, references, and, if used, probability distributions for all parameters. Report reasons or sources for distributions used to represent uncertainty where appropriate. Providing a table to show the input values is strongly recommended.	16
Incremental costs and outcomes	For each intervention, report mean values for the main categories of estimated costs and outcomes of interest, as well as mean differences between the comparator groups. If applicable, report incremental cost-effectiveness ratios.	16-18
Characterising uncertainty	*Single study-based economic evaluation:* Describe the effects of sampling uncertainty for the estimated incremental cost and incremental effectiveness parameters, together with the impact of methodological assumptions (such as discount rate, study perspective).	NA

For peer review only

For peer review only - http://bmjopen.bmj.com/site/about/guidelines.xhtml

	Model-based economic evaluation: Describe the effects on the results of uncertainty for all input parameters, and uncertainty related to the structure of the model and assumptions.	18-21
Characterising heterogeneity	If applicable, report differences in costs, outcomes, or cost effectiveness that can be explained by variations between subgroups of patients with different baseline characteristics or other observed variability in effects that are not reducible by more information.	21
**Discussion**		
Study findings, limitations, generalisability, and current knowledge	Summarise key study findings and describe how they support the conclusions reached. Discuss limitations and the generalisability of the findings and how the findings fit with current knowledge.	21-25
**Other**		
Source of funding	Describe how the study was funded and the role of the funder in the identification, design, conduct, and reporting of the analysis. Describe other non-monetary sources of support.	3
Conflicts of interest	Describe any potential for conflict of interest of study contributors in accordance with journal policy. In the absence of a journal policy, we recommend authors comply with International Committee of Medical Journal Editors recommendations.	26

For peer review only

For peer review only - http://bmjopen.bmj.com/site/about/guidelines.xhtml

BMJ Open

Strategies to Identify Individuals with Monogenic Diabetes: Results of an Economic Evaluation

Journal:	*BMJ Open*
Manuscript ID	bmjopen-2019-034716.R1
Article Type:	Original research
Date Submitted by the Author:	09-Jan-2020

Complete List of Authors:	Peters, Jaime; University of Exeter Medical School, Exeter Test Group<br>Anderson, Rob; University of Exeter, ESMI (Evidence Synthesis & Modelling for Health Improvement)<br>Shields, Beverley; University of Exeter,<br>King, Sophie; Cardiff University, Centre for Trials Research<br>Hudson, Michelle; University of Exeter Medical School, Institute of Biomedical and Clinical Science<br>Shepherd, Maggie; University of Exeter Medical School, NIHR Clinical Research Facility<br>McDonald, Timothy; Royal Devon and Exeter NHS Foundation Trust, ; University of Exeter, NIHR Exeter Clinical Research Facility<br>Pearson, Ewan; University of Dundee, Division of Molecular & Clinical Medicine<br>Hattersley, Andrew; University of Exeter Medical School, Institute of Biomedical Science<br>Hyde, Chris; University of Exeter Medical School, Exeter Test Group

**Primary Subject Heading**:	Diabetes and endocrinology
Secondary Subject Heading:	Diagnostics, Health economics
Keywords:	pharmacogenetics, monogenic diabetes, economic evaluation, decision analytic modelling, tests

SCHOLARONE™
Manuscripts

I, the Submitting Author has the right to grant and does grant on behalf of all authors of the Work (as defined in the below author licence), an exclusive licence and/or a non-exclusive licence for contributions from authors who are: i) UK Crown employees; ii) where BMJ has agreed a CC-BY licence shall apply, and/or iii) in accordance with the terms applicable for US Federal Government officers or employees acting as part of their official duties; on a worldwide, perpetual, irrevocable, royalty-free basis to BMJ Publishing Group Ltd ("BMJ") its licensees and where the relevant Journal is co-owned by BMJ to the co-owners of the Journal, to publish the Work in this journal and any other BMJ products and to exploit all rights, as set out in our licence.

The Submitting Author accepts and understands that any supply made under these terms is made by BMJ to the Submitting Author unless you are acting as an employee on behalf of your employer or a postgraduate student of an affiliated institution which is paying any applicable article publishing charge ("APC") for Open Access articles. Where the Submitting Author wishes to make the Work available on an Open Access basis (and intends to pay the relevant APC), the terms of reuse of such Open Access shall be governed by a Creative Commons licence – details of these licences and which Creative Commons licence will apply to this Work are set out in our licence referred to above.

Other than as permitted in any relevant BMJ Author's Self Archiving Policies, I confirm this Work has not been accepted for publication elsewhere, is not being considered for publication elsewhere and does not duplicate material already published. I confirm all authors consent to publication of this Work and authorise the granting of this licence.

For peer review only

Strategies to Identify Individuals with Monogenic Diabetes: Results of an Economic Evaluation

Jaime L Peters[1,2]*, Rob Anderson[3], Beverley M Shields[4], Sophie King[4,#a], Michelle Hudson[4], Maggie Shepherd[4], Timothy J McDonald[4], Ewan R Pearson[5], Andrew T Hattersley[4], Chris J Hyde[1]

[1]Exeter Test Group, University of Exeter Medical School, University of Exeter, South Cloisters, St Luke's Campus, Exeter, UK

[2]Collaboration for Leadership in Applied Health Research and Care South West Peninsula (NIHR CLAHRC South West Peninsula), University of Exeter Medical School, University of Exeter, South Cloisters, St Luke's Campus, Exeter, UK

[3]Evidence Synthesis & Modelling for Health Improvement, University of Exeter Medical School, University of Exeter, South Cloisters, St Luke's Campus, Exeter, UK

[4]NIHR Exeter Clinical Research Facility, University of Exeter Medical School, University of Exeter, Barrack Road, Exeter, UK

[5]Division of Cardiovascular & Diabetes Medicine, Medical Research Institute, University of Dundee, UK

[#a] Current Address: Peninsula Clinical Trials Unit, Plymouth University Peninsula Schools of Medicine and Dentistry, Plymouth Science Park, Plymouth, PL6 8BX, UK

* Corresponding author, email: j.peters@exeter.ac.uk

For peer review only

For peer review only - http://bmjopen.bmj.com/site/about/guidelines.xhtml

Abstract

Objectives: To evaluate and compare the lifetime costs associated with strategies to identify individuals with monogenic diabetes and change their treatment to more appropriate therapy.

Design: A decision analytic model from the perspective of the National Health Service (NHS) in England and Wales was developed and analysed. The model was informed by the literature, routinely collected data and a clinical study conducted in parallel with the modelling.

Setting: Secondary care in the UK.

Participants: Simulations based on characteristics of patients diagnosed with diabetes <30 years old.

Interventions: Four test-treatment strategies to identify individuals with monogenic diabetes in a prevalent cohort of diabetics diagnosed under the age of 30 years were modelled: clinician-based genetic test referral, targeted genetic testing based on clinical prediction models, targeted genetic testing based on biomarkers, and blanket genetic testing. The results of the test-treatment strategies were compared to a strategy of no genetic testing.

Primary and secondary outcome measures: Discounted lifetime costs, proportion of cases of monogenic diabetes identified.

Results: Based on current evidence, strategies using clinical characteristics or biomarkers were estimated to save approximately £100-£200 per person with diabetes over a lifetime

For peer review only - http://bmjopen.bmj.com/site/about/guidelines.xhtml

compared to no testing. Sensitivity analyses indicated that the prevalence of monogenic diabetes, the uptake of testing, and the frequency of home blood glucose monitoring had the largest impact on the results (ranging from savings of £400 to £50 per person), but did not change the overall findings. The model is limited by many model inputs being based on very few individuals, and some long-term data informed by clinical opinion.

Conclusions: Costs to the NHS could be saved with targeted genetic testing based on clinical characteristics or biomarkers. More research should focus on the economic case for the use of such strategies closer to the time of diabetes diagnosis.

Strengths and limitations of this study:

- Model structure was informed by expert consultation and critical appraisal of existing models
- Parameter values were taken from a UK-based clinical study conducted alongside this economic evaluation
- Wide-ranging sensitivity analyses were conducted
- Many parameters were based on low numbers of patients
- Evidence on effectiveness was limited.

Funding statement: This study was supported by the Department of Health and Wellcome Trust Health Innovation Challenge Award (HICF-1009-041 and WT-091985). JP is partly supported by the NIHR Collaboration for Leadership in Applied Health Research and Care for the South West Peninsula (PenCLAHRC). BMS, MH and ATH are core members of the National Institute for Health Research (NIHR) Exeter Clinical Research Facility. TJM is

For peer review only

For peer review only - http://bmjopen.bmj.com/site/about/guidelines.xhtml

supported by an NIHR Chief Scientist Office Fellowship. ATH is a Wellcome Trust Senior Investigator and an NIHR Senior Investigator. ERP is a Wellcome Trust New Investigator.

Keywords: costs, decision analytic model, economic evaluation, monogenic diabetes, pharmacogenetics, tests

For peer review only

For peer review only - http://bmjopen.bmj.com/site/about/guidelines.xhtml

Background

Monogenic diabetes is a form of diabetes caused by a mutation in a single gene, which is inherited in an autosomal dominant manner[1]. Therefore a child of an individual with monogenic diabetes has a 50% chance of inheriting the mutation (assuming the child's other parent does not have the mutation). Mutations in glucokinase (*GCK*), hepatocyte nuclear factor 1 alpha (*HNF1A*) and hepatocyte nuclear factor 4 alpha (*HNF4A*) genes are the most common forms of monogenic diabetes.[2] Individuals with mutations in the *GCK gene* have persistently moderately raised blood glucose levels from birth, that is rarely detrimental to health[3] and does not respond to treatment.[4] Therefore individuals with mutations in the *GCK* gene can be successfully treated by diet[4]. Individuals with *HNF1A* or *HNF4A* mutations have blood glucose levels which increase over time and can be successfully treated with sulphonylureas[5] but may, eventually, require insulin treatment.[6]

The minimum prevalence of monogenic diabetes in the UK has been estimated as 108 cases per million.[7] As it usually presents by 25-30 years of age,[1 2 8] individuals are often misdiagnosed with type 1 diabetes, and receive insulin treatment when less invasive and less costly treatment is more appropriate.

The National Health Service (NHS) in England and Wales currently has no national guidelines for identifying individuals with monogenic diabetes. Realistic strategies are available ranging from genetic testing of all individuals with diabetes to targeted genetic testing based on clinical characteristics[9] or biochemical[10] and immunological[11] tests. We report a UK-based economic evaluation of these realistic strategies to identify individuals with monogenic diabetes (defined here as mutations in *GCK*, *HNF1A* or *HNF4A* genes). The development of

For peer review only - http://bmjopen.bmj.com/site/about/guidelines.xhtml

the model-based economic evaluation has been published elsewhere.[12] The economic evaluation was undertaken alongside a clinical study whose aims included (i) investigating the prevalence of monogenic diabetes within two areas of the UK, and (ii) measuring the effects of a change of treatment following a positive diagnosis of monogenic diabetes. The clinical study recruited 1407 individuals who were diagnosed with diabetes <30 years old and who were <50 years old at recruitment[13]. Prospective quality of life (using the EQ-5D Index, a generic measure of health outcome[14]) and glycated haemoglobin (HbA1c) data for 45 individuals who were diagnosed with monogenic diabetes within the geographical areas of the clinical study were collected until 12 months after the genetic test result. Although the clinical study collected data on clinical outcomes, it was not designed, nor powered, to detect small changes in clinical outcomes. No statistically significant change in the EQ-5D Index or HbA1c before and 12 months after changing treatment was observed making it impossible to confirm or refute the clinically suspected benefit of changing treatment in persons found to have monogenic diabetes, but on inappropriate treatment. Thus, only costs are considered in this economic evaluation, making this a conservative analysis of the testing strategies if patient benefit does occur. The implications of this are considered in the discussion.

The aim of this analysis is to evaluate and compare the lifetime costs of different realistic strategies in the NHS to identify individuals with monogenic diabetes and change their treatment to more appropriate therapy. This economic evaluation has been reported in line with CHEERS, the Consolidated Health Economic Evaluation Reporting Standards[15].

For peer review only - http://bmjopen.bmj.com/site/about/guidelines.xhtml

Materials and Methods

Model overview

A hybrid decision model was developed from the perspective of the NHS in England and Wales. A decision tree was developed in MicroSoft Excel to estimate the short-term (16 months) costs, which allowed a maximum of 4 months from referral to testing to change of treatment (for those identified as having monogenic diabetes), plus 12 months follow-up (coinciding with the accompanying clinical study). The IMS CORE Diabetes Model (IMS CDM) version 8.5[16] was used to estimate the lifetime costs associated with the strategies. Expert consultation and explicit critical appraisal of existing long-term diabetes models helped to inform the structure of the decision model and choice of the IMS CDM (see Peters et al[12] for more detail on model development). Evidence to inform the model came from a number of sources including published and unpublished data and clinical opinion. Details on the evidence used in the model are given below.

Strategies and comparator

Five strategies for identifying monogenic diabetes in individuals who were diagnosed with diabetes under the age of 30 years were defined: no genetic testing ("No Testing"), clinician-based genetic test referral ("Ad Hoc Testing"), targeted genetic testing based on clinical prediction models[9] ("Clinical Prediction Model Testing") or biochemical (urinary c-peptide to creatinine ratio, UCPCR[10]) and immunological (islet autoantibodies[11]) test results ("Biomarker Testing"), blanket genetic testing ("All Testing").

The No Testing strategy is the comparator for all other strategies, as it represents the current policy within England and Wales where there is no guidance on the identification of

For peer review only - http://bmjopen.bmj.com/site/about/guidelines.xhtml

individuals with monogenic diabetes. Thus, in this strategy all individuals remain on the diabetes treatment they were receiving at the start of the model, regardless of whether they truly have monogenic diabetes or not.

The Ad Hoc Testing strategy assumes no systematic referral of individuals for monogenic diabetes genetic testing. Instead, individuals are referred on an *ad hoc* basis depending on the awareness of local clinicians of monogenic diabetes (see Fig 1). Data on referral rates for monogenic diabetes genetic testing in the UK[7] were used to calculate estimates of sensitivity and specificity of *ad hoc* referral.

In the Clinical Prediction Model Testing strategy, it is assumed that an individual GP would complete the online monogenic diabetes prediction model (http://www.diabetesgenes.org/content/mody-probability-calculator [9]) to calculate a probability of the individual having monogenic diabetes (see Fig 1). Depending on the probability of the individual having monogenic diabetes as calculated from the prediction model, the GP would then refer them for monogenic diabetes genetic testing or not. Two versions of the prediction model exist, one to distinguish type 1 diabetes from monogenic diabetes (version 1) and the other to distinguish type 2 diabetes from monogenic diabetes (version 2). If the individual is currently receiving insulin, then version 1 of the prediction model is used, otherwise version 2 is used. For each version of the prediction model, nine thresholds are simulated in the decision model. Thus, the Clinical Prediction Model Testing strategy can be evaluated at 81 thresholds (9 from version 1 x 9 from version 2) for the simulated population. The decision model can then be used to identify the probability threshold for the prediction model that maximises the costs saved using the Clinical Prediction Model Testing strategy compared to the No Testing strategy.

For peer review only - http://bmjopen.bmj.com/site/about/guidelines.xhtml

In the Biomarker Testing strategy individuals receive biochemical and/or immunological tests depending on their demonstrated ability to produce insulin (see Fig 2). If individuals are currently receiving insulin treatment, they are offered a UCPCR test to determine whether they are producing insulin or not[10]. Those with a positive UCPCR test are then offered a test for glutamic acid decarboxylase (GAD) and islet antigen2 (IA2) autoantibodies[11]. If individuals are not currently receiving insulin treatment it is assumed they can produce their own insulin and so do not require a UCPCR test. Instead, those individuals not on insulin treatment are offered a test for GAD and IA2 autoantibodies. The aim of the GAD and IA2 autoantibodies test is to rule out those individuals with type 1 diabetes who are still producing insulin (i.e. in the 'honeymoon' period). Individuals not showing the presence of autoantibodies are then offered the monogenic diabetes genetic test. In the All Testing strategy, all individuals are offered monogenic diabetes genetic testing (see Fig 1).

[Fig 1 Simplified model structure for the Ad Hoc Testing, Clinical Prediction Model Testing and All Testing strategies.]

[Fig 2 Simplified model structure for the Biomarker Testing strategy]

Model input parameters

Population characteristics

The main analysis (modelled Cohort 1) simulated a prevalent cohort of individuals in England and Wales who were diagnosed with diabetes when <30 years old and were <50 years old at the start of the model. The prevalence of monogenic diabetes assumed in this

For peer review only - http://bmjopen.bmj.com/site/about/guidelines.xhtml

cohort is 2.4% (*GCK* mutation 0.7%, *HNF1A* mutation 1.5%, *HNF4A* mutation 0.2%). A subgroup analysis (modelled Cohort 2) was undertaken to represent a future incident cohort who would have had a diagnosis of diabetes for a shorter duration than those in Cohort 1. Cohort 2 is defined as individuals diagnosed with diabetes when <30 years old and who were <30 years old at the start of the model, leading to a prevalence of 2.2% having monogenic diabetes. All information relevant to Cohort 2, including parameter values and results, are in Supplementary Data 1. Further data on the prevalence and characteristics of Cohort 1 are given in Supplementary Data 2.

Test characteristics

Details of the test sensitivity and specificity used in the model are shown in Supplementary Data 3. To calculate the sensitivity and specificity of referral for monogenic diabetes genetic testing in the Ad Hoc Testing strategy, four datasets were used:

- diabetes prevalence from unpublished data for Tayside, Scotland
- estimates of total population by age and area from national census[17]
- monogenic diabetes prevalence from the accompanying clinical study[13]
- monogenic diabetes genetic test referral rates[7].

The referral rates for monogenic diabetes genetic testing varied across the UK, with higher referral rates in areas where there is a strong research interest in monogenic diabetes, e.g. the South West of England, and Scotland. Estimates of sensitivity and specificity varied from sensitivity of 0.038 and specificity of 0.996 (Northern Ireland) to sensitivity 0.196 and specificity 0.977 (South West of England), see Supplementary Data 3. To account for the general low rates of referral in the UK, we assumed the referral rates for one of the lowest areas, Northern Ireland. In sensitivity analyses, data from all individual regions were used to

For peer review only - http://bmjopen.bmj.com/site/about/guidelines.xhtml

estimate sensitivity and specificity for the Ad Hoc Testing strategy. However, the cost of increased awareness in one area compared to other areas is not known, and so it is not possible to estimate the additional cost of increased awareness of monogenic diabetes in the Ad Hoc Testing strategy, such as the South West of England and Scotland.

For the Clinical Prediction Model Testing strategy the probability thresholds of 10-90% for the two versions of the test were taken from Shields et al[9], with sensitivity ranging from 0.5-0.99 and specificity ranging from 0.65-0.996. All 81 combinations of probability thresholds were evaluated in the decision model. No adjustments were made to the clinical prediction model as the population on which it would be applied (individuals with diabetes in England and Wales) is very similar to that on which it is based. In the Biomarker Testing strategy, sensitivity of 0.94 and specificity of 0.96 for the UCPCR test was used based on a UCPCR cut-off of ≥0.2nmol/mmol to discriminate individuals with *HNF1A* and *HNF4A* mutations who were insulin treated from individuals with type 1 diabetes[10]. Besser et al did not report on the sensitivity and specificity of this cut-off to discriminate insulin-treated type 2 from *GCK*, *HNF1A* and *HNF4A* mutations, or to discriminate type 1 from *GCK* mutations. Since use of a different UCPCR cut-off for type 1 or insulin-treated type 2 would be difficult in practice (Besser et al[10]), we assumed that the UCPCR cut-off of ≥ 0·2nmol/mmol could be used to discriminate type 1 from insulin-treated type 2, *HNF1A* and *HNF4A* mutations. Furthermore, Besser et al report that UCPCR cannot be used to discriminate *GCK* from *HNF1A* and *HNF4A* mutations. Thus, we assume that the UCPCR cut-off of ≥0.2nmol/mmol can be used to discriminate type 1 diabetes from insulin-treated type 2, *GCK*, *HNF1A* and mutations. The impact on the model results of using different estimates of sensitivity and specificity is assessed in sensitivity analyses. Data from McDonald et al[11] were used to inform the

For peer review only - http://bmjopen.bmj.com/site/about/guidelines.xhtml

sensitivity and specificity for the GAD and IA2 autoantibody tests (see Supplementary Data 3). For all testing strategies, individuals referred for the monogenic diabetes genetic test were either tested for mutations in the *GCK* gene only, the *HNF1A* and *HNF4A* genes together, or all three genes (see Supplementary Data 2).

Uptake and repeat tests

Using data from the accompanying clinical study, for Cohort 1, it was assumed that 8·2% of individuals would decline the offer of genetic testing (6.9% for Cohort 2). This percentage was applied to all of the strategies where genetic testing was an option. For the Biomarker Testing strategy it was assumed that 11·9% for Cohort 1 (12.8% for Cohort 2) of individuals offered the UCPCR test and 8·2% for Cohort 1 (6.9% for Cohort 2) of individuals offered the autoantibody test would not accept. Estimates of the number of repeat tests required for both cohorts in the Biomarker Testing strategy are reported in Supplementary Data 2.

Family genetic testing

It was assumed in the model that identification of an individual with monogenic diabetes from any of the defined strategies would lead to first degree family members (who fit the defined cohort) also being genetically tested. Once individuals identified from the testing strategies have had the genetic test and are found to have monogenic diabetes, their family members receive the monogenic diabetes genetic tests. In Cohort 1, it was assumed that for every 10 individuals identified by the testing strategies as having monogenic diabetes, a further 6·3 family members are genetically tested, with 5.9 of these assumed to have the mutation (based on UK referral rate data[7]). These ratios were applied to the Ad Hoc Testing, Clinical Prediction Model Testing and Biomarker Testing strategies.

For peer review only - http://bmjopen.bmj.com/site/about/guidelines.xhtml

Treatment for diabetes

The treatment pattern assumed at the model start is given in Supplementary Data 2. These data are from the accompanying clinical study where the treatment pattern for those truly having monogenic diabetes is based on just 45 individuals. The impact on the model results of the type of treatment at the start of the model is assessed in sensitivity analyses. Only individuals with a positive genetic test were offered a treatment change; which was cessation of diabetes treatment for those with the *GCK* mutation or to sulphonylureas for individuals with the *HNF1A* or *HNF4A* mutations. Data from the clinical study informed the likely treatment pattern once individuals are diagnosed with monogenic diabetes. For Cohort 1, at 1 month after treatment change it was assumed that 86% of individuals with *HNF1A* or *HNF4A* mutations were receiving a more appropriate treatment, at 3 months this was 86%, at 6 months this was 89% and at 12 months this was 77% (see Supplementary Data 2). Some individuals having a positive genetic test result may not successfully change to sulphonylurea treatment alone and may continue to receive insulin.[18] For individuals with *HNF1A* or *HNF4A* mutations it was assumed that they would require insulin treatment eventually, and how much insulin and when they would start taking it would depend upon whether they had previously received sulphonylureas and progressed to insulin or had started on insulin initially. As no data are available two experts in monogenic diabetes (ATH and EP) were consulted for their opinion (see Supplementary Data 2). Based on data from the accompanying clinical study it was assumed that 93% of individuals identified to have the GCK mutation, would successfully stop all diabetes treatment.

For peer review only - http://bmjopen.bmj.com/site/about/guidelines.xhtml

Resource use

The type of NHS costs (£, inflated to 2018 prices using the Hospital and Community Health Services pay and prices index[19]) considered within each strategy are summarised in Supplementary Data 4.

All treatment costs were estimated using the reported doses from the clinical study and the BNF[20]. The costs associated with the tests include costs for the collection of blood and urine samples, costs of the UCPCR and autoantibody tests and genetic test costs. The costs of nurse time spent providing assistance to those individuals with monogenic diabetes who are changing to a more appropriate treatment were also included. See Supplementary Data 4.

The costs associated with home blood glucose monitoring (HBGM) were also included in the model. The frequency of HBGM before and after diagnosis of monogenic diabetes, and any subsequent change in treatment, was estimated from the clinical study for individuals truly having monogenic diabetes (see Supplementary Data 2). Data from the literature were used to inform HBGM frequency in individuals with type 1 and type 2 diabetes[21 22]. It was assumed that individuals who have a *GCK*, *HNF1A* or *HNF4A* mutation, but did not have a genetic test or change treatment would have the same HBGM frequency as at the start of the model. Costs of HBGM were based on use of the Accu-Check Aviva meter (£16.09 for 50 strips[20]).

The costs of diabetes-related complications for individuals with type 1 diabetes, type 2 diabetes, and *HNF1A* or *HNF4A* mutations were identified from reviewing the published literature and using data from the National Schedule of Reference Costs 2016/17. Only cost data from the UK were modelled in the IMS CDM (see Supplementary Data 4). The majority of cost estimates from the literature were associated with uncertainty, mainly in inflating

For peer review only - http://bmjopen.bmj.com/site/about/guidelines.xhtml

the costs to 2018 due to the age of the evidence available, therefore all of the long-term costs inputted into the model were rounded to the nearest £50 to avoid spurious precision. It is assumed that individuals with *GCK* mutations do not experience long-term diabetes-related complications[3] and once identified as having a mutation in the GCK gene, they no longer incur the costs of diabetes-specific consultations. Data from Curtis 2017[19] and Currie et al 2010[23] were used to inform the costs of diabetes-specific consultations (see Supplementary Data 4).

Long-term events and survival

It was assumed that individuals with *GCK* mutations do not experience diabetes related events and have the same mortality rate as the general population[17]. Therefore inidviduals with GCK mutations do not enter the IMS CDM. For individuals with HNF1A and HNF4A mutations, due to limited data on long-term complications and mortality, it was assumed that these individuals have the same pattern of long-term complications and mortality as individuals with type 1 diabetes. Therefore individuals with HNF1A and HNF4A mutations were modelled using the type 1 diabetes model in the IMS CDM.

Model outcomes

All costs (£, 2018) beyond the first year are discounted at a rate of 3·5% per annum to account for the preference for deferring future costs in economic evaluations.[24] Discounted and undiscounted total costs are reported in the results section alongside the estimated discounted incremental costs per person with diabetes over a lifetime for each strategy compared to the No Testing strategy and the proportion of monogenic diabetes cases identified by each strategy.

For peer review only - http://bmjopen.bmj.com/site/about/guidelines.xhtml

Analysis

The results of a “base case” analysis are presented, but due to the uncertainty surrounding many of the parameter estimates alternative combinations of assumptions may be equally plausible. Therefore, wide-ranging one-way sensitivity and threshold analyses have been conducted to explore the different sources of uncertainty, this includes an analysis where an improvement in utility for those who successfully change treatment is assumed. Details of the sensitivity and threshold analyses undertaken for Cohort 1 can be found in Supplementary Data 2 (see Supplementary Data 1 for details on Cohort 2 analyses). In contrast to our planned analysis[12], we decided not to do a probabilistic analysis because important structural uncertainties in this model could not be fully captured by a probabilistic analysis (it would therefore be misleading).

Patient and Public Involvement

There was no patient and public involvement in the development or analysis of the model.

Results

Cohort 1: diagnosed <30 years old, <50 years old at start of model

For the “base case” analysis, the total discounted costs per person with diabetes over a lifetime were estimated to be £53,500 to £54,000 depending on the strategy used (see Table 1). The All Testing strategy was estimated as the most costly (£54,000), the cheapest options were the Clinical Prediction Model Testing (where the probability thresholds were chosen to maximise costs saved compared to No Testing) and Biomarker Testing strategies

For peer review only - http://bmjopen.bmj.com/site/about/guidelines.xhtml

(£53,600). The No Testing and Ad Hoc Testing strategies were both estimated as £53,700 per person with diabetes over a lifetime. The Ad Hoc Testing strategy was estimated to identify very few cases of monogenic diabetes (6%) compared to the All Testing strategy which was estimated to identify 92% of monogenic diabetes cases. No more than 92% of monogenic diabetes cases can be identified by any strategy due to the assumption that 8% of individuals will not accept an offer of genetic testing for monogenic diabetes. Family testing boosts the detection of monogenic diabetes cases to 92% in the Clinical Prediction Model Testing and Biomarker Testing strategies. The costs saved for these two strategies over the No Testing strategy relate to more individuals getting a monogenic diabetes diagnosis and changing to receive more appropriate treatment which is cheaper and also leads to a reduction in the frequency of HBGM. The All Testing strategy is the most expensive since although more monogenic diabetes diagnoses are made, resulting in fewer treatment and HBGM costs, the costs of genetically testing all individuals diagnosed with diabetes are very high.

Table 1 Summary of the per person lifetime costs[a] and percentage of cases and non-cases genetically tested for each strategy (ordered by increasing cost of strategy)

Strategy	Total undiscounted costs [a]	Total discounted costs [a]	Incremental costs vs No Testing strategy [a]	% who are genetically tested	
				With monogenic diabetes	Without monogenic diabetes
Clinical Prediction	£133,200	£53,600	-£100	92	3

For peer review only - http://bmjopen.bmj.com/site/about/guidelines.xhtml

Model Testing[b]					
Biomarker Testing	£133,300	£53,600	-£100	92	8
Ad Hoc Testing	£133,500	£53,700	0	6	<1
No Testing	£133,600	£53,700	NA	0	0
All Testing	£133,700	£54,000	£300	92	92

[a] rounded to nearest £100.
[b]probability thresholds chosen to maximise costs saved vs No Testing are 12.6% for type 1 vs monogenic diabetes and 75.5% for type 2 vs monogenic diabetes.

As there are 81 different combinations of probability thresholds for the clinical prediction model, the combination of thresholds which maximises the costs saved for the Clinical Prediction Model Testing strategy have been reported above. In Fig 3, all 81 threshold combinations for the clinical prediction model are shown. The Clinical Prediction Model Testing strategy is estimated to identify 74% or 92% of monogenic diabetes cases depending on the probability threshold combinations used to refer individuals for genetic testing. The lifetime costs saved per person with these threshold combinations compared to No Testing vary from £0 to £150.

[Fig 3. Base case incremental costs (vs No Testing) and the proportion of monogenic diabetes cases identified for each strategy.]

Sensitivity analysis results suggest that the impacts on costs in the different scenarios are insensitive to wide-ranging, plausible changes to key model parameters, (see Figs 4a-4d). No

For peer review only - http://bmjopen.bmj.com/site/about/guidelines.xhtml

plausible parameter value changes the finding that the Ad Hoc Testing and Clinical Prediction Model Testing strategies are always estimated to save costs compared to the No Testing strategy. Only extreme assumptions on the uptake of genetic and UCPCR testing (just 10% uptake) suggest fewer costs are saved from the Biomarker Testing strategy when compared to the No Testing strategy. Except for assumptions on test uptake, the estimated cost savings are in the region of £0-£50 per person over a lifetime for the Ad Hoc Testing strategy (see Fig 4), £50-£300 for the Clinical Prediction Model Testing strategy (see Fig 5) and £50-£250 for the Biomarker Testing strategy (see Fig 6). The All Testing strategy is estimated to cost an additional £150-£350 per person over a lifetime compared to the No Testing strategy except when the cost of the genetic test is assumed to be <60% of its current cost (see Fig 7).

[Fig 4 Sensitivity analyses: incremental costs per person over a lifetime for Ad Hoc Testing strategy vs No Testing strategy.]

[Fig 5. Sensitivity analyses: incremental costs per person over a lifetime for Clinical Prediction Model Testing strategy vs No Testing strategy.]

[Fig 6. Sensitivity analyses: incremental costs per person over a lifetime for Biomarker Testing strategy vs No Testing strategy.]

[Fig 7. Sensitivity analyses: incremental costs per person over a lifetime for All Testing strategy vs No Testing strategy.]

As Figs 4-7 show, the findings are most sensitive to:

For peer review only - http://bmjopen.bmj.com/site/about/guidelines.xhtml

- the estimated prevalence of monogenic diabetes within the cohort – increasing prevalence (from 2.4% in Cohort 1 to 4.8%) leads to greater costs saved for the Ad Hoc Testing, Clinical Prediction Model Testing and Biomarker Testing strategies compared to the No Testing strategy,
- the uptake of testing - reduced uptake leads to fewer costs saved for all strategies compared to the No Testing strategy,
- the frequency of HBGM pre and post-treatment change - assuming that individuals change their frequency of HBGM by only a small amount after a diagnosis of monogenic diabetes leads to fewer costs saved compared to the No Testing strategy,
- the proportion of individuals with monogenic diabetes who receive insulin before their monogenic diabetes diagnosis – the larger the proportion receiving insulin before being diagnosed as having monogenic diabetes, the greater the costs saved for all strategies compared to No Testing.

Threshold analysis results (see Supplementary Data 2) suggest that when the genetic tests are reduced to approximately 35% of their current costs, the All Testing strategy incurs no additional costs compared to the No Testing strategy. However, in this situation, the Biomarker Testing and Clinical Prediction Model Testing strategies are estimated to save, approximately £150 per person over a lifetime, compared to the No Testing strategy. Reducing the percentage of individuals with monogenic diabetes who are receiving only insulin at the start of the model has little impact on the incremental costs estimated: even if 10% of individuals with *GCK* mutations or 10% of individuals with *HNF1A* or *HNF4A* mutations are on tablets at the start of the model, slight cost savings are still estimated with

For peer review only

the Clinical Prediction Model Testing and Biomarker Testing strategies compared to the No Testing strategy (see Figs 5 and 6).

Threshold analyses specific to the Biomarker Testing strategy demonstrate that once uptake of the UCPCR and autoantibody tests is reduced to less than 70%, the costs saved with the Biomarker Testing strategy compared to the No Testing strategy reduce. Costs saved with the Biomarker Testing strategy are most sensitive to reductions in the sensitivity of the UCPCR and autoantibody tests. Increases in the number of repeat urine or blood samples and tests required within the Biomarker Testing strategy have little impact on the estimate of costs saved compared to the No Testing strategy.

Cohort 2: diagnosed <30 years, <30 years at start of model

As in Cohort 1, the Clinical Prediction Model Testing and Biomarker Testing strategies are estimated to save £100 per person with diabetes over a lifetime compared to the No Testing strategy, while the All Testing strategy is assumed to cost an additional £300 compared to the No Testing strategy. When compared to Cohort 1, the Clinical Prediction Model Testing and Biomarker Testing strategies are not estimated to save any more costs because of the trade-off between individuals being less likely to be on insulin prior to genetic testing in Cohort 2 (67% vs 83% in Cohort 1) even though they are more likely to successfully change to sulphonylureas than Cohort 1 (100% vs 79% in Cohort 1). Individuals in Cohort 2 were estimated to monitor their blood glucose less frequently before receiving a diagnosis of monogenic diabetes compared to Cohort 1, and so fewer costs are saved from reducing further the HBGM frequency than is the case for Cohort 1. See Supplementary Data 1 for further results, including sensitivity analyses which suggest that estimates of prevalence and testing uptake have the largest impact on the findings (as for Cohort 1).

For peer review only - http://bmjopen.bmj.com/site/about/guidelines.xhtml

Discussion

The Clinical Prediction Model Testing and Biomarker Testing strategies modelled here have been estimated to be cost saving for identifying individuals with monogenic diabetes and changing their treatment compared to the current practice of no genetic testing. Assumptions about the prevalence of monogenic diabetes within the simulated cohort, the uptake of testing and the frequency of HBGM before and after receiving a diagnosis of monogenic diabetes had the largest impact on the findings, but did not change the overall conclusions that targeted strategies are estimated to save costs compared to the No Testing or All Testing strategies. Data on prevalence and test uptake were taken directly from the accompanying clinical study, which is the first to systematically estimate prevalence of monogenic diabetes in the UK[13]. Information on the frequency of HBGM before and after a diagnosis of monogenic diabetes is based on just a small number of individuals, but is currently the best evidence available.

This is the first UK-based economic evaluation of strategies to identify individuals with monogenic diabetes. A published paper documented the development of the model and the intended analysis,[12] and the minor departures from the protocol have been declared and justified. UK data have been used to inform many of the model inputs, for which there was previously no credible evidence. However, due to the rarity of monogenic diabetes, many inputs specific to individuals with monogenic diabetes are based on very few individuals, especially for Cohort 2, or assumptions. For instance, it was assumed that treatment and HBGM frequency data taken from the clinical study at 12 month follow-up remained

For peer review only - http://bmjopen.bmj.com/site/about/guidelines.xhtml

constant over time in the model, with additional long-term treatment data informed by clinical opinion. Until longer follow-up data are available, it is unclear what impact these assumptions may have on the model results.

We simulated 2 cohorts, both based on data from the clinical study. The aim of Cohort 2 was to assess the impact of strategies for identifying monogenic diabetes in individuals more recently diagnosed with diabetes than those in Cohort 1. Although it was anticipated that individuals in Cohort 2 would find it easier to change to more appropriate treatment (because they had not been on their existing treatment for a long time), we actually found that individuals in Cohort 2 were less likely to be on insulin at that point, so costs saved from changing treatment were smaller than for Cohort 1, even though more individuals changed treatment. However this analysis was limited by the low number of participants close to diagnosis for which data were available. Furthermore, the performance of the Clinical Prediction Model Testing and Biomarker Testing strategies are based on prevalent cohorts[9-11] which will impact on their generalisability to an incident cohort (Cohort 2). Thus, there are still many uncertainties associated with the results, including that the IMS CDM has not been validated for monogenic diabetes, so these results should be interpreted with this in mind. Nevertheless, the numerous sensitivity and threshold analyses estimated cost-savings for the Clinical Prediction Model Testing (when choice of thresholds was maximised to save costs) and Biomarker Testing strategies compared to No Testing.

Naylor et al[25] conducted an economic evaluation of genetic testing (akin to our All Testing strategy) for monogenic diabetes in individuals aged 25-40 years who were newly diagnosed with type 2 diabetes compared to no genetic testing from a US health system perspective. Individuals identified as having *HNF1A* or *HNF4A* mutations who successfully transferred to

For peer review only - http://bmjopen.bmj.com/site/about/guidelines.xhtml

sulphonylureas were assumed a HbA1c reduction of 16.4mmol/mol compared to those not changing treatment (based on 6 individuals at 3 months follow-up after treatment change[26]) and a utility increase of 0·13 for transferring from insulin to sulphonylurea treatment (based on evidence from 519 individuals aged 65 years and older with type 2 diabetes[27]). Naylor et al reported a gain of 0·012 quality-adjusted life-years (QALYs) for the testing strategy at an additional cost of $2,400 per person over a lifetime compared to their no testing strategy, resulting in an incremental cost-effectiveness ratio of $205,000 per QALY gained[25]. The additional costs for the genetic testing strategy in Naylor et al[25] are much greater than the All Testing strategy in our evaluation ($2,400 vs £300) because of differences in the populations simulated. In our evaluation a younger diabetes population is assumed, with individuals who truly have monogenic diabetes being more likely to be misdiagnosed with type 1 and receive insulin. The simulated population in Naylor et al is older and explicitly those diagnosed with type 2, therefore are less likely to receive insulin treatment, so have fewer cost savings from changing treatment.

The health impacts assumed by Naylor et al[25] are also different from those observed in our accompanying clinical study. Using the EQ-5D Index, we found little evidence over the 12 month treatment change period for an improvement in utility associated with more appropriate treatment, although the EQ-5D visual analogue scale and the Diabetes Treatment Satisfaction Questionnaire did suggest an improvement at 12 months. Furthermore, in the sample of 28 individuals with *HNF1A* or *HNF4A* mutations who successfully changed to sulphonylureas no statistically significant impact on HbA1c at 12 months after treatment change was found (mean difference of 3·43 mmol/mol (95% confidence interval -2·18, 9·04)). Due to the lack of evidence suggesting an effect on quality

For peer review only - http://bmjopen.bmj.com/site/about/guidelines.xhtml

of life and HbA1c we took the decision to assume there were no differences in quality of life and HbA1c between those identified as having monogenic diabetes and subsequently changing treatment, and those not identified. Our evaluation was conservative, as evidence shows that changing treatment can have a substantial beneficial impact on individuals[28 29]. A sensitivity analysis assuming an improvement in utility for those found to have HNF1A or HNF4A mutations who successfully changed treatment indicated <5 quality-adjusted days were gained from the Clinical Prediction Model, Biomarker and All Testing strategies compared to No Testing. However, generic and relatively simple quality of life measures (e.g. EQ-5D) are likely to be insensitive to the magnitude and type of changes individuals with diabetes might experience when changing to more appropriate treatment. Measuring such changes to quality of life is also limited by the ceiling effect, since these individuals generally constitute a well-controlled, young diabetes population with a good quality of life. Given these limitations we have not considered any reductions in quality of life that may occur during the testing period, especially for those tested but not found to have monogenic diabetes.

A further limitation is in the evidence used to inform the sensitivity and specificity of the testing strategies. For example, the accuracy of antibody testing for the Biomarker strategy is based on a two-gate study design where the test is evaluated by comparing test results in individuals known to have a diagnosis of monogenic diabetes with those newly diagnosed with type 1 diabetes. Such study designs have been shown to lead to overstated accuracy estimates[30].

A limitation of the Ad Hoc testing strategy is in choosing the referral rates that are representative. We used referral rates for the area with the lowest rate of referral. We

For peer review only - http://bmjopen.bmj.com/site/about/guidelines.xhtml

could have used an average referral rate across the country, but would not have been able to capture the relevant costs of the increased awareness in some areas (such as the South West of the UK where the Referral Centre for monogenic diabetes is based) which is linked to increased referral.

The results suggest that within the context of the NHS, the additional costs of genetically testing (a relatively large number of) individuals are likely to be offset by the lifetime savings from the subsequent treatment changes in a very small proportion of individuals. Although the estimated cost-savings are relatively small per person (approximately £100-£200 over a lifetime), assuming there are approximately 200,000 individuals (personal communication) in England and Wales who are <50 years old and have had a diagnosis of diabetes before the age of 30 years, between £20million and £40million could be saved if such strategies are used. To be able to apply these findings to other populations the cost of the testing in particular will need to be updated. If the genetic test costs are significantly higher, then it is unclear whether the Clinical Prediction model Testing and Biomarker Testing strategies could be considered cost-saving, or even cost-neutral. However, further collection of treatment pattern, HBGM frequency, HbA1c and quality of life data for individuals with monogenic diabetes is required to better inform the decision model, especially to model an incident cohort. Additional strategies to better identify those with monogenic diabetes are feasible, and in development, but will also require evaluation for their effectiveness and cost-effectiveness.

For peer review only

For peer review only - http://bmjopen.bmj.com/site/about/guidelines.xhtml

Conclusions

Targeted strategies to identify individuals with monogenic diabetes and change to more appropriate treatment may be cost saving to the NHS. However, collection of longer-term treatment and frequency of HBGM data would be valuable to reduce the main uncertainties in the modelling. Future work to evaluate the use of genetic testing strategies soon after diagnosis of diabetes would be useful to policy-makers.

Checklist for reporting: see supplementary file for CHEERS checklist.

Data sharing statement: The decision analytic model described in this manuscript is not available due to the IMS CDM being under license for the current study.

Competing interests: The authors declare that they have no competing interests.

Authors' contributions: JP designed the decision model, contributed to data collection, undertook analysis and interpretation of the model results and drafted the manuscript. RA and CH helped design and analyse the decision model, and contributed to the interpretation of the results drafting of the manuscript. BS, MH, MS, TM, EP and AH contributed to the study design and data collection, and commented on the manuscript. SK contributed to data collection and commented on the manuscript.

Acknowledgements: We would like to thank IMS Health for use of the IMS CDM. This research was supported by the National Institute for Health Research (NIHR) Collaboration for Leadership in Applied Health Research and Care South West Peninsula (NIHR CLAHRC South West Peninsula). The views expressed are those of the author(s) and not necessarily those of the NHS, the NIHR or the Department of Health and Social Care.

For peer review only - http://bmjopen.bmj.com/site/about/guidelines.xhtml

References

1. Tattersall RB. Mild familial diabetes with dominant inheritance. *Quarterly Journal of Medicine* 1974;43(170):339-57.
2. Murphy R, Ellard S, Hattersley AT. Clinical implications of a molecular genetic classification of monogenic beta-cell diabetes. *Nature Clinical Practice Endocrinology & Metabolism* 2008;4(4):200-13. doi: 10.1038/ncpendmet0778
3. Steele AM, Shields BM, Wensley KJ, et al. Prevalence of vascular complications among patients with glucokinase mutations and prolonged, mild hyperglycemia. *JAMA-Journal of the American Medical Association* 2014;311(3):279-86.
4. Stride A, Shields B, Gill-Carey O, et al. Cross-sectional and longitudinal studies suggest pharmacological treatment used in patients with glucokinase mutations does not alter glycaemia. *Diabetologia* 2014;57(1):54-56.
5. Pearson ER, Starkey BJ, Powell RJ, et al. Genetic cause of hyperglycaemia and response to treatment in diabetes. *Lancet* 2003;362:1275-81.
6. Thanabalasingham G, Owen KR. Diagnosis and management of maturity onset diabetes of the young (MODY). *British Medical Journal* 2011;343(d6044)
7. Shields BM, Hicks S, Shepherd MH, et al. Maturity-onset diabetes of the young (MODY): how many cases are we missing? *Diabetologia* 2010;53:2504-08.
8. Fajans SS, Bell GI, Bowden DW, et al. Maturity-onset diabetes of the young. *Life Sciences* 1994;55(6):413-22.
9. Shields BM, McDonald TJ, Ellard S, et al. The development and validation of a clinical prediction model to determine the probability of MODY in patients with young-onset diabetes. *Diabetologia* 2012 [published Online First: 5th January 2012]
10. Besser REJ, Shepherd MH, McDonald TJ, et al. Urinary c-peptide-to-creatinine ratio is a practical outpatient tool for identifying hepatocyte nuclear factor 1-α/hepatocyte nuclear factor 4-α maturity-onset diabetes of the young from long-duration type 1 diabetes. *Diabetes Care* 2011;34:1-6.
11. McDonald TJ, Colclough K, Brown R, et al. Islet autoantibodies can discriminate maturity-onset diabetes of the young (MODY) from type 1 diabetes. *Diabetic Medicine* 2011;28:1028-33.
12. Peters JL, Anderson R, Hyde C. Development of an economic evaluation of diagnostic strategies: the case of monogenic diabetes. *BMJ Open* 2013;3
13. Shields BM, Shepherd M, Hudson M, et al. Population-Based Assessment of a Biomarker-Based Screening Pathway to Aid Diagnosis of Monogenic Diabetes in Young-Onset Patients. *Diabetes Care* 2017;40(8):1017-25. doi: 10.2337/dc17-0224 [published Online First: 2017/07/14]
14. The EuroQol Group. EuroQol - a new facility for the measurement of health-related quality of life. *Health Policy* 1990;16(3):199-208.
15. Husereau D, Drummond M, Petrou S, et al. Consolidated Health Economic Evaluation Reporting Standards (CHEERS) statement. *Pharmacoeconomics* 2013;31(5):361-7. doi: 10.1007/s40273-013-0032-y [published Online First: 2013/03/27]
16. Palmer AJ, Roze S, Valentine WJ, et al. The CORE Diabetes Model: Projecting long-term clinical outcomes, costs and cost-effectiveness of interventions in diabetes mellitus (types 1 and 2) to support clinical and reimbursement decision-making. *Current Medical Research and Opinion* 2004;20:S5-S26. doi: 10.1185/030079904x1980
17. Office for National Statistics. Population Estimates by Ethnic Group 2002-2009. Statistical Bulletin, 2011.
18. Shepherd M, Shields B, Ellard S, et al. A genetic diagnosis of HNF1A diabetes alters treatment and improves glycaemic control in the majority of insulin-treated patients. *Diabetic Medicine* 2009;26:437-41.

For peer review only - http://bmjopen.bmj.com/site/about/guidelines.xhtml

19. Curtis LA, Burns A. Unit costs of health and social care 2017: Personal Social Services Research Unit, University of Kent, 2017.
20. National Institute for Health and Clinical Excellence. BNF, 2018.
21. Farmer AJ, Wade AN, French DP, et al. Blood glucose self-monitoring in type 2 diabetes: a randomised controlled trial. *Health Technol Assess* 2009;13(15):iii-iv, ix-xi, 1-50. doi: 10.3310/hta13150 [published Online First: 2009/03/04]
22. Yeaw J, Chan Lee W, Aagren M, et al. Cost of self-monitoring of blood glucose in the United States among patients on an insulin regimen for diabetes. *Journal of Managed Care Pharmacy* 2012;18(1):21-32.
23. Currie CJ, Gale EAM, Poole CD. Estimation of primary care treatment costs and treatment efficacy for people with type 1 and Type 2 diabetes in the United Kingdom from 1997 to 2007. *Diabetic Medicine* 2010;27(8):938-48.
24. National Institute for Health and Clinical Excellence. Guide to the methods of technology appraisal, 2008.
25. Naylor RN, John PM, Winn AN, et al. Cost-effectiveness of MODY genetic testing: translating genomic advances into practical health applications. *Diabetes Care* 2014;37:202-09.
26. Thanabalasingham G, Pal A, Selwood MP, et al. Systematic assessment of etiology in adults with a clinical diagnosis of young-onset type 2 diabetes is a successful strategy for identifying maturity-onset diabetes of the young. *Diabetes Care* 2012;35(6):1206-21.
27. Huang ES, Shook M, Jin L, et al. The impact of patient preferences on the cost-effectiveness of intensive glucose control in older patients with new-onset diabetes. *Diabetes Care* 2006;29(2):259-64.
28. Shepherd M, Hattersley AT. 'I don't feel like a diabetic any more': the impact of stopping insulin in patients with maturity onset diabetes of the young following genetic testing. *Clinical Medicine* 2004;4(2):144-47.
29. Shepherd M, Miles S, Jones J, et al. Differential diagnosis: Identifying people with monogenic diabetes. *Journal of Diabetes Nursing* 2010;14(9):342-47.

30. Rutjes AW, Reitsma JB, Vandenbroucke JP, et al. Case-control and two-gate designs in diagnostic accuracy studies. *Clinical Chemistry* 2005;51(8):1335-41.

For peer review only

For peer review only - http://bmjopen.bmj.com/site/about/guidelines.xhtml

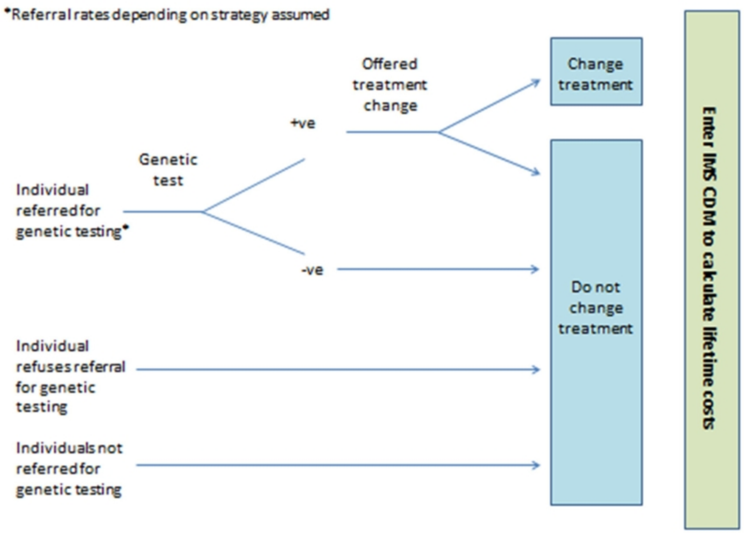

Fig 1 Simplified model structure for the Ad Hoc Testing, Clinical Prediction Model Testing and All Testing strategies

297x209mm (300 x 300 DPI)

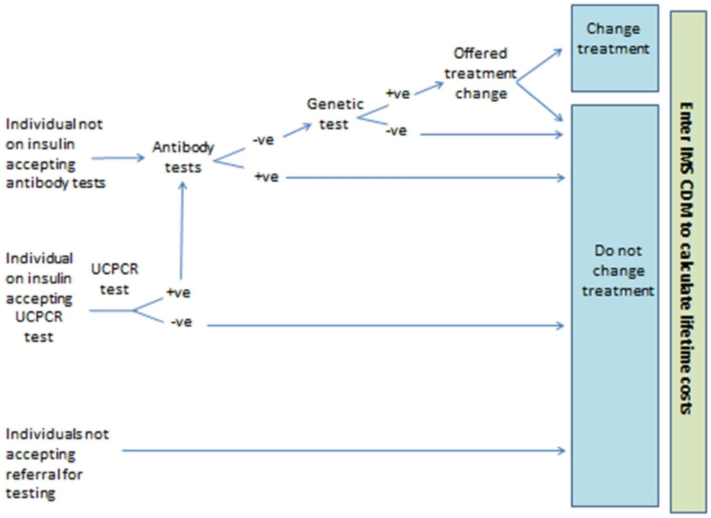

Fig 2 Simplified model structure for the Biomarker Testing strategy

297x209mm (300 x 300 DPI)

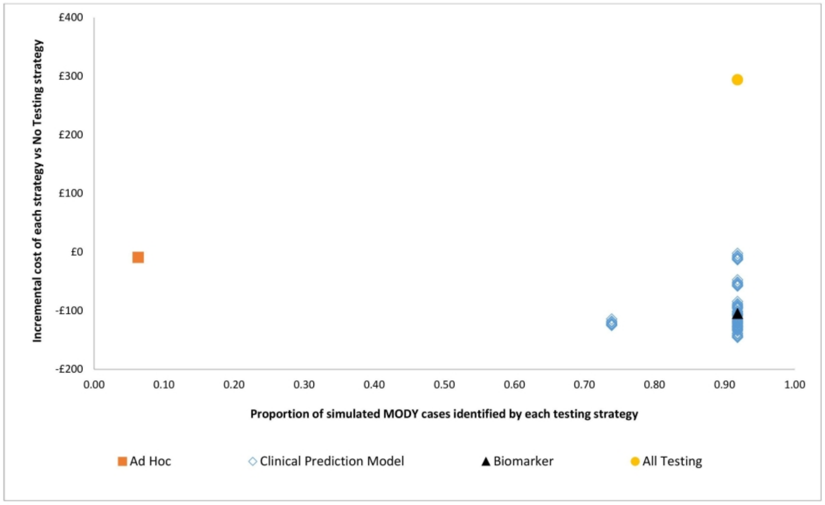

Fig 3. Base case incremental costs (vs No Testing) and the proportion of monogenic diabetes cases identified for each strategy

297x209mm (300 x 300 DPI)

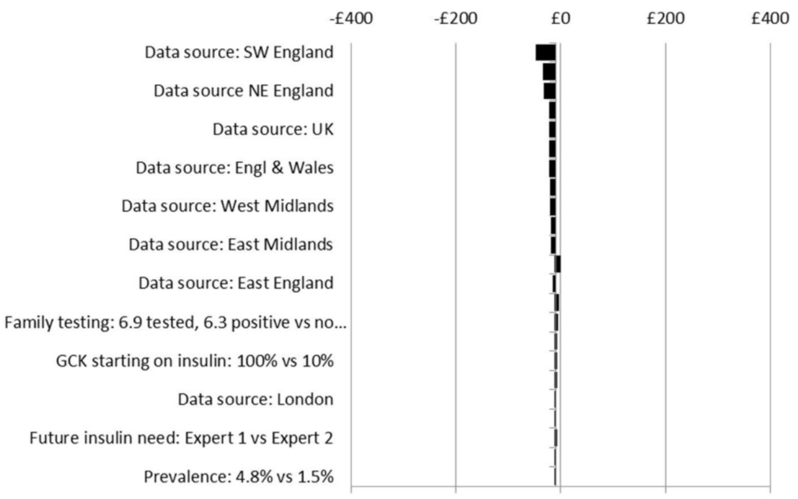

Fig 4. Sensitivity analyses: incremental costs per person over a lifetime for Ad Hoc Testing strategy vs No Testing strategy

148x104mm (300 x 300 DPI)

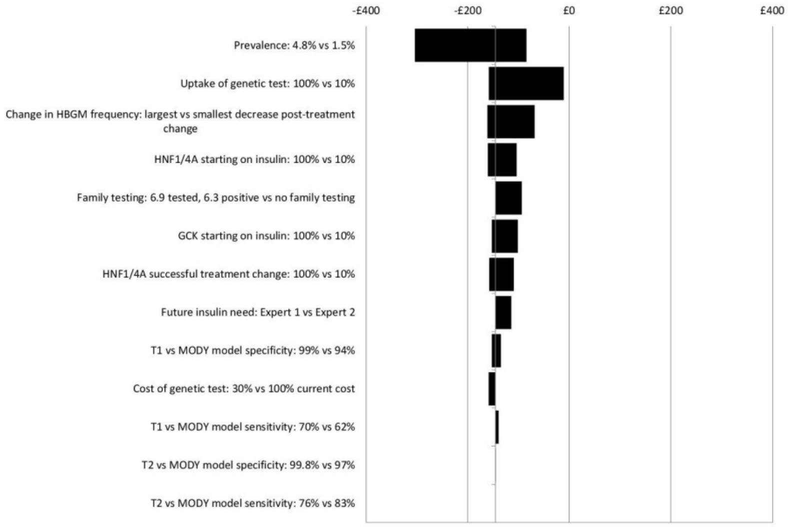

Fig 5. Sensitivity analyses: incremental costs per person over a lifetime for Clinical Prediction Model Testing strategy vs No Testing strategy

148x104mm (300 x 300 DPI)

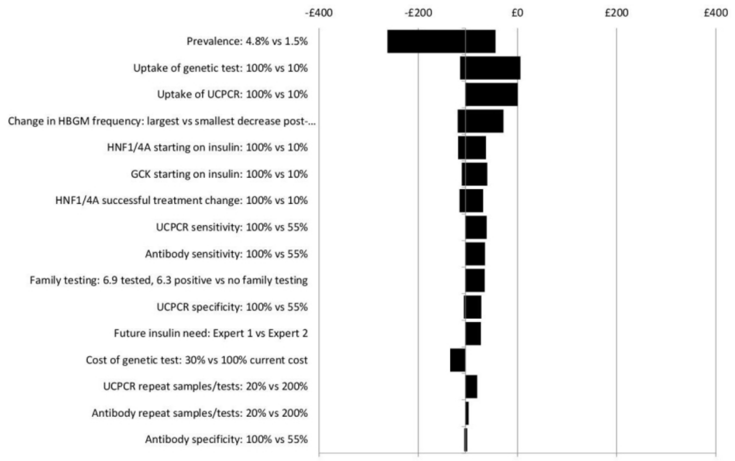

Fig 6. Sensitivity analyses: incremental costs per person over a lifetime for Biomarker Testing strategy vs No Testing strategy

148x104mm (300 x 300 DPI)

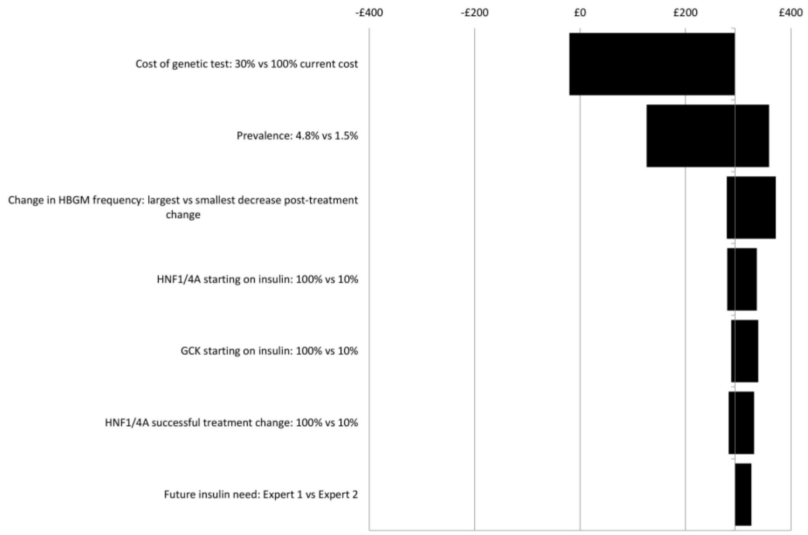

Fig 7. Sensitivity analyses: incremental costs per person over a lifetime for All Testing strategy vs No Testing strategy

297x209mm (300 x 300 DPI)

Supplementary Data 1: Parameters and results for Cohort 2

Cohort 2 - Diagnosed with diabetes <30yrs old and still <30 yrs old at start of model

Table 1A Characteristics of the modelled Cohort 2 at entry to the model

Characteristic	Parameter value	Evidence source
Prevalence (95% confidence interval)		
GCK mutation	1·2% (0.5%, 2.3%)	Shields et al[1] & unpublished data from accompanying clinical study (N=687)
HNF1A mutation	0·9% (0.3%, 1.9%)	Shields et al[1] & unpublished data from accompanying clinical study (N=687)
HNF4A mutation	0.1% (0%, 0.5%)	Shields et al[1] & unpublished data from accompanying clinical study (N=687)
Type 1 diabetes[a]	93·4% (91.3%, 95.2%)	Unpublished data from accompanying clinical study (N=687)
Type 2 diabetes	4·5% (3.1%, 6.3%)	Unpublished data from accompanying clinical study (N=687)
Age (years) [b]	19	Unpublished data from accompanying clinical study (N=687)
Time since diagnosis (years) [b]	8	
Body mass index [b]	25·7	
HbA1c (mmol/mol) [b]	59.8	
Female	50%	
Systolic blood pressure [b]	131·7	[2]
Total cholesterol [b]	4·74	[2]
High density lipoprotein [b]	1·31	[2]
Low density lipoprotein [b]	2·61	[2]
Triglycerides [b]	0·83	[2]
Caucasian	89%	[3]

For peer review only

For peer review only - http://bmjopen.bmj.com/site/about/guidelines.xhtml

Black	4%	3
Asian	7%	3

[a] Defined as receiving insulin treatment within 12 months of diabetes diagnosis.
[b]Mean.

Table 1B Percentage (95% CI) of referred individuals tested for mutations in *GCK* and/or *HNF1A* and *HNF4A* genes by true diagnosis (from unpublished UK referral centre data)

True diabetes diagnosis	Percentage (95% CI) [N=1399]		
	***GCK* only**	***HNF1A* and *HNF4A***	***GCK*, *HNF1A* and *HNF4A***
Not monogenic	15.8% (13.4%, 18.4%)	69.0% (65.8%, 72.0%)	15.2% (12.9%, 17.8%)
GCK mutation	94.6% (91.0%, 97.1%)		5.3% (2.9%, 9.0%)
HNF1A mutation		95.0% (91.0%, 97.6%)	5.0% (2.4%, 9.0%)
HNF4A mutation		96.4% (89.8%, 99.2%)	3.6% (0.8%, 10.2%)

Table 1C Percentage (95% CI) of cohort not accepting offer of testing, or requiring multiple tests for the Biomarker Testing strategy

	Percentage (95% CI)	
Number of tests	UCPCR (including urine sample) N=1299	Autoantibody (including blood sample) N=419
0	12·8% (11.0%, 14.7%)	6·9% (4.7%, 9.8%)
1	84·6% (82.5%, 86.5%)	90·5% (87.2%, 93.1%)
2	2·4% (1.6%, 3.4%)	2·6% (1.3%, 4.6%)
3	0·1% (0.04%, 0.7%)	0%

UCPCR, urinary c-peptide creatinine ratio. Unpublished data from accompanying clinical study.

Table 1D Multipliers (and 95% confidence intervals) to inform cascade genetic testing of diabetic family members

**Number of relatives test per true monogenic diabetes case identified**	**Cohort 2 multiplier**	**Data source**
Relatives positive for monogenic diabetes	5.6 (4.7, 6.5)	Re-analysis of Shields et al[4] (specific to definition of modelled cohort)
Relatives negative for monogenic diabetes	0.6 (0.3, 1.0)	

For peer review only - http://bmjopen.bmj.com/site/about/guidelines.xhtml

Table 1E Pre-genetic treatment pattern, cost and frequency of HBGM by true diagnosis

	Treatment	% receiving treatment	Mean monthly treatment costs	Mean frequency of HBGM[a]
Type 1	Insulin only	100%	£52	78
Type 2	Insulin only	0%	£55	43
	Insulin + tablets	19%	£50	43
	Tablets only	68%	£2	17
	No diabetes treatment	13%	£0	0
GCK	Insulin only	75% (19%, 99%)	£5	52 (0, 110)
	Tablets only	25% (0.6%, 81%)	£1	0
HNF1A or *HNF4A*	Insulin only	67% (35%, 90%)	£18	63 (37, 90)
	Insulin + tablets	0%		
	Tablets	25.0% (6%, 57%)	£1	
	No diabetes treatment	8% (0.2%, 38%)	£0	0

[a]HBGM, home blood glucose monitoring

Table 1F Post-diagnosis HBGM frequency by treatment changed to and true diagnosis

	Time since diagnosis of monogenic diabetes			
	**1 month**	**3 months**	**6 months**	**12 months**
GCK – no diabetes treatment	0	0	0	0
HNF1A and *HNF4A* – tablets only	41 (19, 62)	23 (5, 41)	19 (6, 33)	16 (3, 28)

Table 1G Percentage of individuals with *HNF1A* or *HNF4A* mutations changing to more appropriate treatment after receiving a diagnosis of monogenic diabetes

	Time since treatment change (month)			
	1	3	6	12
Percentage changing to more appropriate treatment	100% (73%, 100%)	100% (73%, 100%)	100% (73%, 100%)	100% (73%, 100%)

For peer review only - http://bmjopen.bmj.com/site/about/guidelines.xhtml

Table 1H Summary of base case, sensitivity and threshold analyses

Parameter	Base case justification	Justification of sensitivity/threshold analyses
Long-term insulin need for individuals with HNF1A or HNF4A mutations	Expert 1	Expert 2, who assumed greater insulin need sooner.
Prevalence of monogenic diabetes	In the accompanying clinical study, the total number of cases of monogenic diabetes was 14 from a total of 687 individuals screened. This leads to an estimated prevalence within the definition of Cohort 1 of 14/687 = 2%.	In sensitivity analyses it was assumed that:<br>1. all of the remaining 993 who were eligible to be screened in the accompanying clinical study would fit the definition for Cohort 2, but were not cases of monogenic diabetes, therefore a lower prevalence of monogenic diabetes was assumed (14/1670 = 0.8%).<br>2. as an upper limit, the prevalence of monogenic diabetes was doubled (28/687 = 4%).
Sensitivity and specificity of the Ad Hoc Testing strategy	Based on referral rate data for Northern Ireland (the region with the lowest referral rates)[4]	Analysed all regions using estimates of sensitivity and specificity given in Supplementary Data 3.
Genetic test cost	UK referral centre costs[5]: £350 for *GCK* mutation; £450 for *HNF1A* and *HNF4A* mutations.	Threshold analyses to identify at what cost of the *GCK* and *HNF1A* and *HNF4A* genetic tests would the All Tested strategy incur no additional costs over the No Testing strategy. Costs of tests for *GCK* and *HNF1A* and *HNF4A* mutations were reduced in 10% steps to just 10% of their base case costs: £35 for *GCK* and £45 for *HNF1A* and *HNF4A*.
Uptake of UCPCR test	Based on data from the accompanying clinical study which investigated the application of the Biomarker Testing strategy.<br>Uptake of UCPCR was assumed to be 87%.	Threshold analyses where UCPCR test uptake was assumed to range from 100% to just 10%.<br>It was hypothesised that test uptake in practice is likely to be lower than test uptake in the accompanying clinical study where individuals have consented to participating in a study.
Uptake of autoantibody test	Based on data from the accompanying clinical study which investigated the application of the Biomarker Testing strategy.<br>Uptake of autoantibody testing was assumed to be 93%.	Threshold analyses where autoantibody test uptake was assumed to range from 100% to just 10%.<br>It was hypothesised that test uptake in practice is likely to be lower than test uptake in the accompanying clinical study where individuals have consented to participating in a study
Uptake of genetic test	Based on data from the accompanying clinical study which investigated the application of the Biomarker Testing strategy.<br>Uptake of genetic testing was assumed to be the same as for autoantibody testing (93%) since the same blood sample for autoantibody testing was used for the genetic testing.	Threshold analyses where genetic test uptake was assumed to range from 100% to just 10%.<br>It was hypothesised that test uptake in practice is likely to be lower than test uptake in the accompanying clinical study where individuals have consented to participating in a study
Repeat urine samples and UCPCR tests	Based on data from the accompanying clinical study which investigated the	Threshold analyses were undertaken assuming no repeats, 1%, 5%, 10%, 20%, 50%, 100%, 150% and 200% of samples and tests needed to be repeated. 200%

For peer review only - http://bmjopen.bmj.com/site/about/guidelines.xhtml

	application of the Biomarker Testing strategy. The percentage of repeat urine samples and UCPCR tests was assumed to be 3%.	repeat samples and tests can be interpreted as every individual requiring another 2 urine samples and UCPCR tests to be done, so that in total every individual has provided 3 urine samples and 3 UCPCR tests have been done – an extreme assumption.
Repeat blood samples and autoantibody tests	Based on data from the accompanying clinical study which investigated the application of the Biomarker Testing strategy. The percentage of repeat blood samples and autoantibody tests was assumed to be 3%.	Threshold analyses were undertaken assuming no repeats, 1%, 5%, 10%, 20%, 50%, 100%, 150% and 200% of samples and tests needed to be repeated. 200% repeat samples and tests can be interpreted as every individual requiring another 2 blood samples and autoantibody tests to be done, so that in total every individual has provided 3 blood samples and 3 autoantibody tests have been done – an extreme assumption.
Sensitivity of UCPCR test	Based on data from Besser et al[6] which used a prevalent case-control diagnostic study design: 0.94.	Since the sensitivity estimate for the UCPCR test is from a case-control diagnostic study, it is likely that the reported estimate will be greater than in practice. Threshold analyses have therefore been undertaken to investigate the impact of assuming lower sensitivity values in particular.<br><br>Threshold analyses assumed sensitivity estimates between 1 and 0·55.
Specificity of UCPCR test	Based on data from Besser et al[6] which used a prevalent case-control diagnostic study design: 0.96.	Since the specificity estimate for the UCPCR test is from a case-control diagnostic study, it is likely that the reported estimate will be greater than in practice. Threshold analyses have therefore been undertaken to investigate the impact of assuming lower specificity values in particular.<br><br>Threshold analyses assumed specificity estimates between 1 and 0·55.
Sensitivity of autoantibody test	Based on data from MacDonald et al[7] which used a prevalent case-control diagnostic study design: 0.99.	Since the sensitivity estimate for the autoantibody test is from a case-control diagnostic study, it is likely that the reported estimate will be greater than in practice. Threshold analyses have therefore been undertaken to investigate the impact of assuming lower sensitivity values in particular.<br><br>Threshold analyses assumed sensitivity estimates between 1 and 0·55.
Specificity of autoantibody test	Based on data from MacDonald et al[7] which used a prevalent case-control diagnostic study design: 0.82.	Since the specificity estimate for the autoantibody test is from a case-control diagnostic study, it is likely that the reported estimate will be greater than in practice. Threshold analyses have therefore been undertaken to investigate the impact of assuming different specificity values.<br><br>Threshold analyses assumed specificity estimates between 1 and 0·55.
Percentage of individuals with *GCK* mutation who are receiving insulin treatment at the	Based on data from the accompanying clinical study which investigated the application of the Biomarker Testing strategy. 75% of individuals with *GCK* mutation are	Threshold analyses assuming 100% to 10% (in 10% decrements) of individuals with *GCK* mutations are receiving insulin at the start of the model.

For peer review only - http://bmjopen.bmj.com/site/about/guidelines.xhtml

start of the model	receiving insulin treatment at the start of the model, while 25% are receiving tablets (metformin and sulphonylureas).	
Percentage of individuals with *HNF1A* or *HNF4A* mutation who are receiving insulin treatment at the start of the model	Based on data from the accompanying clinical study which investigated the application of the Biomarker Testing strategy. 67% of individuals with *HNF1A* or *HNF4A* mutation are receiving insulin treatment at the start of the model, 25% are receiving tablets (metformin and sulphonylureas) and 8% are not treated pharmacologically.	Threshold analyses assuming 100% to 10% (in 10% decrements) of individuals with *HNF1A* or *HNF4A* mutations are receiving insulin at the start of the model.
Percentage of individuals with *HNF1A* or *HNF4A* mutations who remain on most appropriate treatment after a diagnosis of monogenic diabetes	Based on data from the accompanying clinical study which investigated the application of the Biomarker Testing strategy. At every follow-up point after treatment change, 100% of individuals with *HNF1A* or *HNF4A* mutations remained on the most appropriate treatment.	The base case estimates are based on a small number of participants. Threshold analyses have been conducted to investigate the percentage of individuals with *HNF1A* or *HNF4A* mutations who need to remain on tablets for the strategies to be cost-saving compared to No Testing.<br><br>It was assume that for all follow-up time periods after a monogenic diabetes diagnosis, the percentage receiving tablets is: 86%, 77%, 50%, 25% or 10%.
Cascade family testing	Analysis of referral rate data[4] indicate that for every 10 case of monogenic diabetes identified, 6.2 family members are also genetically tested: with 5.6 being positive for monogenic diabetes and 0.6 being negative for monogenic diabetes.	The impact of family cascade testing in the Ad Hoc Testing, Clinical Prediction Model Testing and Biomarker Testing strategies was investigated by removing all cascade family testing from the strategies.<br><br>Estimates of the magnitude of cascade family testing based on the 95% confidence interval limits are used to investigate the impact of this parameter: 4.7 to 6.5 family members who are found to be positive for monogenic diabetes, and 0. 3 to 1 family members who are found to be negative for monogenic diabetes.
Frequency of HBGM before and after changing treatment due to a diagnosis of monogenic diabetes	Based on data from the accompanying clinical study which investigated the application of the Biomarker Testing strategy. Data suggested that individuals with *GCK* mutations stopped HBGM after their diagnosis of monogenic diabetes, while individuals with *HNF1A* or *HNF4A* mutations significantly reduced their frequency of HBGM after a diagnosis of monogenic diabetes.	The 95% confidence limits for the estimated frequency of HBGM at the start of the model and at follow-up after a treatment change for individuals with *HNF1A* or *HNF4A* mutations were used in sensitivity analyses. The change in frequency of HBGM before and after a diagnosis of monogenic diabetes was maximised (which would favour strategies to identify cases of monogenic diabetes) by assuming the upper 95% confidence limit at baseline and the lower 95% confidence limits at follow-up. Conversely, the change in frequency of HBGM was minimised (which would not be as favourable to strategies to identify cases of monogenic diabetes) by assuming the lower 95% confidence limit at baseline and the upper 95% confidence limit at follow-up.

Table 1I Summary of “base case” results

For peer review only - http://bmjopen.bmj.com/site/about/guidelines.xhtml

Strategy	Total undiscounted LYs	Total discounted QALYs	Total discounted costs[a]	Incremental costs vs No Testing strategy[a]	% who are genetically tested	
					With monogenic diabetes	Without monogenic diabetes
Clinical Prediction Model[b]	38·4	11·9	£54,000	-£100	93	3
Biomarker			£54,000	-£100	93	5
Ad Hoc			£54,100	0	7	<1
No Testing			£54,100	NA	0	0
All Testing			£54,400	£300	93	93

[a] rounded to nearest £100; [b] thresholds chosen to maximise costs saved

Fig 1A Incremental costs (vs No Testing) and the proportion of monogenic diabetes cases identified for each strategy

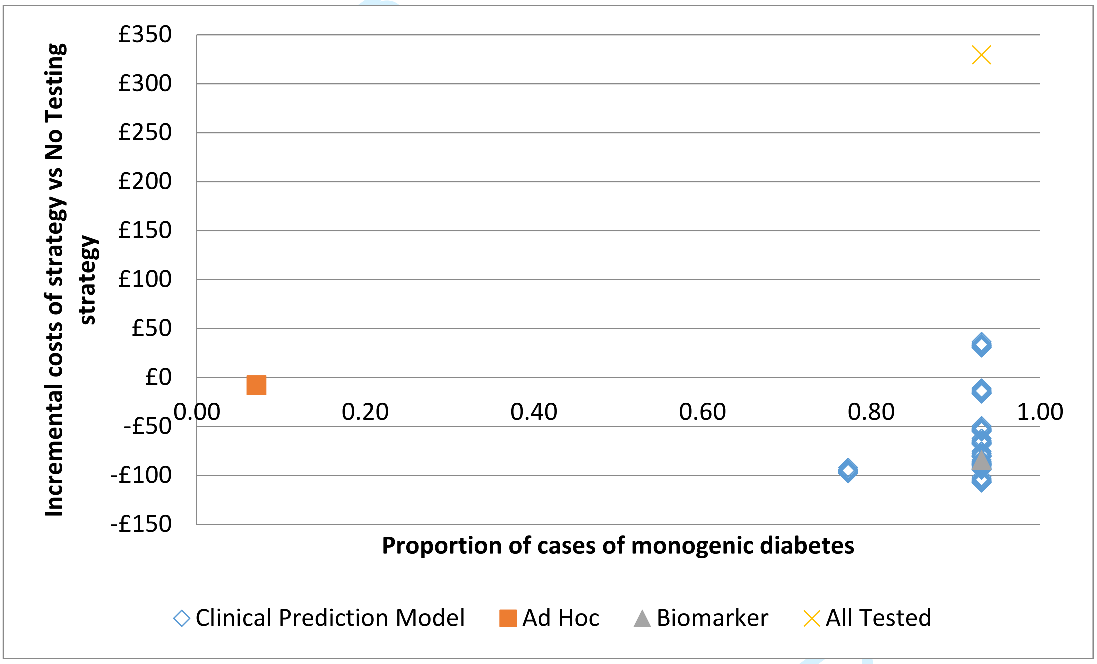

For peer review only - http://bmjopen.bmj.com/site/about/guidelines.xhtml

Fig 1B Tornado plot of sensitivity analyses for the Ad Hoc Testing strategy

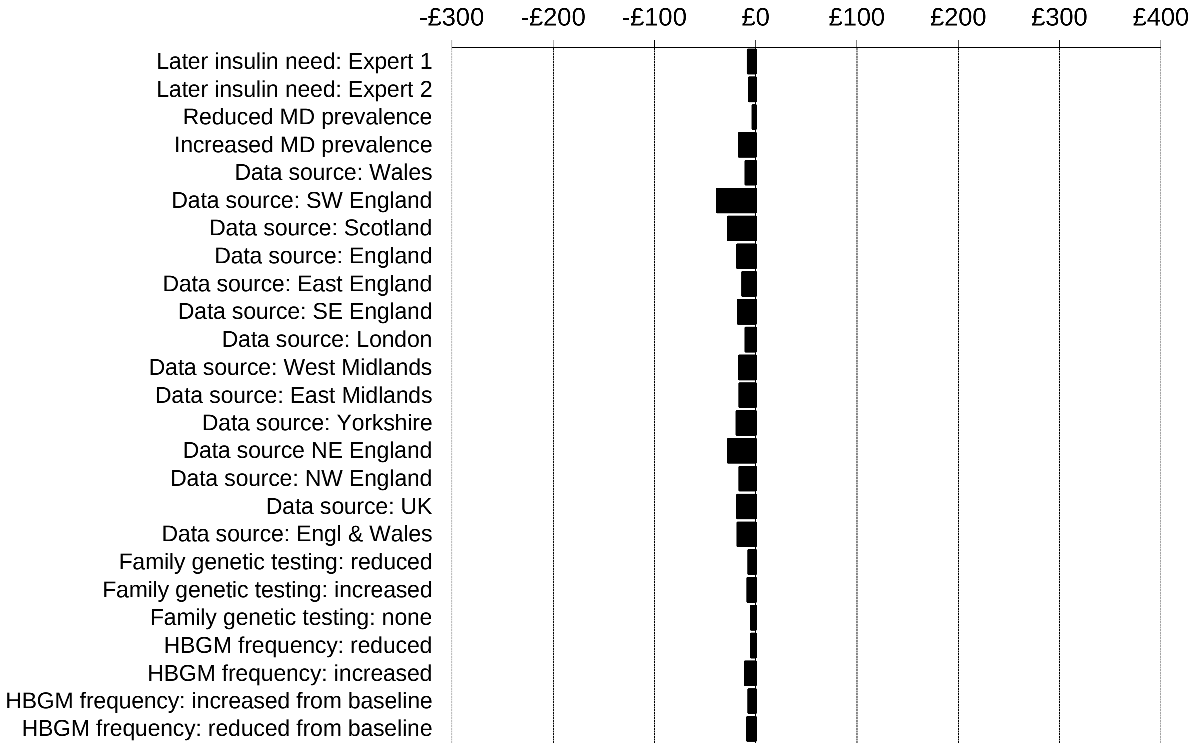

Fig 1C Tornado plot for the Clinical Prediction Model Testing strategy

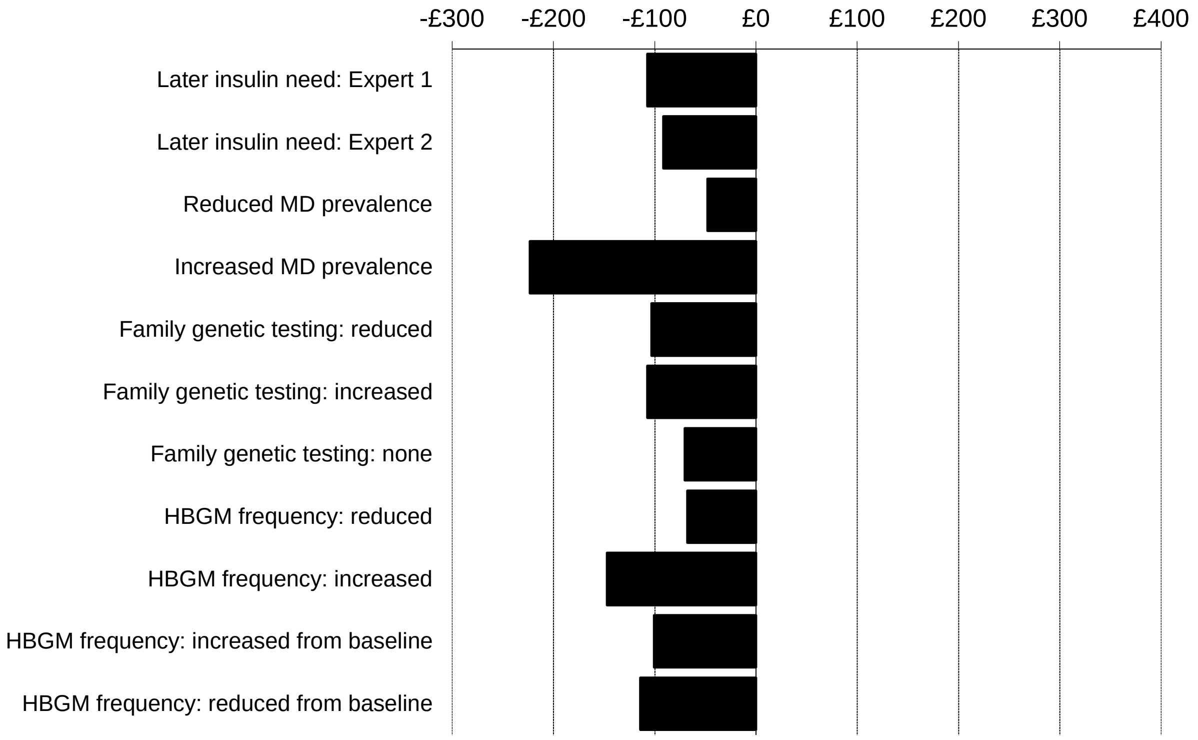

For peer review only - http://bmjopen.bmj.com/site/about/guidelines.xhtml

Fig 1D Tornado plot for the Biomarker Testing strategy

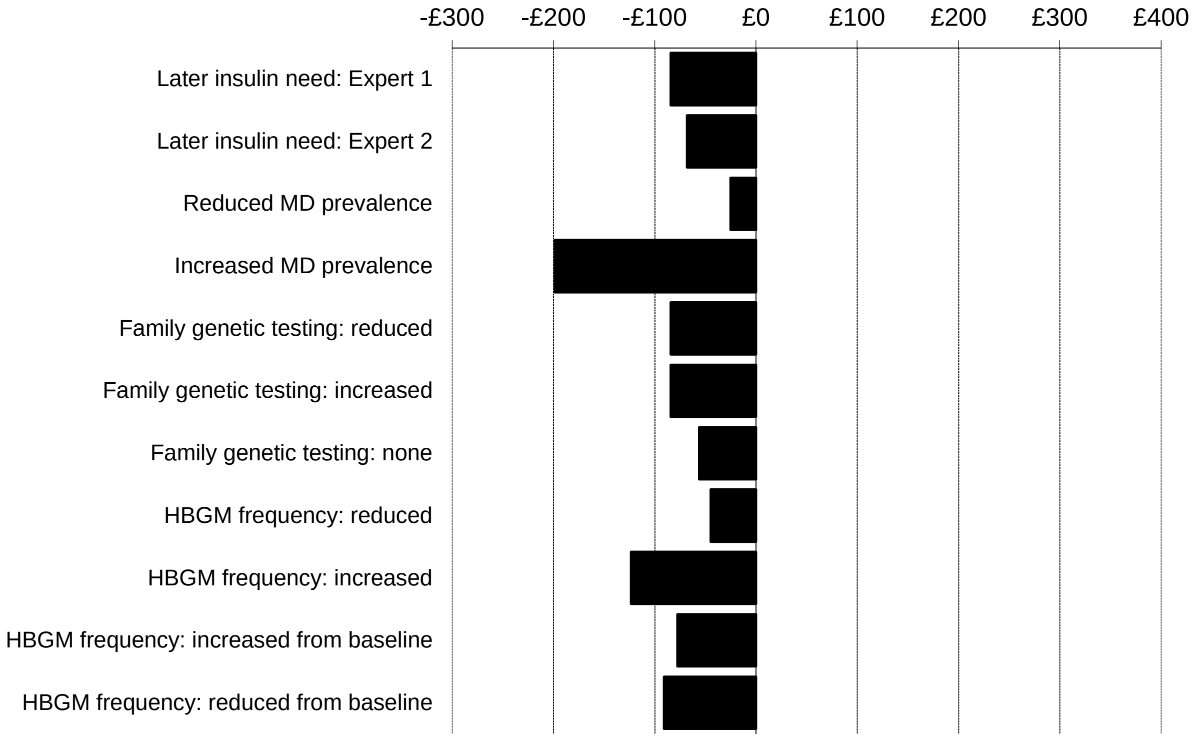

Fig 1E Tornado plot for the All Testing strategy

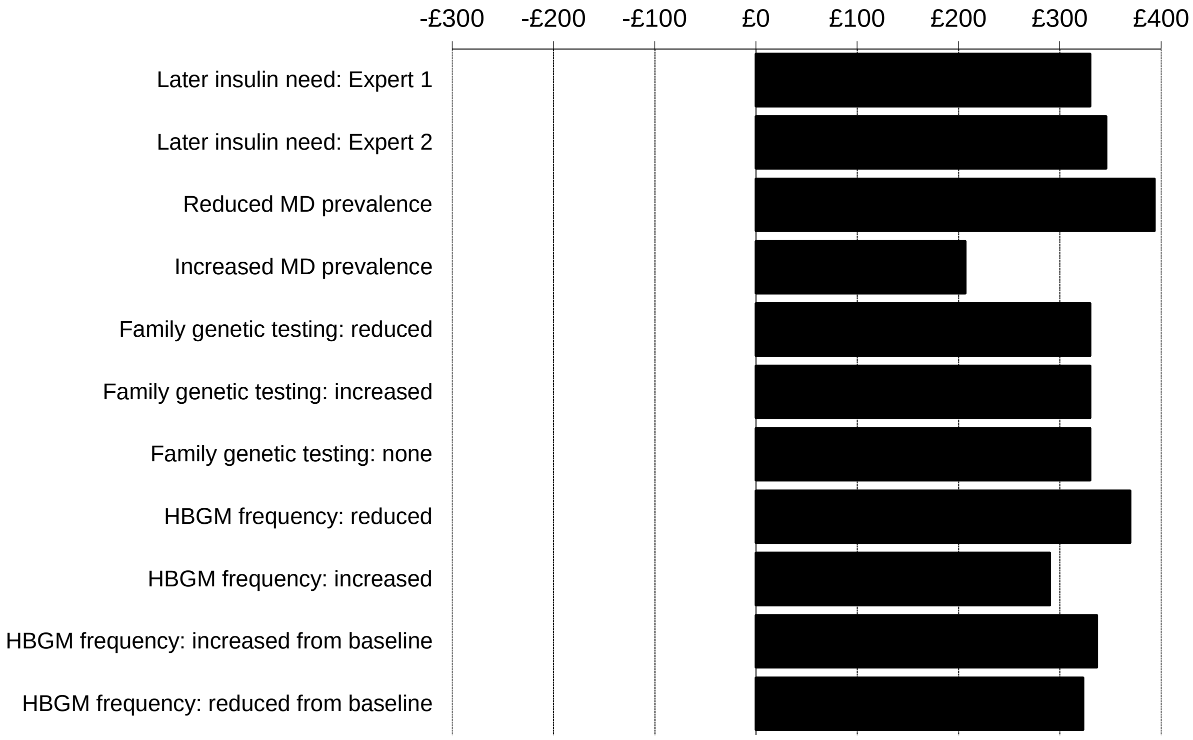

For peer review only - http://bmjopen.bmj.com/site/about/guidelines.xhtml

Fig 1F Incremental costs (vs No Testing) for all strategies for reducing percentage of *GCK* cohort starting on insulin

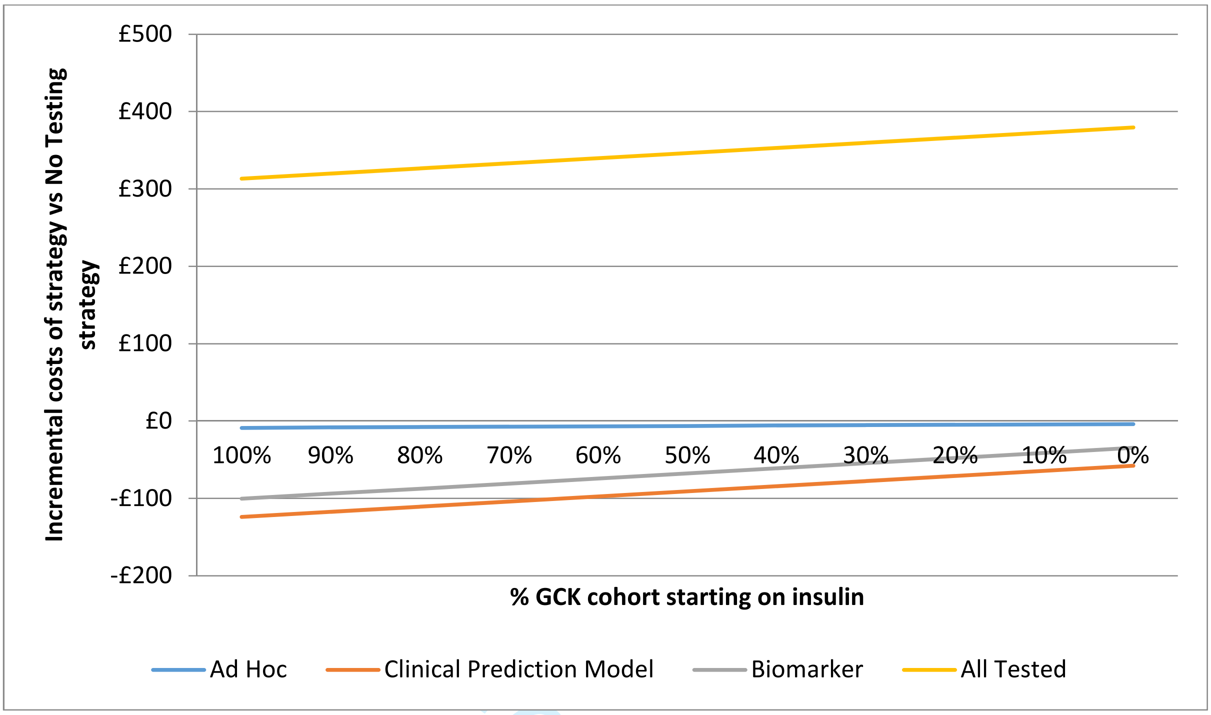

Fig 1G Incremental costs (vs No Testing) for all strategies for reducing percentage of *HNF1A* and *HNF4A* cohort starting on insulin

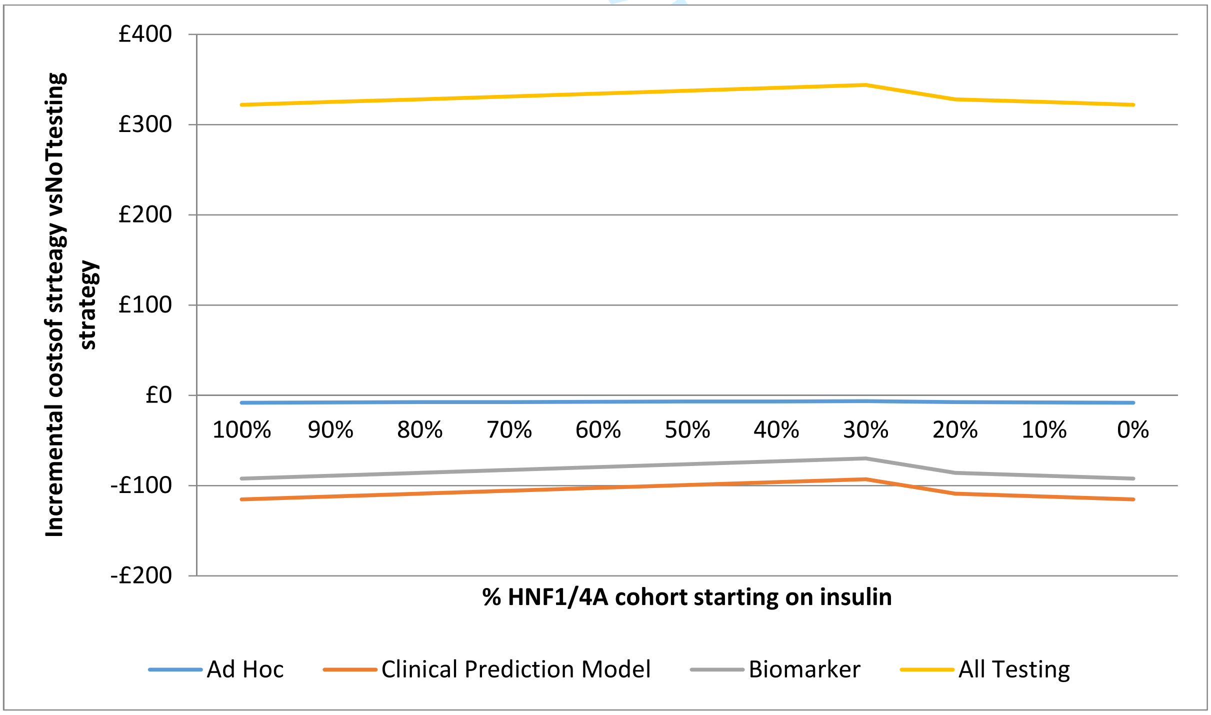

For peer review only - http://bmjopen.bmj.com/site/about/guidelines.xhtml

Fig 1H Incremental costs (vs No Testing) for the Biomarker Testing strategy with reducing levels of UCPCR and antibody testing uptake

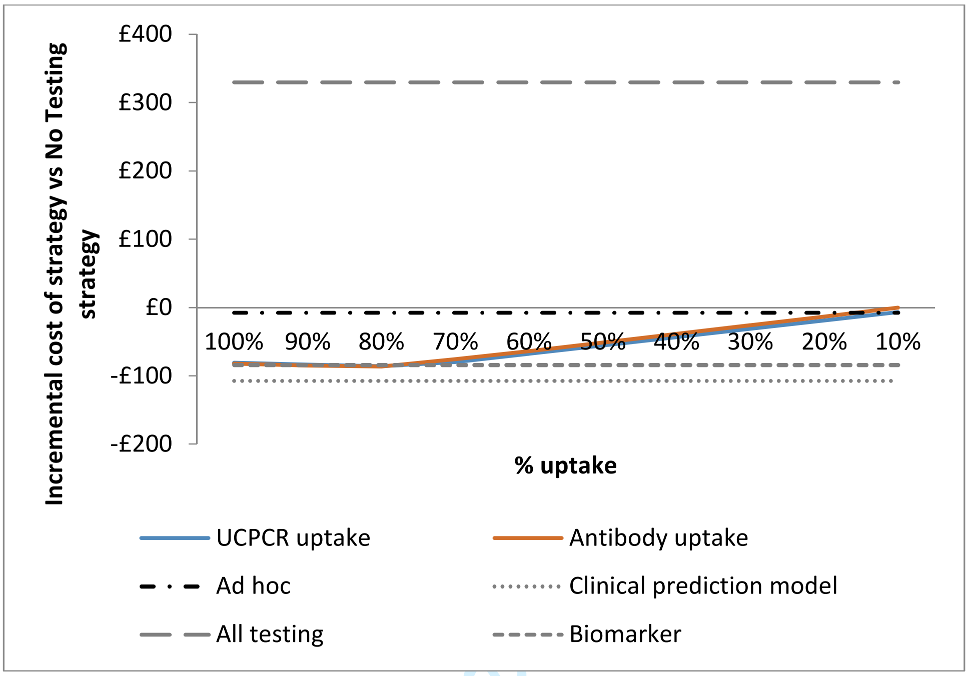

Fig 1I Incremental costs (vs No Testing) for the Biomarker Testing strategy with reducing estimates of sensitivity and specificity for the UCOCR and antibody tests

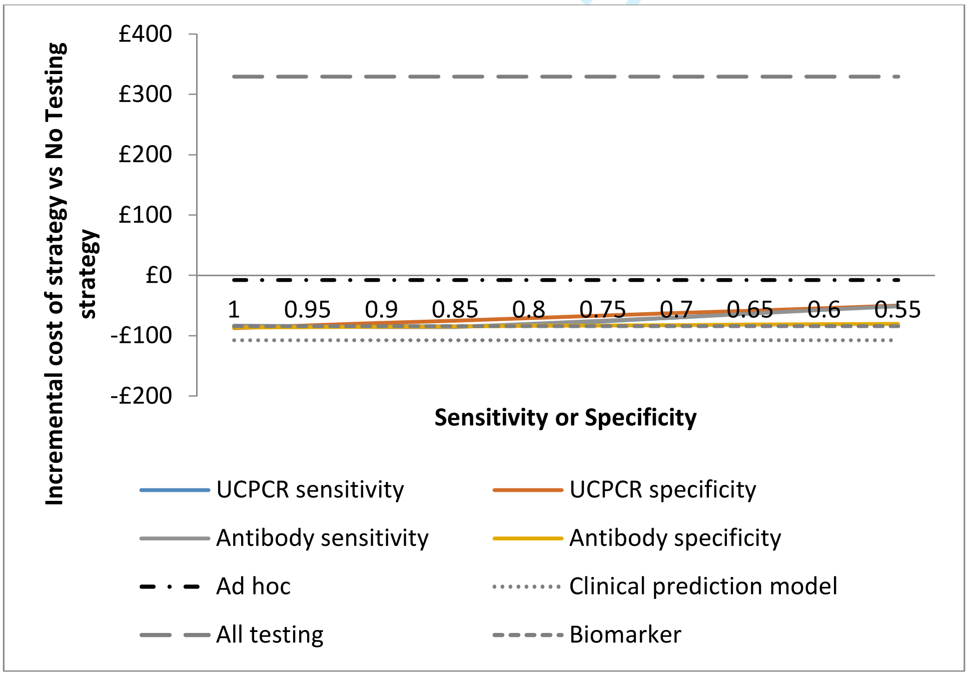

For peer review only - http://bmjopen.bmj.com/site/about/guidelines.xhtml

Fig 1J Incremental costs for the Biomarker Testing strategy (vs No Testing) with increasing estimates of repeat samples and UCPCR and autoantibody tests

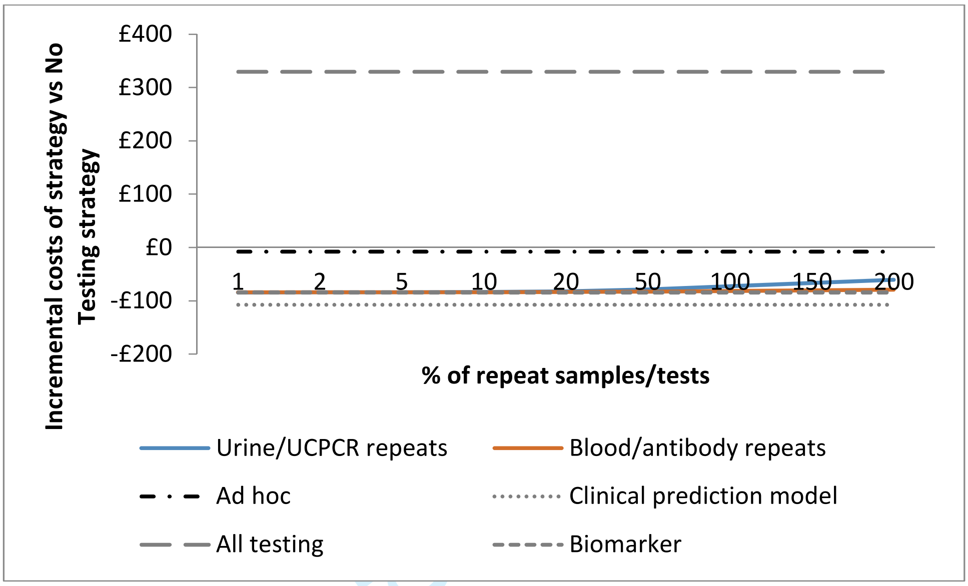

Fig 1K Incremental costs (vs No Testing) for all strategies when genetic test costs are reduced

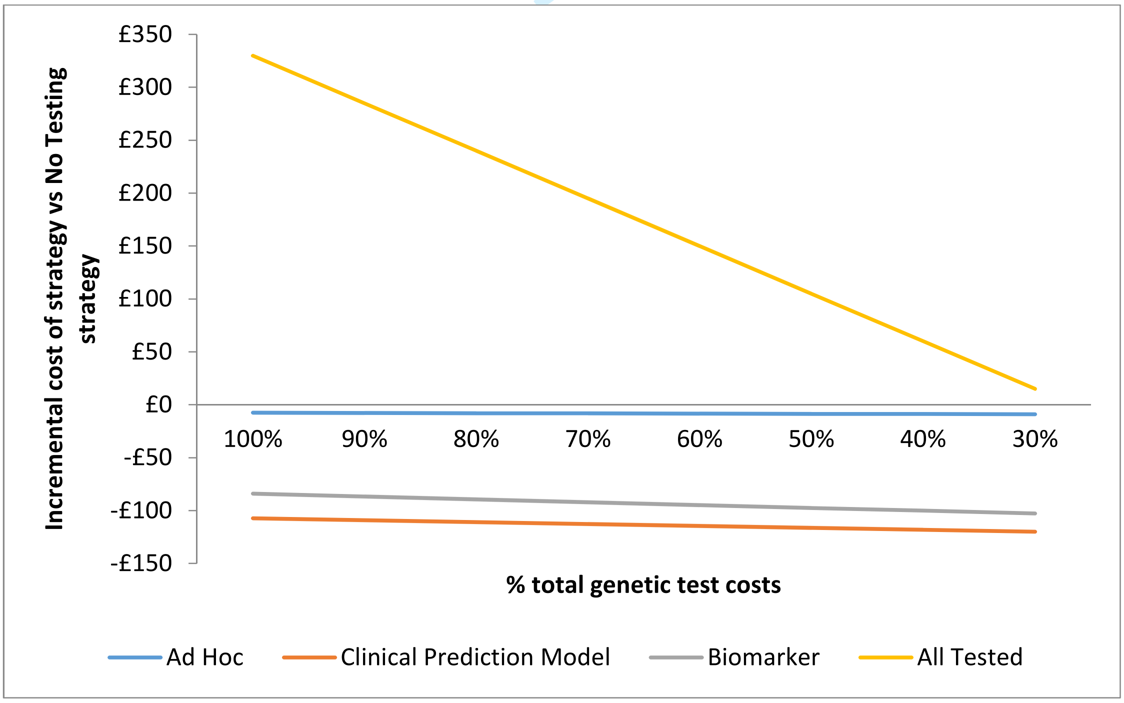

For peer review only - http://bmjopen.bmj.com/site/about/guidelines.xhtml

References

1. Shields BM, Shepherd M, Hudson M, et al. Population-Based Assessment of a Biomarker-Based Screening Pathway to Aid Diagnosis of Monogenic Diabetes in Young-Onset Patients. *Diabetes Care* 2017;40(8):1017-25. doi: 10.2337/dc17-0224 [published Online First: 2017/07/14]
2. Llaurado G, Gonzalez-Clemente J-M, Maymo-Masip E, et al. Serum levels of TWEAK and scavenger receptor CD163 in type 1 diabetes mellitus: relationship with cardiovascular risk factors. A case-control study. *PLoS One* 2012;7(8):e43919.
3. Office for National Statistics. Population Estimates by Ethnic Group 2002-2009. Statistical Bulletin, 2011.
4. Shields BM, Hicks S, Shepherd MH, et al. Maturity-onset diabetes of the young (MODY): how many cases are we missing? *Diabetologia* 2010;53:2504-08.
5. Royal Devon and Exeter NHS Foundation Trust. [Available from: http://www.rdehospital.nhs.uk/prof/molecular_genetics/tests/Full_Test_List.htm accessed 13th October 2014.
6. Besser REJ, Shepherd MH, McDonald TJ, et al. Urinary c-peptide-to-creatinine ratio is a practical outpatient tool for identifying hepatocyte nuclear factor 1-α/hepatocyte nuclear factor 4-α maturity-onset diabetes of the young from long-duration type 1 diabetes. *Diabetes Care* 2011;34:1-6.
7. McDonald TJ, Colclough K, Brown R, et al. Islet autoantibodies can discriminate maturity-onset diabetes of the young (MODY) from type 1 diabetes. *Diabetic Medicine* 2011;28:1028-33.

For peer review only

For peer review only - http://bmjopen.bmj.com/site/about/guidelines.xhtml

Supplementary Data 2: Parameters and results for Cohort 1

Cohort 1 - Diagnosed with diabetes <30yrs old and still <50 yrs old at start of model

Table 2A Characteristics of the modelled cohorts 1 and 2 at entry to the model

Characteristic	Parameter value	Evidence source
Prevalence (95% confidence interval)		
GCK mutation	0·7%<br>(0.4%, 1.4%)	Shields et al[1] & unpublished data from accompanying clinical study (N=1407)
HNF1A mutation	1·5%<br>(1.2%, 2.7%)	Shields et al[1] & unpublished data from accompanying clinical study (N=1407)
HNF4A mutation	0·2%<br>(0.1%, 0.6%)	Shields et al[1] & unpublished data from accompanying clinical study (N=1407)
Type 1 diabetes[a]	88·6%<br>(86.4%, 89.9%)	Unpublished data from accompanying clinical study (N=1407)
Type 2 diabetes	9.0%<br>(7.4%, 10.5%)	Unpublished data from accompanying clinical study (N=1407)
Age (years)[b]	25	Unpublished data from accompanying clinical study (N=1407)
Time since diagnosis (years)[b]	12	
Body mass index[b]	24·4	
HbA1c (mmol/mol)[b]	64.2	
Female (%)	50	
Systolic blood pressure[b]	131·7	[2]
Total cholesterol[b]	4·74	[2]
High density lipoprotein[b]	1·31	[2]
Low density lipoprotein[b]	2·61	[2]
Triglycerides[b]	0·83	[2]
Caucasian	89%	[3]

Black	4%	3
Asian	7%	3

[a] Defined as receiving insulin treatment within 12 months of diabetes diagnosis.
[b]Mean.

Table 2B Percentage (95% CI) of referred individuals tested for mutations in *GCK* and/or *HNF1A* and *HNF4A* genes by true diagnosis (from unpublished UK referral centre data)

True diabetes diagnosis	Percentage (95% CI) [N=2294]		
	***GCK* only**	***HNF1A* and *HNF4A***	***GCK*, *HNF1A* and *HNF4A***
Not monogenic	14.1% (12.3%, 16.0%)	70.0% (67.5%, 72.4%)	15.9% (14.0%, 18.0%)
GCK mutation	95.2% (92.3%, 97.3%)		4.8% (2.7%, 7.7%)
HNF1A mutation		96.2% (94.0%, 97.8%)	3.5% (2.0%, 5.7%)
HNF4A mutation		97.3% (93.2%, 99.2%)	2.7% (0.7%, 6.8%)

Table 2C Percentage (95% CI) of cohort not accepting offer of testing, or requiring multiple tests for the Biomarker Testing strategy

	Cohort 1	
Number of tests	UCPCR (including urine sample) N=2017	Autoantibody (including blood sample) N=624
0	11·9% (10.6%, 13.4%)	8·2% (6.1%, 10.6%)
1	86·1% (84.5%, 87.6%)	90·0% (87.4%, 92.3%)
2	1·8% (1.3%, 2.5%)	1·8% (0.9%, 3.1%)
3	0·1% (0.03%, 0.4%)	0%

UCPCR, urinary c-peptide creatinine ratio. Unpublished data from accompanying clinical study.

Table 2D Multipliers (and 95% confidence intervals) to inform cascade genetic testing of diabetic family members

**Number of relatives test per true monogenic diabetes case identified**	Multipliers (and 95% CIs)	**Data source**
Relatives positive for monogenic diabetes	5.9 (5.4, 6.3)	Re-analysis of Shields et al[4] (specific to definition of modelled cohort)
Relatives negative for monogenic diabetes	0.4 (0.2, 0.6)	

For peer review only

Table 2E Pre-genetic test treatment pattern, cost and frequency of HBGM by true diagnosis

Diabetes type	Treatment	% receiving treatment	Mean monthly treatment costs	Mean frequency of HBGM[a]
Type 1	Insulin only	100%	£52	78
Type 2	Insulin only	36%	£55	43
	Insulin + tablets	54%	£50	43
	Tablets only	3%	£2	17
	No diabetes treatment	7%	£0	0
GCK mutation	Insulin only	87.5% (47.3%, 99.7%)	£10	63 (19, 107)
	Tablets only	12.5% (0.3%, 52.6%)	£1	0
HNF1A and *HNF4A* mutation	Insulin only	78.4% (61.8%, 90.2%)	£23	
	Insulin + tablets	13.5% (4.5%, 28.8%)	£16	76 (52, 99)
	Tablets	5.4% (0.1%, 18.2%)	£2	
	No diabetes treatment	2.7% (0.1%, 14.2%)	£0	0

[a] HBGM, home blood glucose monitoring

Table 2F Estimated dose and timing of future insulin requirements for individuals identified as having *HNF1A* or *HNF4A* mutations

Population	Expert 1		Expert 2	
	**Years after start of model**	**Insulin need (u)**	**Years after start of model**	**Insulin need (U/kg)**
Tablets only	0-19<br>20-24<br>25-29<br>≥30 yrs	As at model start<br>10 + tablets<br>20+ tablets<br>30 + tablets	0-9<br>10-14<br>15-24<br>≥2 yrs	As at model start<br>0.25 + tablets<br>0.4 + tablets<br>0.5 (no tablets)
Tablets and insulin	0-4<br>5-14<br>≥15 yrs	As at model start<br>20 + tablets<br>30 + tablets	0-9<br>10-14<br>≥15 yrs	As at start of model<br>0.4 + tablets<br>0.5 (no tablets)
Insulin only	0-9<br>10-24<br>≥25 yrs	As at model start<br>50<br>60	≥0 yrs	0.5

Table 2G Post-diagnosis HBGM frequency (95%CI) by treatment changed to and true diagnosis

	Time since diagnosis of monogenic diabetes (months)			
**Mutation - Treatment received**	**1**	**3 months**	**6 months**	**12 months**
GCK mutation – no diabetes treatment	0	0	0	0
HNF1/4A mutation – tablets only	50 (27, 73)	36 (14, 57)	22 (11, 33)	21 (10, 32)
HNF1/4A mutation – insulin and tablets	89 (56, 121)	66 (44, 87)	70 (46, 93)	43 (25, 60)

For peer review only

Table 2H Justification of parameter values and variations used in base case and sensitivity analyses

Parameter	Base case justification	Justification of sensitivity/threshold analyses
Prevalence of monogenic diabetes	In the accompanying clinical study, the total number of cases of monogenic diabetes was 34 from a total of 1407 individuals screened. This leads to an estimated prevalence within the definition of Cohort 1 of 34/1407 = 2·4%.	Although the total screened population was 1407 in the accompanying clinical study[1], the total eligible population in the defined geographical area was 2288. We could therefore assume:<br>1. that no more cases would have been found in the remaining eligible population not screened, i.e. the remaining 881 were not screened as they were quite obviously ***not*** cases of monogenic diabetes, therefore a lower estimate of the prevalence of monogenic diabetes might be appropriate (34/2288 = 1·5%),<br>2. there were no differences between those not screened and those who were screened, and so the base case numbers would not change (34/1407 = 2·4%)<br>3. those 881 who did not complete screening were ***more*** likely to be cases of monogenic diabetes. As an upper estimate, we assume the prevalence of monogenic diabetes in the defined cohort is doubled (68/1407 = 4·8%).<br>To investigate an increase or decrease in the prevalence of monogenic diabetes, sensitivity analyses assumed scenarios 1 and 3 above.
Sensitivity and specificity of the Ad Hoc Testing strategy	Based on referral rate data for Northern Ireland (the region with the lowest referral rates)[4]	Sensitivity analyses were based on all regions analysed by Shields et al[4]
Sensitivity of UCPCR test	Based on data from Besser et al[5] which used a prevalent case-control diagnostic study design: 0.94.	Since the sensitivity estimate for the UCPCR test is from a case-control diagnostic study, it is likely that the reported estimate will be greater than in practice.<br><br>Threshold analyses assumed sensitivity estimates for the UCPCR test between 1 and 0·55 (in 0.05 decrements). Results assuming a sensitivity of 1 or 0.55 are presented.
Specificity of UCPCR test	Based on data from Besser et al[5] which used a prevalent case-control diagnostic study design: 0.96.	Since the specificity estimate for the UCPCR test is from a case-control diagnostic study, it is likely that the reported estimate will be greater than in practice.<br><br>Threshold analyses assumed specificity estimates for the UCPCR test between 1 and 0·55 (in 0.05 decrements). Results assuming a specificity of 1 or 0.55 are shown.
Sensitivity of autoantibody test	Based on data from MacDonald et al[6] which used a prevalent case-control diagnostic study design: 0.99.	Since the sensitivity estimate for the autoantibody test is from a case-control diagnostic study, it is likely that the reported estimate will be greater than in practice.<br><br>Threshold analyses assumed sensitivity estimates for the autoantibody test between 1 and 0·55 (in 0.05 decrements).<br>Results assuming a sensitivity of 1 or 0.55 are shown.

For peer review only

Specificity of autoantibody test	Based on data from MacDonald et al[6] which used a prevalent case-control diagnostic study design: 0.82.	Since the specificity estimate for the autoantibody test is from a case-control diagnostic study, it is likely that the reported estimate will be greater than in practice.<br><br>Threshold analyses assumed specificity estimates for the autoantibody test between 1 and 0·55 (in 0.05 decrements).<br>Results assuming a sensitivity of 1 or 0.55 are shown.
Uptake of UCPCR test	Based on data from the accompanying clinical study which investigated the application of the Biomarker strategy.<br>Uptake of UCPCR was assumed to be 88%.	Threshold analyses where UCPCR test uptake was assumed to range from 100% to just 10% (in 10% decrements).<br>It was hypothesised that test uptake in practice is likely to be lower than test uptake in the accompanying clinical study where individuals have consented to participating in a study.<br><br>Results of assumptions that uptake of UCPCR is 100% or 10% are reported.
Uptake of autoantibody test	Based on data from the accompanying clinical study which investigated the application of the Biomarker strategy.<br>Uptake of autoantibody testing was assumed to be 92%.	Threshold analyses where autoantibody test uptake was assumed to range from 100% to just 10% (in 10% decrements).<br><br>It was hypothesised that test uptake in practice is likely to be lower than test uptake in the accompanying clinical study where individuals have consented to participating in a study.<br><br>Results of assumptions that uptake of autoantibody testing is 100% or 10% are reported.
Uptake of genetic test	Based on data from the accompanying clinical study which investigated the application of the Biomarker strategy.<br>Uptake of genetic testing was assumed to be the same as for autoantibody testing (92%) since the same blood sample for autoantibody testing was used for the genetic testing.	Threshold analyses where genetic test uptake was assumed to range from 100% to just 10% (in 10% decrements).<br><br>It was hypothesised that test uptake in practice is likely to be lower than test uptake in the accompanying clinical study where individuals have consented to participating in a study.<br><br>Results of assumptions that uptake of genetic testing is 100% or 10% are reported.
Repeat urine samples and UCPCR tests	Based on data from the accompanying clinical study which investigated the application of the Biomarker strategy. The percentage of repeat urine samples and UCPCR tests was assumed to be 2%.	Threshold analyses were undertaken assuming no repeats, 1%, 5%, 10%, 20%, 50%, 100%, 150% and 200% of samples and tests needed to be repeated. 200% repeat samples and tests can be interpreted as every individual requiring another 2 urine samples and UCPCR tests to be done, so that in total every individual has provided 3 urine samples and 3 UCPCR tests have been done – an extreme assumption.<br><br>Results for assuming 200% repeat samples and tests are presented.
Repeat blood samples and	Based on data from the accompanying clinical study which investigated the	Threshold analyses were undertaken assuming no repeats, 1%, 5%, 10%, 20%, 50%, 100%, 150% and 200% of samples and tests needed to be repeated. 200%

autoantibody tests	application of the Biomarker strategy. The percentage of repeat blood samples and autoantibody tests was assumed to be 2%.	repeat samples and tests can be interpreted as every individual requiring another 2 blood samples and autoantibody tests to be done, so that in total every individual has provided 3 blood samples and 3 autoantibody tests have been done, clearly an extreme assumption.<br><br>Results for assuming 200% repeat samples and tests are presented.
Percentage of individuals with *GCK* mutation who are receiving insulin treatment at the start of the model	Based on data from the accompanying clinical study which investigated the application of the Biomarker strategy. 88% of individuals with *GCK* mutation are receiving insulin treatment at the start of the model, while 12% are receiving tablets (metformin and sulphonylureas).	Threshold analyses assuming 100% to 10% (in 10% decrements) of individuals with *GCK* mutations are receiving insulin at the start of the model.<br><br>Results from assuming 100% or 10% are receiving insulin at the start of the model are presented.
Percentage of individuals with *HNF1A* or *HNF4A* mutation who are receiving insulin treatment at the start of the model	Based on data from the accompanying clinical study which investigated the application of the Biomarker strategy. 78% of individuals with *HNF1A* or *HNF4A* mutation are receiving insulin treatment at the start of the model, 5% are receiving insulin and tablets (metformin and sulphonylureas), 14% are receiving tablets and 3% are not treated pharmacologically.	Threshold analyses assuming 100% to 10% (in 10% decrements) of individuals with *HNF1A* or *HNF4A* mutations are receiving insulin at the start of the model.<br><br>Results from assuming 100% or 10% are receiving insulin at the start of the model are presented.
Genetic test cost	UK referral centre costs[7]: £350 for *GCK* mutation; £450 for *HNF1A* and *HNF4A* mutations.	Threshold analyses were conducted to identify at what cost of genetic tests would the All Tested strategy incur no additional costs over the No Testing strategy. Costs of tests for GCK and HNF1A and HNF4A mutations were reduced in 10% steps to just 10% of their base case costs: £35 for GCK and £45 for HNF1A and HNF4A.<br><br>Results of assumptions that genetic costs are 100% or 10% of their current costs are reported.
Long-term insulin need for individuals with *HNF1A* or *HNF4A* mutations	Expert 1	Expert 2, who assumed a larger dose of insulin would generally be required sooner than that stated by Expert 1.
Percentage of individuals with *HNF1A* or *HNF4A* mutations who remain on most appropriate treatment after a diagnosis of	Based on data from the accompanying clinical study which investigated the application of the Biomarker strategy. At 1 and 3 months after changing to more appropriate treatment, 86% are receiving tablets only (sulphonylureas and	The base case estimates are based on a small number of participants. Threshold analyses have been conducted to investigate the percentage of individuals with *HNF1A* or *HNF4A* mutations who need to remain on tablets for the strategies to be cost-saving compared to No Testing.

For peer review only

monogenic diabetes	metformin). At 6 and 12 months 89% and 77% are on tablets only, respectively.	It was assume that for all follow-up time periods after a monogenic diabetes diagnosis, the percentage receiving tablets is: 100%, 50%, 25% or 10%.<br><br>Results assuming 100% and 10% receive tablets are presented.
Cascade family testing	Analysis of referral rate data[7] indicate that for every 10 case of monogenic diabetes identified, 6.3 family members are also genetically tested: with 5.9 being positive for monogenic diabetes and 0.4 being negative for monogenic diabetes.	The impact of family cascade testing in the Ad Hoc, Clinical Prediction Model and Biomarker strategies was investigated by removing all cascade family testing from the strategies.<br><br>Estimates of the magnitude of cascade family testing based on the upper 95% confidence interval limits are used where 6.3 family members are found to be positive for monogenic diabetes, and 0.6 are found to be negative for monogenic diabetes, compared to the scenario where there is no family testing.
Frequency of HBGM before and after changing treatment due to a diagnosis of monogenic diabetes	Based on data from the accompanying clinical study which investigated the application of the Biomarker strategy. Data suggested that individuals with *GCK* mutations stopped HBGM after their diagnosis of monogenic diabetes, while individuals with *HNF1A* or *HNF4A* mutations significantly reduced their frequency of HBGM after a diagnosis of monogenic diabetes.	The 95% confidence limits for the estimated frequency of HBGM at the start of the model and at follow-up after a treatment change for individuals with *HNF1A* or *HNF4A* mutations were used in sensitivity analyses. The change in frequency of HBGM before and after a diagnosis of monogenic diabetes was maximised (which would favour strategies to identify cases of monogenic diabetes) by assuming the upper 95% confidence limit at baseline and the lower 95% confidence limits at follow-up. Conversely, the change in frequency of HBGM was minimised (which would not be as favourable to strategies to identify cases of monogenic diabetes) by assuming the lower 95% confidence limit at baseline and the upper 95% confidence limit at follow-up.

UCPCR, urinary c-peptide to creatinine ratio; HBGM, home blood glucose monitoring

For peer review only

Fig 2A Incremental costs (vs No Testing) for all strategies for reducing percentage of *GCK* cohort starting on insulin

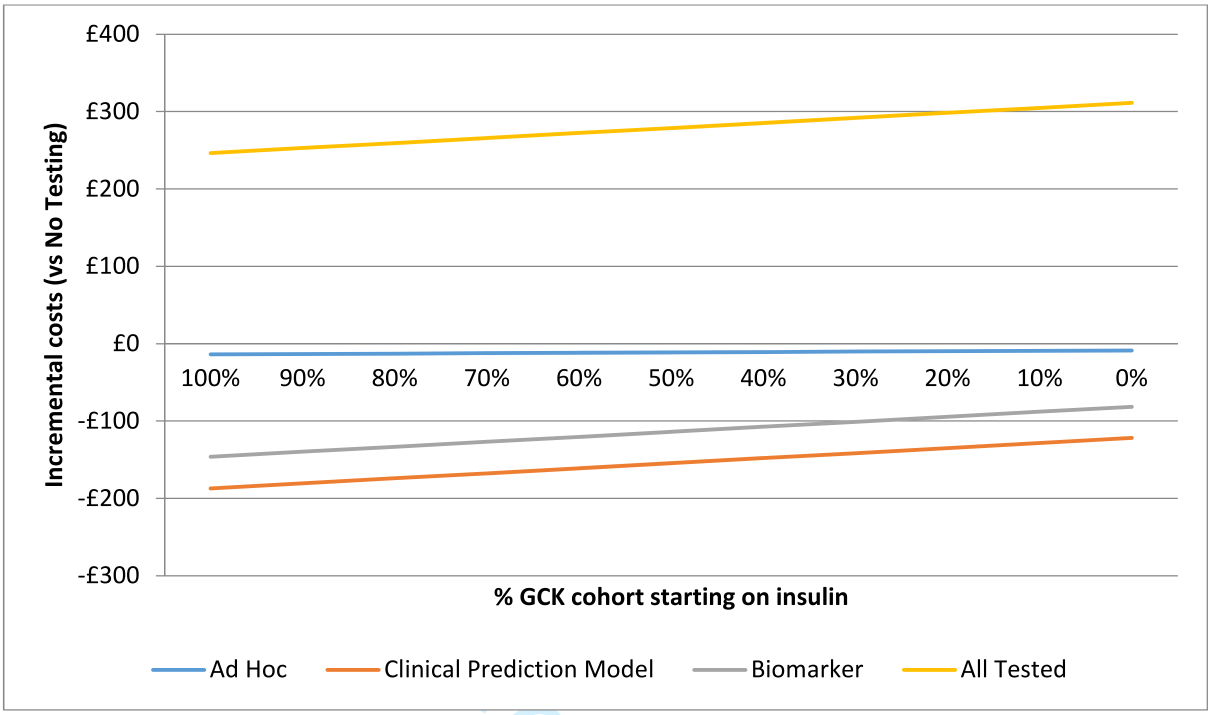

Fig 2B Incremental costs (vs No Testing) for all strategies for reducing percentage of *HNF1A* and *HNF4A* cohort starting on insulin

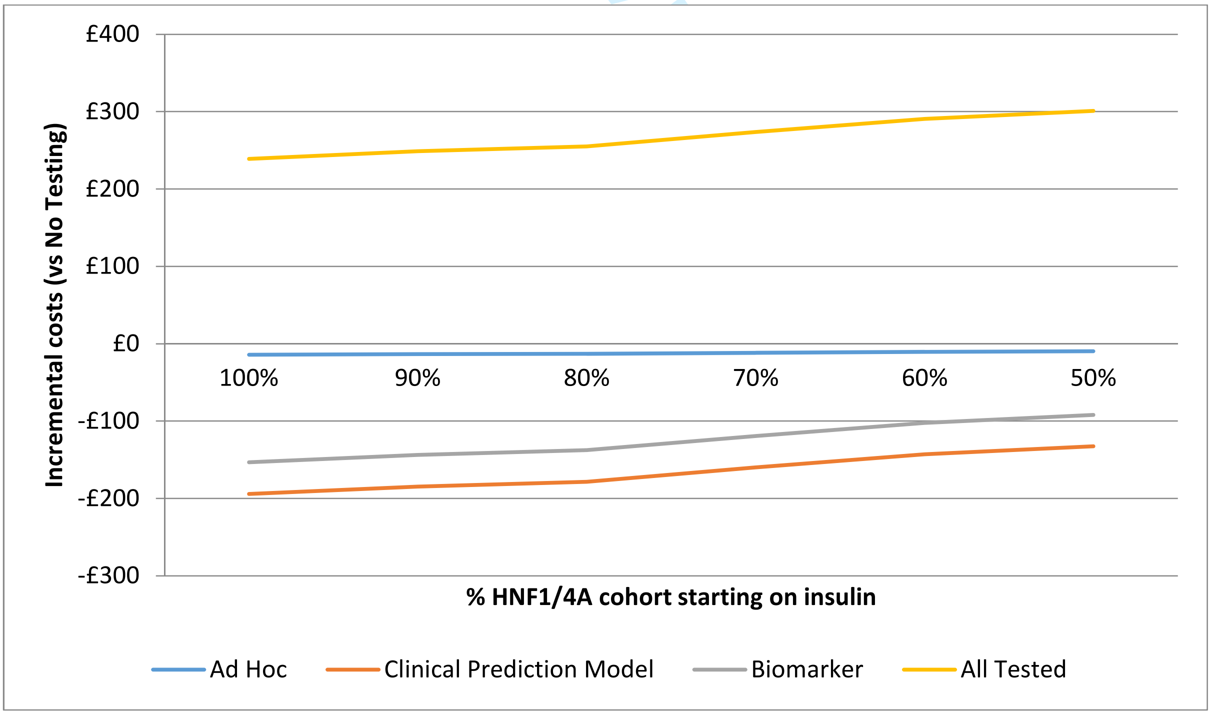

Fig 2C Incremental costs (vs No Testing) for the Biomarker Testing strategy with reducing levels of UCPCR and antibody testing uptake

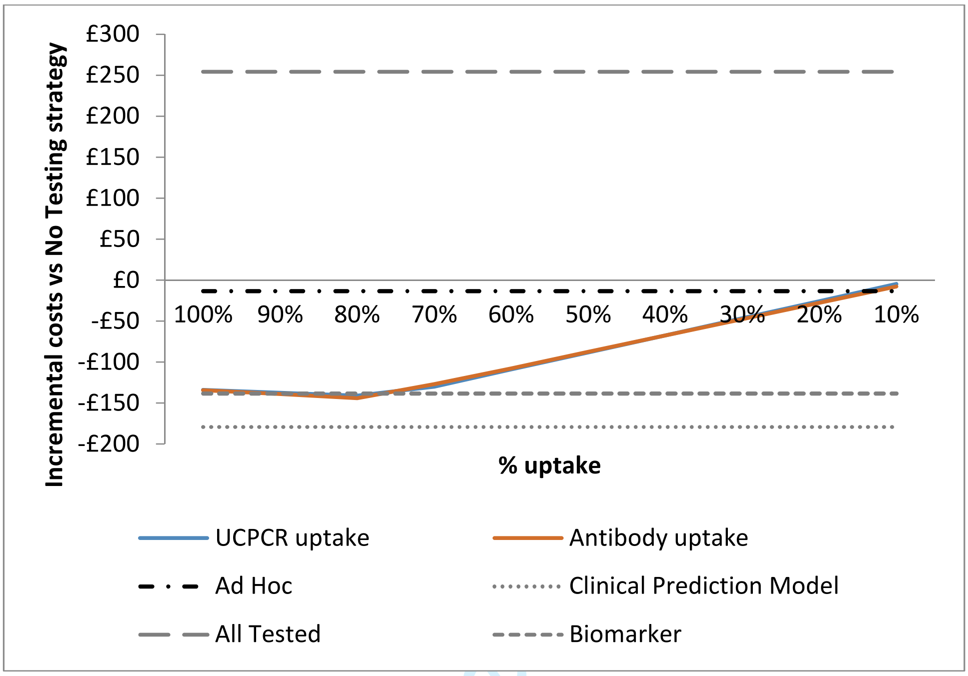

Fig 2D Incremental costs (vs No Testing) for the Biomarker Testing strategy with reducing estimates of sensitivity and specificity for the UCPCR and antibody tests

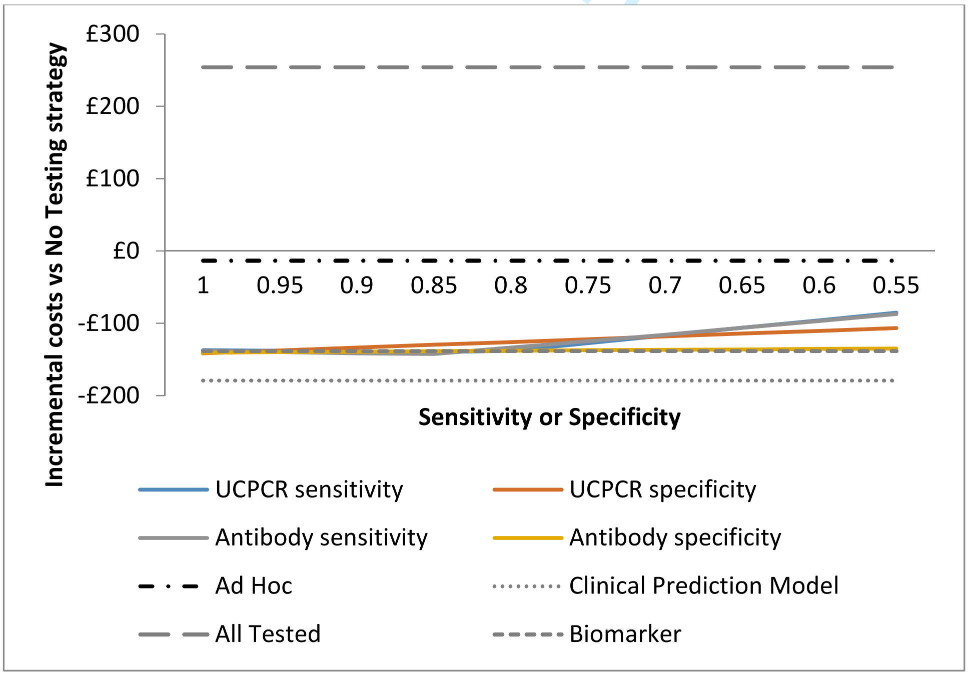

Fig 2E Incremental costs for the Biomarker Testing strategy (vs No Testing) with increasing estimates of repeat samples and UCPCR and autoantibody tests

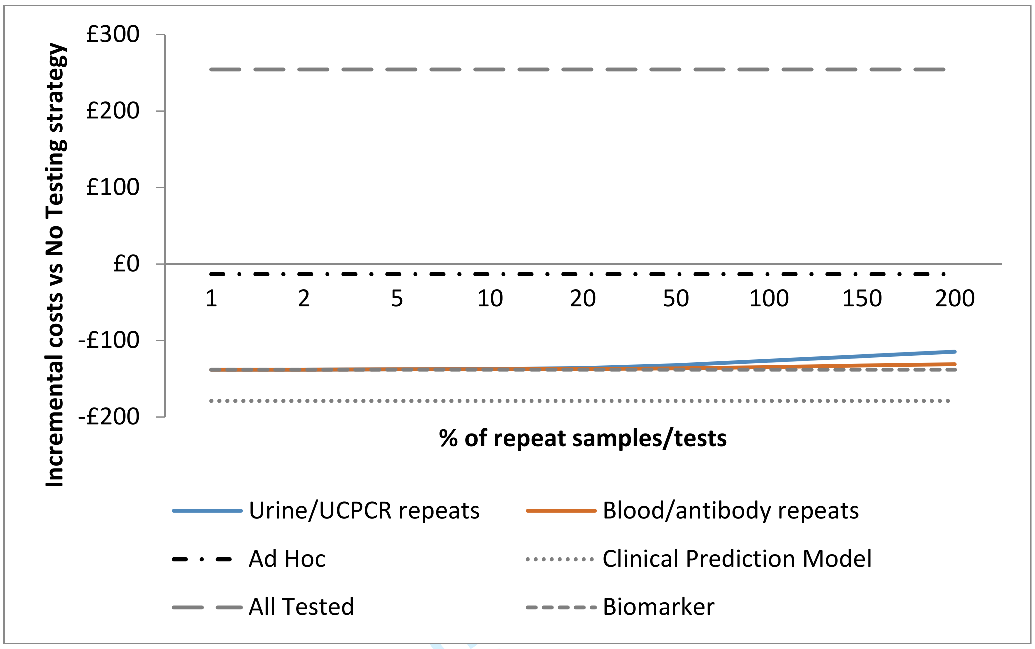

Fig 2F Incremental costs (vs No Testing) for all strategies when genetic test costs are reduced

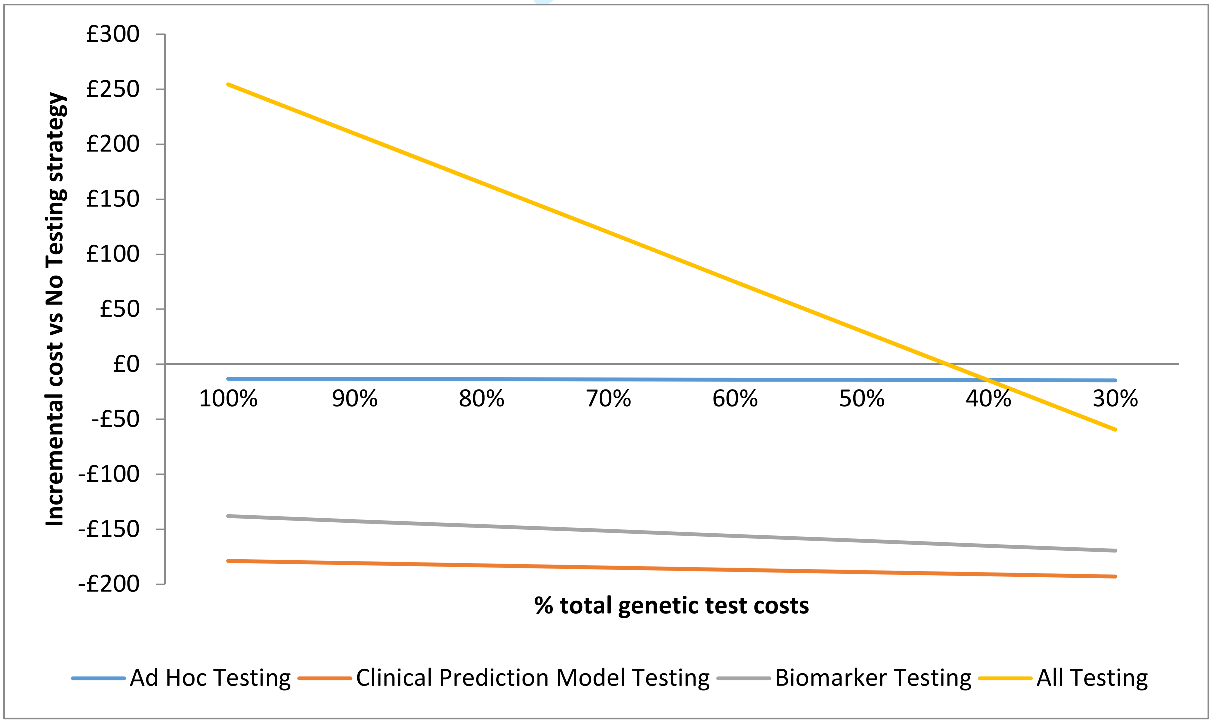

Utility improvement sensitivity analysis

In this sensitivity analysis it was assumed that individuals with HNF1A and HNF4A mutations who successfully transferred to sulphonylureas experienced an improvement in utility of 0.02 from one year after changing treatment (based on data from the associated clinical study). Please note that these analyses were run on an updated version of CORE (v9.0 rather than v8.5, as v8.5 no longer available). The total costs and QALYs are different, but importantly the incremental costs are the same as the results from v8.5.

Table 2I. Results of assuming improved utility for those successfully changing to sulphonylureas

Strategy	Total undiscounted costs [a]	Total discounted costs [a]	Incremental costs vs No Testing strategy [a]	Total discounted QALYs	Incremental QALYs vs No Testing strategy	% who are genetically tested		ICER vs No Testing [a]
						With monogenic diabetes	Without monogenic diabetes	
Clinical Prediction Model Testing[b]	£133,200	£65,900	-£100	10.3865	0.0013	92	3	-£111,700
Biomarker Testing	£133,300	£65,900	-£100	10.3865	0.0013	92	8	-£80,500
Ad Hoc Testing	£133,500	£66,000	0	10.3853	<0.001	6	<1	-£103,400
No Testing	£133,600	£66,000	NA	10.3852	NA	0	0	NA
All Testing	£133,700	£66,300	£300	10.3865	0.0013	92	92	£225,700

[a] rounded to nearest £100.

For peer review only - http://bmjopen.bmj.com/site/about/guidelines.xhtml

The total discounted QALYs for the Clinical Prediction Model, Biomarker and All Testing strategies are all the same (10.3865). This is because a maximum proportion of individuals with MODY are assumed to accept testing (92%), which is the case for these three strategies. The assumed proportion of individuals with HNF1A or HNF4A mutations who successfully change treatment (100%) does not depend on the testing strategy used. Thus, there is no difference in the proportion of people with HNF1A and HNF4A mutations who successfully change treatment between these three strategies, and so the total QALYs are the same. It is the relative costs of the strategies which allows some distinction between the Clinical Prediction Model, Biomarker and All Testing strategies.

For instance, the results suggest that the All Testing strategy would not be considered cost-effective by NICE willingness to pay per QALY gained thresholds (of £20,000 to £30,000). This is because it is estimated to cost £300 more, and produce a utility incremental of 0.0013 over the No Testing strategy, giving an ICER of £225,700.

As the ICERs for the Ad Hoc, Clinical Prediction Model and Biomarker Testing strategies are all estimated to cost less but produce more QALYs than the No Testing strategy (Fig X), there are all considered to be cost-effective options.

In a fully incremental analysis, the Clinical Prediction Model is considered to be the most cost-effective strategy – it produces the most QALYs at the least cost.

Fig 2G Cost-effectiveness plane for the sensitivity analysis which assumes an improvement in utility of 0.02 for those with HNF1A and HNF4A who successfully change treatment

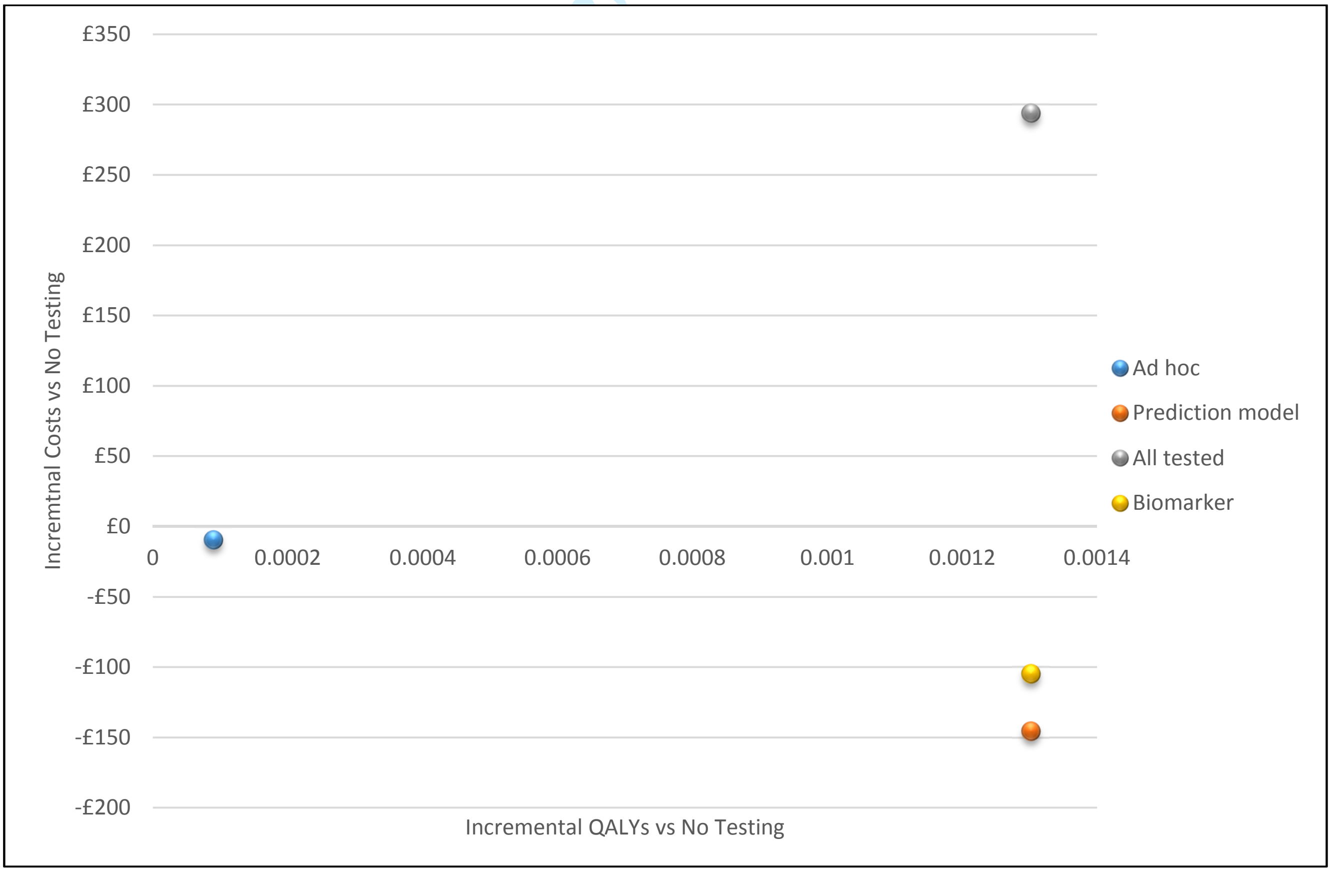

For peer review only - http://bmjopen.bmj.com/site/about/guidelines.xhtml

References

1. Shields BM, Shepherd M, Hudson M, et al. Population-Based Assessment of a Biomarker-Based Screening Pathway to Aid Diagnosis of Monogenic Diabetes in Young-Onset Patients. *Diabetes Care* 2017;40(8):1017-25. doi: 10.2337/dc17-0224 [published Online First: 2017/07/14]
2. Llaurado G, Gonzalez-Clemente J-M, Maymo-Masip E, et al. Serum levels of TWEAK and scavenger receptor CD163 in type 1 diabetes mellitus: relationship with cardiovascular risk factors. A case-control study. *PLoS One* 2012;7(8):e43919.
3. Office for National Statistics. Population Estimates by Ethnic Group 2002-2009. Statistical Bulletin, 2011.
4. Shields BM, Hicks S, Shepherd MH, et al. Maturity-onset diabetes of the young (MODY): how many cases are we missing? *Diabetologia* 2010;53:2504-08.
5. Besser REJ, Shepherd MH, McDonald TJ, et al. Urinary c-peptide-to-creatinine ratio is a practical outpatient tool for identifying hepatocyte nuclear factor 1-α/hepatocyte nuclear factor 4-α maturity-onset diabetes of the young from long-duration type 1 diabetes. *Diabetes Care* 2011;34:1-6.
6. McDonald TJ, Colclough K, Brown R, et al. Islet autoantibodies can discriminate maturity-onset diabetes of the young (MODY) from type 1 diabetes. *Diabetic Medicine* 2011;28:1028-33.
7. Royal Devon and Exeter NHS Foundation Trust. [Available from: http://www.rdehospital.nhs.uk/prof/molecular_genetics/tests/Full_Test_List.htm accessed 13th October 2014.

For peer review only - http://bmjopen.bmj.com/site/about/guidelines.xhtml

Supplementary Data 3: Test-related parameters

Table 3A Summary of the tests involved and estimates of sensitivity and specificity used in the economic evaluation

Test-treatment strategy	Tests used	Sensitivity	Specificity	Data sources
Ad Hoc Testing	Clinical referral based on patient characteristics	0·04	0·996	Shields et al[1]; 2011 census data; Clinical study; Unpublished prevalence data
	Genetic test	1	1	Assumption
Clinical Prediction Model Testing	Type 1 clinical prediction model	0·5 - 0·96	0·65 - 0·996	Shields et al[2]. Estimates of sensitivity and specificity depend on the combination of the probability thresholds used from both clinical prediction models.
	Type 2 clinical prediction model	0·8 - 0·99	0·73 - 0·99	Shields et al[2]. Estimates of sensitivity and specificity depend on the combination of the probability thresholds used from both clinical prediction models.
	Genetic test	1	1	Assumption
Biomarker Testing	UCPCR test	0·94	0·96	Besser et al[3]
	Autoantibody test	0·99	0·82	McDonald et al[4]
	Genetic test	1	1	Assumption
All Testing	Genetic test	1	1	Assumption

UCPCR, urinary c-peptide to creatinine ratio

For peer review only

For peer review only - http://bmjopen.bmj.com/site/about/guidelines.xhtml

Table 3B Sensitivity and specificity of the Ad Hoc Testing strategy by regions in the UK

Region	Sensitivity	Specificity
Northern Ireland[a]	0.038	0.996
Wales	0.044	0.998
Scotland	0.132	0.988
England	0.086	0.993
South West England	0.196	0.977
South East England	0.080	0.995
London	0.049	0.995
East England	0.060	0.996
West Midlands England	0.077	0.994
East Midlands England	0.074	0.995
Yorkshire/Humberside England	0.084	0.996
North East England	0.122	0.994
North West England	0.074	0.995
UK	0.087	0.993
England and Wales	0.084	0.993

[a]Used in base case analysis

References

1. Shields BM, Hicks S, Shepherd MH, et al. Maturity-onset diabetes of the young (MODY): how many cases are we missing? *Diabetologia* 2010;53:2504-08.
2. Shields BM, McDonald TJ, Ellard S, et al. The development and validation of a clinical prediction model to determine the probability of MODY in patients with young-onset diabetes. *Diabetologia* 2012 [published Online First: 5th January 2012]
3. Besser REJ, Shepherd MH, McDonald TJ, et al. Urinary c-peptide-to-creatinine ratio is a practical outpatient tool for identifying hepatocyte nuclear factor 1-α/hepatocyte nuclear factor 4-α maturity-onset diabetes of the young from long-duration type 1 diabetes. *Diabetes Care* 2011;34:1-6.
4. McDonald TJ, Colclough K, Brown R, et al. Islet autoantibodies can discriminate maturity-onset diabetes of the young (MODY) from type 1 diabetes. *Diabetic Medicine* 2011;28:1028-33.

For peer review only

Supplementary Data 4: Cost parameters

Table 4A Summary of the costs considered for each strategy

	**No Testing**	**Ad Hoc Testing**	**Clinical Prediction Model Testing**	**Biomarker Testing**	**All Testing**
Diabetes-specific consultations	o	o	o	o	o
Current treatment	o	o	o	o	o
HBGM on current treatment	o	o	o	o	o
Blood test (for genetic test or autoantibody testing)		o	o	o	o
UCPCR test				o	
Autoantibody test				o	
Genetic test		o	o	o	o
Treatment transfer assistance[a]		o	o	o	o
New treatment		o	o	o	o
HBGM on new treatment		o	o	o	o
Long-term management	o	o	o	o	o

[a]Includes telephone calls with nurse and visit(s) to GP for changes in treatment during 12 month follow-up. UCPCR, urinary c-peptide to creatinine ratio; HBGM, home blood glucose monitoring

Table 4B Costs of testing associated with the strategies

**Cost**	**Value (£, 2018)**	**Source**
GP nurse time for collecting blood sample	£6	10 minutes at £36 per 1hr GP nurse patient contact time[1]
Genetic test for GCK mutation	£350	Sanger sequence analysis from UK referral centre[2]
Genetic test for HNF1/4A mutation	£450	Sanger sequence analysis from UK referral centre[2]
Genetic test for known mutation	£100	Sanger sequence analysis from UK referral centre[2]
Nurse time for successful treatment transfer	£24	Four 10 minute phone calls (expert opinion) at £36 per 1hr GP nurse patient contact time[1]
GP time for informing patient of genetic test result and treatment change	£28	Cost of GP consultation[1]
UCPCR pack	£3·90	Postage
UCPCR test	£10·50	RD&E laboratory[2]
Autoantibody test	£20	RD&E laboratory[2]

UCPCR, urinary c-peptide to creatinine ratio

For peer review only - http://bmjopen.bmj.com/site/about/guidelines.xhtml

Table 4C Cost estimates (£, 2018) used in the IMS CDM model

Event	Cost (£, 2018)	Source
**CVD complications**		
Myocardial infarction (MI) in 1st year of MI	£7,550	Clarke[3]
Second and subsequent yrs after an MI	£1,250	Clarke[3]
Angina in 1st year of angina	£250	Ward[4]
Second and subsequent yrs after an angina	£200	Ward[4]
Congestive heart failure (CHF) in 1st year of CHF	£3,500	Clarke[5]
Second and subsequent yrs after a CHF	£500	Clarke[5]
Stroke in 1st year of stroke	£4,600	Clarke[3]
Second and subsequent yrs after a stroke	£850	Clarke[3]
Stroke death within 30 days of stroke	£6,350	Clarke[3]
Peripheral vascular disease (PVD) in 1st year of PVD	£1,150	Clarke [5]
Second and subsequent yrs after a PVD	£450	Clarke[5]
**Renal complications**		
Hemodialysis in 1st year of needing hemodialysis	£43,500	Baboolal[6]
Hemodialysis in second & subsequent yrs of needing hemodialysis	£43,500	Baboolal [6]
Peritoneal dialysis in 1st year of needingperitoneal dialysis	£24,250	Baboolal [6]
Peritoneal dialysis in second & subsequent yrs of needing peritoneal dialysis	£24,250	Baboolal [6]
Renal transplant in 1st year of needing renal transplant	£13,100	NHS Schedule Reference costs[7]; Wight [8]
Renal transplant in second & subsequent yrs of needing renal transplant	£7,050	Wight[8]
**Acute events**		
Major hypoglyceamic event	£200	Hammer[9]
Minor hypoglyceamic event	£0	Would not require medical assistance
Ketoacidosis event	£1,250	Scuffham[10]
Lactic acid event	£2,500	Curtis[11]
Edema onset	£50	Curtis[11]
Edema follow-up	£0	Assume no follow-up
**Eye disease**		
Laser treatment	£100	NHS Schedule Reference costs[7]
Cataract operation	£800	NHS Schedule Reference costs[7]
Following cataract operation	£550	Clarke[3]
Blindness in the yr of onset	£7,250	Mitchell[12]
Blindness in the following yrs	£7,250	Mitchell[12]
**Neuropathy/foot ulcer**		
Neuropathy in the first yr	£150	BNF[13]
Neuropathy in subsequent yrs	£150	BNF[13]
Amputation (one-off cost)	£7,950	Kerr[14]

For peer review only - http://bmjopen.bmj.com/site/about/guidelines.xhtml

Amputation prosthesis (one-off cost)	£3,200	Kerr [14]
Gangrene treatment	£2,700	
After a healed ulcer	£0	Assumption
Infected ulcer	£4,050	Kerr [14]
Standard uninfected ulcer	£4,050	Kerr [14]
Healed ulcer in those with an amputation history	£0	Assumption
**Other**		
Statins	£0	NICE guidance and BNF[13]
Aspirin	£0	NICE guidance and BNF[13]
Angiotensin-converting enzyme (ACE)	£0	BNF
Screening for microalbuminuria	£0	NICE[15]
Screening for gross proteinuria	£0	Assume as for MA
Stopping ACEs due to side effects	£0	Assumptions
Eye screening	£50	NICE [15]
Foot screening programme	£100	NICE[16] and Curtis [17]
Non-standard ulcer treatment (e.g. Regranex)	£0	Assumptions
Anti-depression treatment	£0	Assumptions
Screening for depression	£0	Assumptions

Table 4D Annual number of primary care consultations (taken from Currie et al 2010[18])

**Type of consultation**	**Type 1**	**Type 2**	**Type 1 control**	**Type 2 control**	**Cost per consultation**
GP surgery	7·3	8·7	4·5	5·4	£34
GP home visit	0·3	0·6	0·1	0·4	£41
GP telephone	0·5	0·7	0·3	0·4	£20
Community nurse clinic	0·9	1·5	0·3	0·6	£12
Total cost	£278	£349	£165	£213	
Additional cost over controls	£113	£136			

For peer review only

For peer review only - http://bmjopen.bmj.com/site/about/guidelines.xhtml

References

1. Curtis LA, Burns A. Unit costs of health and social care 2017: Personal Social Services Research Unit, University of Kent, 2017.
2. Royal Devon and Exeter NHS Foundation Trust. [Available from: http://www.rdehospital.nhs.uk/prof/molecular_genetics/tests/Full_Test_List.htm accessed 13th October 2014.
3. Clarke P, Gray A, Legood R, et al. The impact of diabetes-related complications on healthcare costs: results from the United Kingdom Prospective Diabetes Study (UKPDS 65). *Diabetic Medicine* 2003;20:442-50.
4. Ward S, Lloyd Jones M, Pandor A, et al. Statins for the prevention of coronary events. Technology assessment report commissioned by the HTA programme on behalf of the National Institute for Clinical Excellence, 2005.
5. Clarke PM, Glasziou P, Patel A, et al. Event rates, hospital utilization, and costs associated with major complications of diabetes: a multicountry comparative analysis. *PLoS Medicine* 2010;7(2)
6. Baboolal K, McEwan P, Sondhi S, et al. The cost of renal dialysis in a UK setting - a multicentre study. *Nephrology Dialysis Transplantation* 2008;23(6):1982-89.
7. Department of Health. National Schedule of Reference Costs 2012-2013, 2013.
8. Wight J, Chilcott J, Holmes M, et al. The clinical and cost-effectiveness of pulsatile machine perfusion versus cold storage of kidneys for transplantation retrieved from heart-beating and non-heart-beating donors. *Health Technology Assessment* 2003;7(25)
9. Hammer M, Lammert M, Mejias SM, et al. Costs of managing severe hypoglycaemia in three European countries. *Journal of Medical Economics* 2009;12(4)
10. Scuffham P, Carr L. The cost-effectiveness of continuous subcutaneous insulin infusion compared with multiple daily injections for the management of diabetes. *Diabetic Medicine* 2003;20(7):586-93.
11. Curtis L. Unit Costs of Health and Social Care 2013, 2013.
12. Mitchell P, Annemans L, Gallagher M, et al. Cost-effectiveness of ranibizumab in treatment of diabetic macular oedema (DME) causing vision impairment: evidence from the RESTORE trial. *British Journal of Opthalmology* 2011;early online
13. British National Formulary 67. BNF 67 (May 2014) 2014 [
14. Kerr M. Foot care for people with diabetes: the economic case for change: NHS Diabetes, 2012.
15. National Institute for Health and Clinical Excellence. Quality Standards Programme. NICE cost impact and commissioning assessment for diabetes in adults, 2011.
16. National Institute for Health and Clinical Excellence. Diabetes footcare commissioning and benchmarking tool, 2012.
17. Curtis L. Unit Costs of Health and Social Care 2011: PSSRU, 2012.
18. Currie CJ, Gale EAM, Poole CD. Estimation of primary care treatment costs and treatment efficacy for people with type 1 and Type 2 diabetes in the United Kingdom from 1997 to 2007. *Diabetic Medicine* 2010;27(8):938-48.

For peer review only - http://bmjopen.bmj.com/site/about/guidelines.xhtml

Section/item	Recommendation	Reported on page
**Title and abstract**		
Title	Identify the study as an economic evaluation or use more specific terms such as "cost-effectiveness analysis", and describe the interventions compared	1
Abstract	Provide a structured summary of objectives, perspective, setting, methods (including study design and inputs), results (including base case and uncertainty analyses), and conclusions.	2
**Introduction**		
Background and objectives	Provide an explicit statement of the broader context for the study.<br>Present the study question and its relevance for health policy or practice decisions.	5-6
**Methods**		
Target population and subgroups	Describe characteristics of the base case population and subgroups analysed, including why they were chosen.	9-10
Setting and location	State relevant aspects of the system(s) in which the decision(s) need(s) to be made.	7
Study perspective	Describe the perspective of the study and relate this to the costs being evaluated.	14
Comparators	Describe the interventions or strategies being compared and state why they were chosen.	7-9
Time horizon	State the time horizon(s) over which costs and consequences are being evaluated and say why appropriate	7
Discount rate	Report the choice of discount rate(s) used for costs and outcomes and say why appropriate.	15
Choice of health outcomes	Describe what outcomes were used as the measure(s) of benefit in the evaluation and their relevance for the type of analysis performed.	15
Measurement of effectiveness	*Single study-based estimates:* Describe fully the design features of the single effectiveness study and why the single study was a sufficient source of clinical effectiveness data.	
	Synthesis-based estimates: Describe fully the methods used for identification of included studies and synthesis of clinical effectiveness data.	10-12, 13
Measurement and valuation of preference based outcomes	If applicable, describe the population and methods used to elicit preferences for outcomes.	NA
Estimating resources and costs	*Single study-based economic evaluation:* Describe approaches used to estimate resource use associated with the alternative interventions. Describe primary or secondary research methods	

For peer review only - http://bmjopen.bmj.com/site/about/guidelines.xhtml

	for valuing each resource item in terms of its unit cost.<br>Describe any adjustments made to approximate to opportunity costs.	
	Model-based economic evaluation: Describe approaches and data sources used to estimate resource use associated with model health states. Describe primary or secondary research methods for valuing each resource item in terms of its unit cost. Describe any adjustments made to approximate to opportunity costs.	14-15
Currency, price date and conversion rate	Report the dates of the estimated resource quantities and unit costs. Describe methods for adjusting estimated unit costs to the year of reported costs if necessary. Describe methods for converting costs into a common currency base and the exchange rate.	14
Choice of model	Describe and give reasons for the specific type of decision analytical model used. Providing a figure to show model structure is strongly recommended	7
Assumptions	Describe all structural or other assumptions underpinning the decision-analytical model.	7-9, 12, 15
Analytical methods	Describe all analytical methods supporting the evaluation. This could include methods for dealing with skewed, missing, or censored data; extrapolation methods; methods for pooling data; approaches to validate or make adjustments (such as half cycle corrections) to a model; and methods for handling population heterogeneity and uncertainty.	10, 16
**Results**		
Study parameters	Report the values, ranges, references, and, if used, probability distributions for all parameters. Report reasons or sources for distributions used to represent uncertainty where appropriate. Providing a table to show the input values is strongly recommended.	16
Incremental costs and outcomes	For each intervention, report mean values for the main categories of estimated costs and outcomes of interest, as well as mean differences between the comparator groups. If applicable, report incremental cost-effectiveness ratios.	16-18
Characterising uncertainty	*Single study-based economic evaluation:* Describe the effects of sampling uncertainty for the estimated incremental cost and incremental effectiveness parameters, together with the impact of methodological assumptions (such as discount rate, study perspective).	NA

For peer review only

For peer review only - http://bmjopen.bmj.com/site/about/guidelines.xhtml

	Model-based economic evaluation: Describe the effects on the results of uncertainty for all input parameters, and uncertainty related to the structure of the model and assumptions.	18-21
Characterising heterogeneity	If applicable, report differences in costs, outcomes, or cost effectiveness that can be explained by variations between subgroups of patients with different baseline characteristics or other observed variability in effects that are not reducible by more information.	21
**Discussion**		
Study findings, limitations, generalisability, and current knowledge	Summarise key study findings and describe how they support the conclusions reached. Discuss limitations and the generalisability of the findings and how the findings fit with current knowledge.	21-25
**Other**		
Source of funding	Describe how the study was funded and the role of the funder in the identification, design, conduct, and reporting of the analysis. Describe other non-monetary sources of support.	3
Conflicts of interest	Describe any potential for conflict of interest of study contributors in accordance with journal policy. In the absence of a journal policy, we recommend authors comply with International Committee of Medical Journal Editors recommendations.	26

For peer review only

For peer review only - http://bmjopen.bmj.com/site/about/guidelines.xhtml
